# A generalized logrank-type test for comparison of treatment regimes in sequential multiple assignment randomized trials

**DOI:** 10.1093/biomtc/ujae139

**Published:** 2024-11-30

**Authors:** Anastasios A Tsiatis, Marie Davidian

**Affiliations:** Department of Statistics, North Carolina State University, Raleigh, NC 27695-8203, United States; Department of Statistics, North Carolina State University, Raleigh, NC 27695-8203, United States

**Keywords:** augmented inverse probability weighting, dynamic treatment regime, estimating function, potential outcomes

## Abstract

The sequential multiple assignment randomized trial (SMART) is the ideal study design for the evaluation of multistage treatment regimes, which comprise sequential decision rules that recommend treatments for a patient at each of a series of decision points based on their evolving characteristics. A common goal is to compare the set of so-called embedded regimes represented in the design on the basis of a primary outcome of interest. In the study of chronic diseases and disorders, this outcome is often a time to an event, and a goal is to compare the distributions of the time-to-event outcome associated with each regime in the set. We present a general statistical framework in which we develop a logrank-type test for comparison of the survival distributions associated with regimes within a specified set based on the data from a SMART with an arbitrary number of stages that allows incorporation of covariate information to enhance efficiency and can also be used with data from an observational study. The framework provides clarification of the assumptions required to yield a principled test procedure, and the proposed test subsumes or offers an improved alternative to existing methods. We demonstrate performance of the methods in a suite of simulation studies. The methods are applied to a SMART in patients with acute promyelocytic leukemia.

## 1 INTRODUCTION

Clinicians caring for patients with chronic diseases and disorders make sequential treatment decisions at key points in a patient’s disease or disorder progression based on accrued information on the patient, with the goal of optimizing a long-term health outcome. A treatment regime is a sequence of decision rules, each rule corresponding to a key decision point and mapping the accrued information to a recommended treatment option from among those feasible for the patient; thus, treatment regimes formalize clinical decision-making. Sequential multiple assignment randomized trials (SMARTs) (Murphy, 2005) involve multiple stages of randomization, each stage corresponding to a key decision point, and are ideally suited to study of treatment regimes. SMARTs have been conducted in a range of areas (Almirall et al., 2014; Kidwell, 2014; Lorenzoni et al., 2023) and are of increasing interest in chronic disease research. The treatment options at any stage can depend on the subject’s past history, including previous treatments and responses to them, and the design defines a set of regimes, known as the SMART’s embedded regimes. Primary or secondary analyses often focus on comparison of all or a subset of the embedded regimes based on a health outcome of interest.

There is an extensive literature on methods for inference on treatment regimes, including those in a specified set, such as the embedded regimes in a SMART (Nahum-Shani and Almirall, 2019; Tsiatis et al., 2020). The majority focus on a continuous or discrete outcome and comparison of the mean outcomes that would be achieved if the entire patient population were to receive treatments according to the rules in each regime in the set. In many chronic diseases and disorders, the outcome is a time to an event, e.g., in cancer, disease-free or overall survival time, and, as in conventional (single-stage) clinical trials, interest instead is in comparison of the event-time (survival) distributions if the patient population were to receive treatments according to the rules in each regime. A complication in any study with a survival outcome is that the outcome may be censored for some subjects.

**FIGURE 1:**
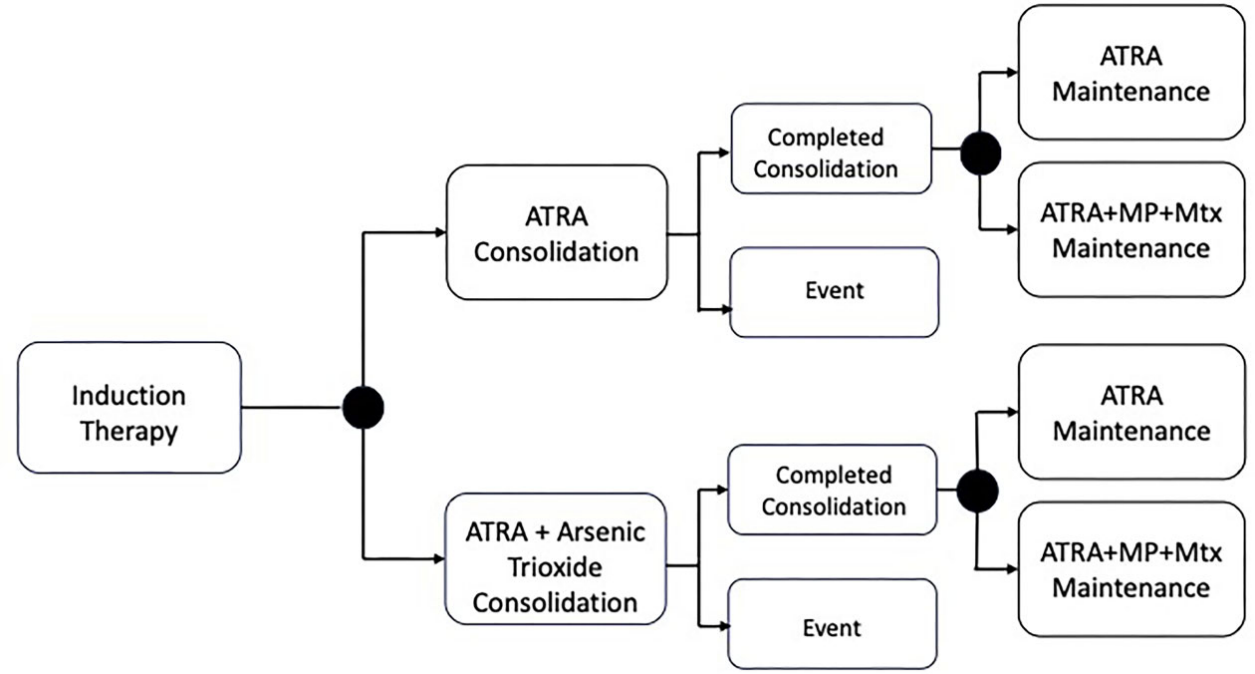
Schematic depicting the design of Study C9710. Solid circles represent points of randomization. The 4 embedded regimes implied by this design are of the form “Give consolidation therapy $a$ initially; if consolidation is completed before the event occurs, give maintenance therapy $b$,” where $a =$ ATRA or ATRA+Arsenic Trioxide and $b =$ ATRA or ATRA+MP+Mtx.

This situation is exemplified by North American Leukemia Intergroup Study C9710, coordinated by the Cancer and Leukemia Group B, now part of the Alliance for Clinical Trials in Oncology, in patients with acute promyelocytic leukemia (APL) (Powell et al., 2010). The primary outcome was event-free survival (EFS) time, a composite of time to failure to achieve complete remission (CR), relapse after CR, or death, whichever first. The study was a SMART with 2 stages as in Figure 1: All subjects received the same induction chemotherapy and were then randomized at stage 1 to one of 2 consolidation therapies, 2 courses of either all-trans-retinoic acid (ATRA) or ATRA combined with arsenic trioxide; thus, the first decision point corresponds to choice of consolidation therapy. Those not experiencing the event by completion of consolidation were to be randomized at stage 2 to receive maintenance therapy of ATRA alone or ATRA plus oral methotrexate (Mtx) and mercaptopurine (MP); thus, the second decision point involves choice of maintenance for patients completing consolidation. For some subjects, EFS time was not observed because it did not occur during the study period, i.e., was administratively censored, or was censored due to loss to follow up. Subjects for whom the event occurred or was censored during consolidation were not re-randomized. As shown in Figure 1, Study C9710 involves 4 embedded regimes; although the goal was not to compare these regimes, the trial presents the opportunity for insight on how best to sequence these therapies in the treatment of APL.

With a time-to-event outcome, several methods for comparing regimes in a specified set have been proposed, on the basis of survival probabilities at a single time point (Lunceford et al., 2002) or via logrank-type tests comparing survival distributions for 2 regimes that assign different stage 1 treatments. (Guo and Tsiatis, 2005; Feng and Wahed, 2008; Li and Murphy, 2011). Kidwell and Wahed (2013) and Li et al. (2014) propose tests for comparing $\ge 2$ regimes, including “shared path” regimes with the same stage 1 treatment. Both methods have potential shortcomings, discussed in the sequel.

In this article, we propose a general framework from which to derive a logrank-type test for comparing the survival distributions for an arbitrary set of regimes for any number of decision points; e.g., in a SMART, all or a subset of the embedded regimes. In addition to the basic test, the framework also yields a covariate-adjusted test statistic that uses baseline and intermediate covariate information to yield more powerful tests. Although we focus on SMARTs, the methodology also can be used with observational data and includes the approach of Li et al. (2014). In Section 2, we present the framework and test procedures and constrast our approach to existing methods. We present simulation studies demonstrating performance in Section 3 and apply the methods to Study C9710 in Section 4.

## 2 STATISTICAL FRAMEWORK AND METHODOLOGY

### 2.1 Treatment regimes with time-to-event outcome

We first follow Tsiatis et al. (2020, Section 8.3.1) and characterize a $K$-decision treatment regime when the outcome is a time to an event, where at Decision $k$, $k=1,\ldots ,K$, $\mathcal {A}_k$ is the finite set of all treatment options. As in Table 1, for an individual, let $(\tau _1,{\boldsymbol x}_1, a_1)$ be the time of Decision 1 ($\tau _1=0$, baseline), information available, and treatment option in $\mathcal {A}_1$ given at Decision 1, respectively. For $k=2,\ldots ,K$, if the individual does not experience the event between Decisions $k-1$ and $k$, let $(\tau _k, {\boldsymbol x}_k, a_k)$, denote the time Decision $k$ is reached, additional information obtained between Decisions $k-1$ and $k$, and the treatment option in $\mathcal {A}_k$ given at Decision $k$. Then, as in Table 1, let ${\boldsymbol h}_k$, $k=1,\ldots ,K$, denote the history available on an individual at Decision $k$. A treatment regime $d$ is a set of decision rules $d = \lbrace d_1({\boldsymbol h}_1),\ldots , d_K({\boldsymbol h}_K)\rbrace = (d_1,\ldots ,d_K)$, where, for an individual for whom ${\boldsymbol h}_k$ indicates the event has not yet occurred, the $k$th rule $d_k({\boldsymbol h}_k)$ returns a treatment option in the subset $\Psi _k({\boldsymbol h}_k) \subseteq \mathcal {A}_k$ that is feasible for an individual with history ${\boldsymbol h}_k$. If ${\boldsymbol h}_k$ indicates that the event has already occurred, $d_k({\boldsymbol h}_k)$ makes no selection, and $\Psi _k({\boldsymbol h}_k) = \emptyset$. As in Tsiatis et al. (2020, Section 6.2.2), at Decision $k$ there may be $\ell _k$ subsets of $\mathcal {A}_k$ that are feasible for different histories, and $d_k({\boldsymbol h}_k)$ is a composite of subset-specific rules; an example is given below.

**TABLE 1:** Summary of notation.

Symbol	Definition
**Treatment regime**	
$\mathcal {A}_k$	Finite set of treatment options at Decision $k$, $k=1,\ldots ,K$
$\tau _1$	Baseline, $\tau _1=0$
${\boldsymbol x}_1$	Covariate information available on individual at baseline; e.g., demographics, medical history variables, clinical and physiologic measures, etc.
$\tau _k$	Time at which Decision $k$ is reached (defined as long as the event has not yet occurred), $k=2,\ldots ,K$
${\boldsymbol x}_k$	Covariate information obtained between Decisions $k-1$ and $k$, e.g., updated clinical and physiologic measures, timing and severity of adverse reactions to and response to prior treatments, etc. (defined as long as the event has not yet occurred), $k=2,\ldots ,K$
$a_k$	Treatment option in $\mathcal {A}_k$ given at Decision $k$, $k=1,\ldots ,K$ (defined as long as the event has not yet occurred)
$\overline{a}_k$	$\overline{a}_k = (a_1,\ldots ,a_k)$ , $k=1,\ldots ,K$ as long as $a_k$ is defined; $\overline{\tau }_k$, $\overline{{\boldsymbol x}}_k$ defined analogously
${\boldsymbol h}_k$	History available on an individual at Decision $k$:
	${\boldsymbol h}_1 = (\tau _1,{\boldsymbol x}_1)$
	${\boldsymbol h}_k = (\overline{\tau }_k,\overline{{\boldsymbol x}}_k,\overline{a}_{k-1})$ if individual has not experienced the event by Decision $k$, $k = 2,\ldots ,K$
	$= (\overline{\tau }_{j-1},\overline{{\boldsymbol x}}_{j-1},\overline{a}_{j-1},t)$ if individual experiences the event at time $t$ between Decisions $j-1$ and $j$,
	$j \le k$ , $k = 2,\ldots ,K$
$\Psi _k({\boldsymbol h}_k) \subseteq \mathcal {A}_k$	Subset of treatment options in $\mathcal {A}_k$ that are feasible for an individual with history ${\boldsymbol h}_k$, $k=1,\ldots ,K$
$d$	Treatment regime $d = \lbrace d_1({\boldsymbol h}_1),\ldots , d_K({\boldsymbol h}_K)\rbrace$ with rules $d_k({\boldsymbol h}_k)$ taking values in $\Psi _k({\boldsymbol h}_k)$, $k=1,\ldots ,K$
**Potential outcomes**	
$T^{*}(d)$	Potential time to the event if an individual were to receive treatment by following the rules in regime $d$
$\lambda (u,d)$	Hazard rate of experiencing the event under regime $d$
$N^{*}(u,d)$	$N^{*}(u,d) = I\lbrace T^{*}(d) \le u\rbrace$ , the event-time counting process associated with $T^{*}(d)$
$Y^{*}(u,d)$	$Y^{*}(u,d) = I\lbrace T^{*}(d) \ge u\rbrace$ , at-risk process associated with $T^{*}(d)$
**Observed data**	
$\kappa$	observed number of decision points reached by a subject before experiencing the event or censoring
$\mathcal {T}_1$	Baseline, $\mathcal {T}_1=0$
${\boldsymbol X}_1$	Covariate information observed at baseline
$\mathcal {T}_k$	Observed time at which a subject reaches Decision $k$, $k=2,\ldots ,\kappa$
${\boldsymbol X}_k$	Covariate information observed on a subject between Decisions $k-1$ and $k$, $k=2,\ldots ,\kappa$
$A_k$	Treatment option in $\mathcal {A}_k$ received at Decision $k$, $k=1,\ldots ,\kappa$
$\overline{A}_k$	$\overline{A}_k= (A_1,\ldots ,A_k)$ , $k=1,\ldots ,\kappa$; $\overline{\mathcal {T}}_k$ and $\overline{{\boldsymbol X}}_k$ defined analogously
${\boldsymbol H}_k$	Observed history up to Decision $k$:
	${\boldsymbol H}_1 = (\mathcal {T}_1, {\boldsymbol X}_1)$
	${\boldsymbol H}_k = (\overline{\mathcal {T}}_k,\overline{{\boldsymbol X}}_k,\overline{A}_{k-1})$ if $\kappa \ge k$; $= (\overline{\mathcal {T}}_\kappa ,\overline{{\boldsymbol X}}_\kappa ,\overline{A}_\kappa ,U,\Delta )$ if $\kappa < k$

**FIGURE 2:**
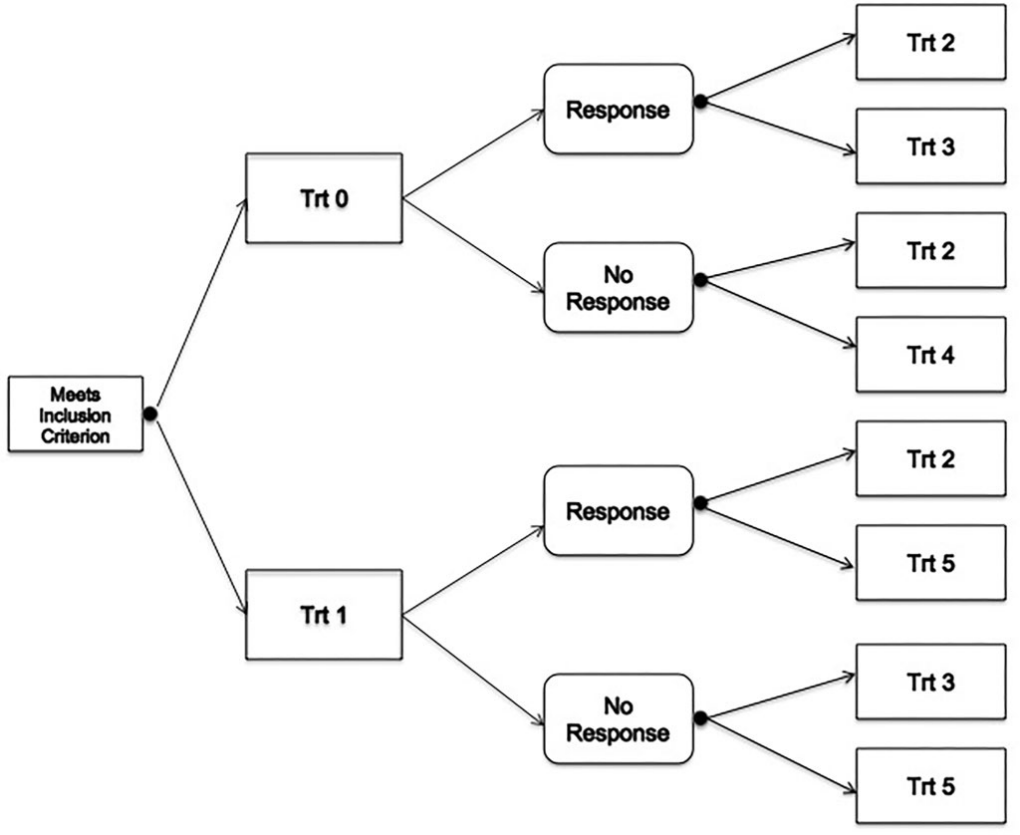
Schematic depicting the design of a 2-stage SMART with $\mathcal {A}_1=\lbrace 0,1\rbrace$, $\mathcal {A}_2 = \lbrace 2,3, 4,5\rbrace$ in which subjects who do not experience the event prior to Decision 2 are classified as responders or nonresponders to Decision 1 treatment and are randomized to stage 2 treatments within feasible subsets of $\mathcal {A}_2$ depending on their stage 1 treatment and response status; here, $\ell _2=4$. Solid circles represent points of randomization. The 8 embedded regimes implied by this design are of the form “Give Trt $a$ initially; if the event does not occur before Decision 2 and response, give Trt $b$, otherwise if the event does not occur before Decision 2 and no response, give Trt $c$,” where the 8 regimes correspond to $(a,b,c) = (0,2,2), (0,2,4), (0,3,2), (0,3,4), (1,2,3), (1,2,5), (1,5,3) (1,5,5)$.

In a SMART, the feasible sets and regimes that can be studied are dictated by the design. For example, in the SMART in Figure 2, with $K=2$, $\Psi _1({\boldsymbol h}_1) = \mathcal {A}_1 = \lbrace 0, 1\rbrace$ for any ${\boldsymbol h}_1$, and regimes that can be studied have rules $d_1({\boldsymbol h}_1)$ that return one of the treatments in $\mathcal {A}_1$. At Decision 2, $\Psi _2({\boldsymbol h}_2) = \emptyset$ for subjects who experience the event prior to Decision 2. Otherwise, $\Psi _2({\boldsymbol h}_2)$ is a subset of $\mathcal {A}_2= \lbrace 2, 3, 4, 5\rbrace$ determined by the stage 1 treatment and response status contained in histories ${\boldsymbol h}_2$ for which the event has not yet taken place; there are $\ell _2 = 4$ such subsets, and regimes that can be studied have rules $d_2({\boldsymbol h}_2)$ that are a composite of subset-specific rules. For example, if ${\boldsymbol h}_2$ indicates that $a_1=1$ and the individual is a responder, a subset-specific rule selects Decision 2 treatment from $\Psi _2({\boldsymbol h}_2) = \lbrace 2, 5\rbrace$; if ${\boldsymbol h}_2$ instead indicates $a_1=1$ and nonresponse, a subset-specific rule selects from $\Psi _2({\boldsymbol h}_2) = \lbrace 3, 5\rbrace$. The SMART in Figure 2 has 8 embedded regimes satisfying these conditions.

### 2.2 Problem formulation

We can now formalize the null hypothesis of interest, which, as is standard, is in terms of potential outcomes. We follow Tsiatis et al. (2020, Section 8.3.2), who define for any sequence of treatment options $\overline{a}_K \in \mathcal {A}_1 \times \cdots \times \mathcal {A}_K$ that could hypothetically be given to an individual the potential number of decision points that would be reached and potential times at which they would be reached, potential covariate information that would arise, and the associated potential event time. As in (8.50) of Tsiatis et al. (2020), denote by $\mathcal {W}^{*}$ the collection of these potential variables for all possible sequences. For any regime $d$ and associated feasible sets, let $T^{*}(d)$ denote the potential time to the event if a randomly chosen individual in the population were to receive treatments by following the rules in $d$; as in Tsiatis et al. (2020, Section 8.3.2), $T^{*}(d)$ can be defined in terms of $\mathcal {W}^{*}$. Then, we can denote the hazard rate of experiencing the event at time $u$ for an individual following the rules in $d$ by $\lambda (u,d) = \lim _{du \rightarrow 0} (du)^{-1} P\lbrace u \le T^{*}(d) < u+du| T^{*}(d) \ge u\rbrace$, $u \ge 0$. Thus, given a set $\mathcal {D}= \lbrace d^1,\ldots ,d^D\rbrace$ of $D$ regimes of interest, e.g., the embedded regimes in a SMART or a subset thereof, we are interested in testing the null hypothesis


(1) \begin{eqnarray*}
H_0\mathord {:} \, \, \, \lambda (u,d^1) = \cdots = \lambda (u,d^D), \, \, \, u \ge 0,
\end{eqnarray*}

versus the alternative hypothesis that at least 1 hazard rate differs from the rest.

Analogous to the formulation of the standard logrank test in a conventional (single-stage) multi-arm clinical trial, to motivate our proposed test of $H_0$ in (1), for the regimes $d^j$, $j=1,\ldots ,D$, of interest, assume a proportional hazards relationship; namely,


(2) \begin{eqnarray*}
\lambda (u,d^j) = \lambda _0(u) \exp (\beta _j), \, \, j=1,\ldots ,D-1.
\end{eqnarray*}

Under (2), the baseline hazard $\lambda _0(u)$ is $\lambda (u,d^D)$, and (1) corresponds to $H_0\mathord {:}\, \, \beta _1 = \cdots = \beta _{D-1} = 0.$ As with the standard logrank test, we do not necessarily believe that (2) holds. In fact, (2) generally cannot hold; e.g., intuitively, the hazards for 2 “shared path” regimes cannot be proportional under departures from $H_0$. Rather, (2) is solely a convenient mechanism by which to motivate a test of (1).

Toward that goal, we consider the possibly misspecified model (2) and develop a test statistic in the spirit of generalized score tests (Boos, 1992). For any regime $d$, let $N^{*}(u,d) = I\lbrace T^{*}(d) \le u\rbrace$ and $Y^{*}(u,d) = I\lbrace T^{*}(d) \ge u\rbrace$ be the event-time counting process and at-risk process associated with $T^{*}(d)$. Assuming (2) holds and letting $\Lambda _0(u) = \int _0^u \lambda _0(s)\, ds$ and $\Lambda (u,d) =\int _0^u \lambda (s,d)\, ds$ denote the cumulative hazard functions associated with $\lambda _0(u)$ and $\lambda (u,d)$, $E\lbrace dN^{*}(u,d) - d\Lambda (u,d) Y^{*}(u,d)\rbrace = 0$. Then, for the set $\mathcal {D}$ of regimes of interest,


\begin{eqnarray*}
E\lbrace dN^{*}(u,d^D) &-& d\Lambda _0(u) Y^{*}(u,d^D)\rbrace = 0, \\
E\lbrace dN^{*}(u,d^j) &-& d\Lambda _0(u) \exp (\beta _j) Y^{*}(u,d^j)\rbrace = 0,\\
&& j=1,\ldots ,D-1,
\end{eqnarray*}

which defines a dynamic regime marginal structural model (Orellana et al., 2010). If we were able to observe $T^{*}(d^j)$ for all $j=1,\ldots ,D$, and if (2) were correct, then, analogous to Yang et al. (2018), for each regime $j=1,\ldots ,D$, we would be led to a set of estimating functions for the unknown quantities $\Lambda _0(u)$, $u \ge 0$, and $\beta _1,\ldots ,\beta _{D-1}$. That associated with $\Lambda _0(u)$, $u \ge 0$, is the infinite-dimensional expression


\begin{eqnarray*}
dN^{*}(u,d^j) &-& d\Lambda _0(u) \exp \Big \lbrace \sum _{j^{\prime }=1}^{D-1} \beta _{j^{\prime }} I(j^{\prime }=j)\Big \rbrace Y^{*}(u,d^j), \,\\
&& u \ge 0,
\end{eqnarray*}

and that associated with $\beta _{j^{\prime }}$, $j^{\prime }=1,\ldots ,D-1$, is


\begin{eqnarray*}
&&\int ^\infty _0 \, I(j^{\prime }=j) \Bigg [dN^{*}(u,d^j)\\
&&\qquad- d\Lambda _0(u) \exp \Bigg \lbrace \sum _{j^{\prime }=1}^{D-1} \beta _{j^{\prime }} I(j^{\prime }=j)\Bigg \rbrace Y^{*}(u,d^j)\Bigg ].
\end{eqnarray*}

Note that this expression is equal to 0 except when $j^{\prime }=j$. Following Orellana et al. (2010) and Yang et al. (2018), we can combine these estimating functions across the $D$ regimes in $\mathcal {D}$ via a weighted sum, yielding the estimating functions


(3) \begin{eqnarray*}
&&\sum ^D_{j=1} w(u, d^j) \Bigg [dN^{*}(u,d^j)- d\Lambda _0(u)\\
&&\quad \times \exp \Bigg \lbrace \sum _{j^{\prime }=1}^{D-1} \beta _{j^{\prime }}I(j^{\prime }=j)\Bigg \rbrace Y^{*}(u,d^j)\Bigg ], \, \, u \ge 0,
\end{eqnarray*}

(4) \begin{eqnarray*}
&&\int ^\infty _0 \, w(u, d^j) \lbrace dN^{*}(u,d^j) - d\Lambda _0(u) \exp (\beta _j) Y^{*}(u,d^j)\rbrace ,\\
&&\quad j=1,\ldots ,D-1,
\end{eqnarray*}

where, for any regime $d$, $w(u,d)$ is a possibly time-dependent weight function discussed later.

Now, under $H_0\mathord {:}\, \, \beta _1 = \cdots = \beta _{D-1} = 0$, if it were possible to observe $T^{*}(d^j)$, $j=1,\ldots ,D$, for each of $n$ subjects in a SMART (or observational study), indexed by $i$, from (3), for any $u \ge 0$, the “score equation” associated with the “nuisance parameter” $\Lambda _0(u)$ is


(5) \begin{eqnarray*}
\sum ^n_{i=1}\sum ^D_{j=1} w(u, d^j)\lbrace dN^{*}_i(u,d^j) -d\Lambda _0(u)Y^{*}_i(u,d^j)\rbrace =0,\\
\end{eqnarray*}

yielding


\begin{eqnarray*}
d\widehat{\Lambda }_0(u) = \frac{\sum ^n_{i=1}\sum ^{D}_{j=1}w(u,d^j) dN^{*}_i(u,d^j)}{\sum ^n_{i=1}\sum ^{D}_{j=1}w(u,d^j) Y^{*}_i(u,d^j)}.
\end{eqnarray*}

From (4), the “score equation” associated with $\beta _j$ is


\begin{eqnarray*}
\sum ^n_{i=1}\int ^\infty _0\, w(u,d^j) \lbrace dN^{*}_i(u,d^j) - d\Lambda _0(u)Y^{*}_i(u,d^j) \rbrace;
\end{eqnarray*}

substituting $d\widehat{\Lambda }_0(u)$ yields the “score vector” $\boldsymbol{\mathfrak {T}}^{*}=(\mathfrak {T}^{*\, 1},\ldots , \mathfrak {T}^{*\, D-1})^T$ associated with $\beta _1,\ldots ,\beta _{D-1}$, where


(6) \begin{eqnarray*}
\mathfrak {T}^{*\, j} = \sum ^n_{i=1}\int ^\infty _0\, w(u,d^j) \lbrace dN^{*}_i(u,d^j) - d\widehat{\Lambda }_0(u)Y^{*}_i(u,d^j) \rbrace .\\
\end{eqnarray*}

These developments suggest that a test statistic for $H_0$ could be constructed as the quadratic form $\mathbb {Z}^{*} = \boldsymbol{\mathfrak {T}}^{*\, T} {\boldsymbol \Sigma }^{* \, -1} \boldsymbol{\mathfrak {T}}^{*}$ for suitable covariance matrix ${\boldsymbol \Sigma }^{*}$. Of course, for a given set of regimes $\mathcal {D}$, we cannot observe $T^{*}(d^j)$, $j=1,\ldots ,D$, for each subject. Thus, to exploit this formulation, we must relate (5)–(6) to the data actually available, discussed next.

### 2.3 Data and assumptions

As in Study C9710, the event time for some subjects may be censored. Thus, following Tsiatis et al. (2020, Section 8.3.2), the observed data on a participant in a $K$-stage SMART (or observational study) can be represented as


(7) \begin{eqnarray*}
\mathcal {O}= \lbrace \kappa , \mathcal {T}_1, {\boldsymbol X}_1, A_1, \mathcal {T}_2, {\boldsymbol X}_2, A_2, \ldots , \mathcal {T}_\kappa , {\boldsymbol X}_\kappa , A_\kappa , U, \Delta ),\\
\end{eqnarray*}

where $\kappa$ is the observed number of decision points reached by the subject before experiencing the event or censoring, $1 \le \kappa \le K$; $U$ is the observed time to the event or censoring, whichever first, and $\Delta =1$ or 0 as $U$ is the event or censoring time; and $(\mathcal {T}_k,{\boldsymbol X}_k,A_k)$ and the observed history ${\boldsymbol H}_k$ up to Decision $k=1,\ldots ,K$, are defined in Table 1. Let $H(u)$ be the history available up to time $u \ge 0$, which comprises all covariate information, treatments, and times of decision points up to and including $u$; for $k \le \kappa$, if $u=\mathcal {T}_k$, $H(u) = {\boldsymbol H}_k$, and if $\mathcal {T}_{k-1} < u < \mathcal {T}_k$, $H(u)$ comprises ${\boldsymbol H}_{k-1}$ and any components of ${\boldsymbol X}_k$ collected in that interval.

Relating (5) and (6) to the observed data (7) is possible under standard identifiability assumptions, which are discussed in detail in Tsiatis et al. (2020, Section 8.3.2). The consistency assumption in these authors’ (8.66) states that the observed data are equal to their potential analogs under the treatments actually received. The sequential randomization assumption states that $\mathcal {W}^{*} {\perp\!\!\!\perp} A_k| ({\boldsymbol H}_k, \kappa \ge k)$, $k=1,\ldots ,K$, where “${\perp\!\!\!\perp}$” denotes statistical independence, which is guaranteed by randomization in a SMART but must be made based on domain considerations in an observational study. We assume that censoring is noninformative in the sense that the cause-specific hazard for censoring satisfies $\lambda _c\lbrace u| H(u), \mathcal {W}^{*}\rbrace = \lim _{du \rightarrow 0} (du)^{-1} P\lbrace u \le U < u+du,\Delta =0| U\ge u, H(u), \mathcal {W}^{*}\rbrace = \lim _{du \rightarrow 0} (du)^{-1} P\lbrace u \le U < u+du,\Delta =0| U\ge u, H(u)\rbrace = \lambda _c\lbrace u| H(u)\rbrace$, say, so that censoring depends only on information observed through time $u$. The positivity assumption states roughly that $\omega _k({\boldsymbol h}_k,a_k) = P(A_k=a_k|{\boldsymbol H}_k={\boldsymbol h}_k, \kappa \ge k) > 0$ for all ${\boldsymbol h}_k$ such that $P({\boldsymbol H}_k={\boldsymbol h}_k, \kappa \ge k) > 0$ and $a_k \in \Psi _k({\boldsymbol h}_k)$, $k=1,\ldots ,K$, which is true by design in a SMART; see Tsiatis et al. (2020, Section 8.3.2) for a precise formulation.

### 2.4 Test procedure

As in Section 2.2, we wish to define a test statistic $\mathbb {Z}$ analogous to $\mathbb {Z}^{*}$, where $\mathbb {Z}$ is a function of the observed data (7). To this end, under the above assumptions, we first obtain analogs to (5) and (6) in terms of the observed data based on inverse probability weighting, where the analog to $\mathfrak {T}^{*\, j}$ given in (6) is the $j$th element of a “score vector” $\boldsymbol{\mathfrak {T}}$, say, analogous to $\boldsymbol{\mathfrak {T}}^{*}$. We then derive a suitable covariance matrix ${\boldsymbol \Sigma }$ analogous to ${\boldsymbol \Sigma }^{*}$.

For regime $d$ and $u \ge 0$, define


(8) \begin{eqnarray*}
C(u,d) &=& \prod ^\kappa _{k=1} \left[I\lbrace \mathcal {T}_k> u\rbrace + I\lbrace \mathcal {T}_k \le u\rbrace I\lbrace A_k = d_k({\boldsymbol H}_k)\rbrace \right],\\
&& u \ge 0,
\end{eqnarray*}

the indicator of whether or not the treatments received by an individual through time $u$ are consistent with having followed the rules in $d$ through $u$. Similarly, define


(9) \begin{eqnarray*}
\pi (u,d) &=& \prod ^\kappa _{k=1} \left[I\lbrace \mathcal {T}_k> u\rbrace + I\lbrace \mathcal {T}_k \le u\rbrace \omega _k\lbrace {\boldsymbol H}_k,d_k({\boldsymbol H}_k) \rbrace \right],\\
&& u \ge 0,
\end{eqnarray*}

which can be viewed roughly as the probability of receiving treatments consistent with $d$ through all decisions reached by an individual by time $u$. In Web Appendix A of the SupplementaryMaterial, we give the form of $C(u,d)$ and $\pi (u,d)$ in the SMART in Figure 2.

Denote the survival function for censoring under the noninformative censoring assumption as $K_c\lbrace u| H(u)\rbrace = \exp [-\int ^u_0 \lambda _c\lbrace s|H(s)\rbrace \, ds]$, and define $N(u) = I(U \le u, \Delta =1)$ and $Y(u) = I(U \ge u)$. Then, under the above assumptions, we argue in Web Appendix A that an observed data analog to (5) is given by


(10) \begin{eqnarray*}
&&\sum ^n_{i=1}\sum ^D_{j=1} \Omega _i(u,d^j)\lbrace dN_i(u) -d\Lambda _0(u) Y_i(u)\rbrace =0,\\
&&\qquad\Omega (u,d) = \frac{C(u,d) I(U \ge u) }{\pi (u,d) K_c\lbrace u| H(u)\rbrace } w(u, d),
\end{eqnarray*}

so that


\begin{eqnarray*}
d\widehat{\Lambda }_0(u) = \frac{\sum ^n_{i=1}\sum ^{D}_{j=1}\Omega _i(u,d^j) dN_i(u)}{\sum ^n_{i=1}\sum ^{D}_{j=1}\Omega _i(u,d^j) Y_i(u)}.
\end{eqnarray*}

Similarly, under $H_0$, an analog to the “score equation” for $\beta _j$ is $\sum ^n_{i=1}\int ^\infty _0 \, \Omega _i(u, d^j) \lbrace dN_i(u) - d\Lambda _0(u) Y_i(u)\rbrace$, $j= 1,\ldots ,D-1$, so that, substituting $d\widehat{\Lambda }_0(u)$, the observed data analog to (6) is


(11) \begin{eqnarray*}
\mathfrak {T}^j &=& \sum ^n_{i=1}\int ^\infty _0\, \Omega _i(u,d^j) \lbrace dN_i(u) - d\widehat{\Lambda }_0(u)Y_i(u) \rbrace ,\\
&&\quad j=1,\ldots ,D-1.
\end{eqnarray*}

Thus, the “score vector” analogous to $\boldsymbol{\mathfrak {T}}^{*}$ is given by $\boldsymbol{\mathfrak {T}}= (\mathfrak {T}^1,\ldots ,\mathfrak {T}^{D-1})^T$. In (11), $\Omega _i(u,d)$ defined in (10) incorporates inverse probability weighting by $\pi (u,d)$ in (9) and the probability of being uncensored at time $u$. Note that, in a SMART, the treatment assignment probabilities $\omega _k({\boldsymbol h}_k,a_k)$, $k=1,\ldots ,K$, involved in (9) are the known randomization probabilities used in the design at each stage; however, the formulation of (11) is valid if the data (7) arise from an observational study, in which case $\omega _k({\boldsymbol h}_k,a_k)$ are likely unknown; see Section 2.5.

The censoring survival function $K_c\lbrace u| H(u)\rbrace$ in $\Omega (u,d)$ is a function of the history $H(u)$. Thus, in principle, censoring can depend on covariates and treatments received observed up to time $u$. As in a well-conducted conventional, single-stage clinical trial and in Tsiatis et al. (2020, Section 8.3.3), censoring in a SMART may be primarily administrative, in which case it is reasonable to assume that, in addition to being noninformative in the sense defined above, censoring is independent of $H(u)$, and write $K_c(u)$. This assumption is analogous to that underlying the standard logrank test for a conventional multi-arm trial, namely, that censoring is noninformative and independent of baseline covariates and treatment assignment. Under this condition, we can take $w(u,d^j) = K_c(u)$, $j=1,\ldots ,D$, so that


(12) \begin{eqnarray*}
\Omega (u,d^j) = \frac{C(u,d^j) I(U \ge u) }{\pi (u,d^j) },
\end{eqnarray*}

so that (11) and thus $\boldsymbol{\mathfrak {T}}$ does not depend on the censoring distribution. In fact, analogous to the usual logrank test, (12) holds if censoring depends only on stage 1 (baseline) treatment assignment, which we write as $K_c(u|a_1)$, and we take $w(u,d^j) = K_c(u| a^j)$, where $a^j$ is the stage 1 treatment assigned by regime $d^j$. We assume henceforth that censoring is independent of $H(u)$ with the possible exception of stage 1 treatment and that $w(u,d)$ is specified so that (12) holds; more complex dependence on $H(u)$ is discussed in Web Appendix B.

A suitable choice for ${\boldsymbol \Sigma }$ is the (large-sample) approximation to the covariance matrix of $\boldsymbol{\mathfrak {T}}$ under $H_0$. However, a challenge to deriving ${\boldsymbol \Sigma }$ is that $\mathfrak {T}^j$ in (11) for each $j = 1,\ldots ,D-1$, and thus $\boldsymbol{\mathfrak {T}}$, is not a sum of independent and identically distributed (iid) mean-zero terms, so that standard asymptotic theory does not apply. Defining $d\overline{N}_i(u) = \sum ^{D}_{j=1}\Omega _i(u,d^j) dN_i(u)$, $\overline{Y}_i(u) = \sum ^{D}_{j=1}\Omega _i(u,d^j) Y_i(u)$, and $\widehat{q}(u,d^j) = \big \lbrace \sum ^n_{i=1}\Omega _i(u,d^j) Y_i(u)\big \rbrace /\sum ^n_{i=1}\overline{Y}_i(u)$, we show in Web Appendix C that $n^{-1/2}$ times (11) can be written as


(13) \begin{eqnarray*}
n^{-1/2} \mathfrak {T}^j &=& n^{-1/2} \sum ^n_{i=1}\mathfrak {T}^j_i + o_p(1),\\
\mathfrak {T}^j_i &=& \Bigg[\vphantom{\sum ^D_{j^{\prime }=1}} \int ^\infty _0 \Omega _i(u,d^j) \lbrace dN_i(u) - d\Lambda _0(u)Y_i(u) \rbrace\\
&&- \sum ^D_{j^{\prime }=1} \int ^\infty _0 \Omega _i(u,d^{j^{\prime }}) q(u,d^j) \lbrace dN_i(u)\\
&&- d\Lambda _0(u)Y_i(u) \rbrace \Bigg],
\end{eqnarray*}

where $q(u,d)$ is the limit in probability of $\widehat{q}(u,d)$, so that $n^{-1/2} \boldsymbol{\mathfrak {T}}= n^{-1/2} \sum ^n_{i=1}\boldsymbol{\mathfrak {T}}_i + o_p(1)$, $\boldsymbol{\mathfrak {T}}_i = (\mathfrak {T}^1_i,\ldots ,\mathfrak {T}^{D-1}_i)^T$, $i=1,\ldots ,n$, and thus $n^{-1/2} \boldsymbol{\mathfrak {T}}$ is asymptotically equivalent to $n^{-1/2}$ times a sum of mean-zero iid terms. Thus, the asymptotic covariance matrix of $n^{-1/2} \boldsymbol{\mathfrak {T}}$ can be approximated by ${\boldsymbol \Sigma }= n^{-1} \sum ^n_{i=1}(\boldsymbol{\mathfrak {T}}_i \boldsymbol{\mathfrak {T}}_i^T)$. Because $q(u,d^j)$ and $d\Lambda _0(u)$ are unknown, substitute $\widehat{q}(u,d^j)$ for $q(u,d^j)$ and $d\widehat{\Lambda }_0(u)$ for $d\Lambda _0(u)$ in (13), and denote the resulting approximation to $\mathfrak {T}^j_i$ as $\widehat{\mathfrak {T}}^j_i$. Then, letting $\widehat{\boldsymbol{\mathfrak {T}}}_i = (\widehat{\mathfrak {T}}^1_i,\ldots , \widehat{\mathfrak {T}}^{D-1}_i)^T$, ${\boldsymbol \Sigma }$ can be estimated by $\widehat{{\boldsymbol \Sigma }}= n^{-1} \sum ^n_{i=1}(\widehat{\boldsymbol{\mathfrak {T}}}_i \widehat{\boldsymbol{\mathfrak {T}}}_i^T)$.

**FIGURE 3:**
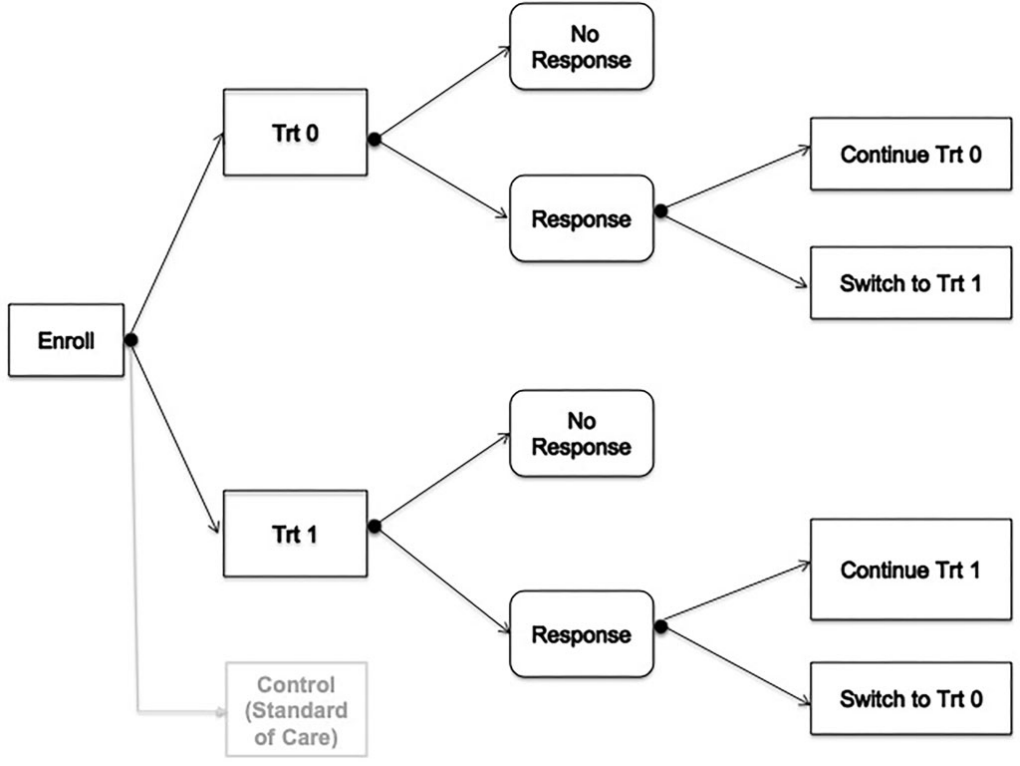
Schematic depicting the design of 2 possible 2-stage SMARTs, without and with a control (“standard of care”) regime to which subjects can be randomized up front. In the former design, with no control regime, $\mathcal {A}_1=\lbrace 0,1\rbrace$; in the latter, $\mathcal {A}_1 = \lbrace 0, 1,$control}. In either design, subjects who receive stage 1 options Trt 0 or 1 and do not experience the event prior to Decision 2 are classified as nonresponders or responders to Decision 1 treatment, nonresponders continue stage 1 treatment, and responders are randomized to either continue stage 1 treatment or switch; thus, for such subjects $\mathcal {A}_2 = \lbrace 0, 1\rbrace$, where 0 indicates continue and 1 indicates switch. Solid circles represent points of randomization. The 4 embedded regimes excluding the possible control regime are of the form “Give Trt $a$ initially; if the event does not occur before response status is ascertained and nonresponse, continue; else if response, give $b$,” where the 4 regimes correspond to $(a,b) = (0,0), (0,1), (1,0), (1, 1)$.

We thus propose for practical use the test statistic $\mathbb {Z}= n^{-1} \boldsymbol{\mathfrak {T}}^T \widehat{{\boldsymbol \Sigma }}^{-} \boldsymbol{\mathfrak {T}}$, where $\widehat{{\boldsymbol \Sigma }}^{-}$ is a generalized inverse of $\widehat{{\boldsymbol \Sigma }}$. As in Wu et al. (2023), we use a generalized inverse, as ${\boldsymbol \Sigma }$ and thus $\widehat{{\boldsymbol \Sigma }}$ may be singular due to a dependency structure in ${\boldsymbol \Sigma }$ induced by some SMART designs and the set of regimes of interest. For example, for the SMART in Figure 2, if $\mathcal {D}$ is the set of all $D=8$ embedded regimes, the hazards for the 4 regimes starting with the same stage 1 treatment involve a linear dependency, so that ${\boldsymbol \Sigma }(7 \times 7)$ has rank 5. In the SMART in Figure 3, ignoring the control, there is no such dependency, so if $\mathcal {D}$ is the set of $D=4$ embedded regimes, ${\boldsymbol \Sigma }$ is of full rank (= 3). In general, under $H_0$, $\mathbb {Z}$ has an approximate $\chi ^2_\nu$ distribution with degrees of freedom $\nu = \mathrm{rank}({\boldsymbol \Sigma })$.

In (11) and (13), the implied range of integration is 0 to at most the largest observed event time. Often, the number of subjects at risk at event times leading up to this time may be small, and even fewer of these may have treatment experience consistent with a particular regime. As a result, small numbers of subjects may have undue influence on the test statistic. Thus, analogous to Kidwell and Wahed (2013), we recommend truncating the range of integration at a time $L \le$ the largest observed event time. As a rule of thumb, we suggest choosing $L$ so that 1% to 4% of subjects remain at risk at $L$.

### 2.5 Improving efficiency

In a SMART, the randomization probabilities $\omega _k({\boldsymbol h}_k,a_k)$, $k=1,\ldots ,K$, are known, and they are treated as known in (12) in the foregoing developments. However, although counterintuitive, it is well known (eg, Tsiatis, 2006) that estimating these probabilities can lead to more efficient inferences. Accordingly, we propose fitting parametric models $\omega _k({\boldsymbol h}_k,a_k; {\boldsymbol \gamma }_k)$, e.g., logistic models fitted by maximum likelihood (ML), yielding ML estimators $\widehat{{\boldsymbol \gamma }}_k$. In a SMART, these models are chosen so that the ML estimators for the randomization probabilities are the sample proportions of subjects randomized to each feasible treatment given their histories. For example, in the SMART in Figure 2, the estimator for the probability of being assigned Trt 2 for responders for whom $a_1=$ Trt 0 is the sample proportion subjects who received Trt 0, reached stage 2 as responders, and were assigned to Trt 2. In an observational study, $\omega _k({\boldsymbol h}_k,a_k)$ are unknown and must be modeled; we discuss modeling and fitting of $\omega _k({\boldsymbol h}_k,a_k)$ in both SMARTs and observational studies in Web Appendix B.

In Web Appendix D, following Tsiatis (2006), we present an argument demonstrating that, if the randomization probabilities in a SMART are modeled parametrically and estimated by ML as above and the fitted models $\omega _k({\boldsymbol h}_k,a_k; \widehat{{\boldsymbol \gamma }}_k)$, $k=1,\ldots ,K$, are substituted in place of the known probabilities, as long as estimation of ${\boldsymbol \gamma }= ({\boldsymbol \gamma }_1^T,\ldots ,{\boldsymbol \gamma }_K^T)^T$ is taken into appropriate account in a modified test statistic, the resulting test of $H_0$ will be more powerful than that based on $\mathbb {Z}$. In an observational study, if the $\omega _k({\boldsymbol h}_k,a_k)$ are modeled parametrically as in Li et al. (2014), the argument also demonstrates how estimation of ${\boldsymbol \gamma }$ should be taken into account to lead to a test that is valid as long as the models are correctly specified.

The form of the modified test statistic follows from Web Appendix D and is given as follows. Let $\boldsymbol{\mathfrak {T}}(\widehat{{\boldsymbol \gamma }})$ be $\boldsymbol{\mathfrak {T}}$ with the models $\omega _k({\boldsymbol h}_k,a_k; \widehat{{\boldsymbol \gamma }}_k)$, $k=1,\ldots ,K$, fitted by ML substituted, and let ${\boldsymbol \Sigma }_\gamma$ be the asymptotic covariance matrix of $\boldsymbol{\mathfrak {T}}(\widehat{{\boldsymbol \gamma }})$. Let ${\boldsymbol S}_\gamma ({\boldsymbol \gamma })$ be the score vector associated with ${\boldsymbol \gamma }$; i.e., the vector of partial derivatives with respect to ${\boldsymbol \gamma }$ of the loglikelihood associated with ${\boldsymbol \gamma }$ for the posited models. See Web Appendix D for examples of the form of ${\boldsymbol S}_\gamma ({\boldsymbol \gamma })$. For $j=1,\ldots ,D-1$, let $\widehat{\mathfrak {T}}^j_i(\widehat{{\boldsymbol \gamma }})$, $i=1,\ldots ,n$, denote $\widehat{\mathfrak {T}}^j_i$ as defined above with the fitted models $\omega _k({\boldsymbol h}_k,a_k; \widehat{{\boldsymbol \gamma }}_k)$, $k=1,\ldots ,K$, substituted. For each $j=1,\ldots ,D-1$, carry out a linear regression of the $\widehat{\mathfrak {T}}^j_i(\widehat{{\boldsymbol \gamma }})$ on ${\boldsymbol S}_{\gamma ,i}(\widehat{{\boldsymbol \gamma }})$ and form for subjects $i = 1,\ldots ,n$ the residuals from this fit, which we denote as $\widehat{\mathfrak {T}}^{j,R}_i(\widehat{{\boldsymbol \gamma }})$, $i=1,\ldots ,n$. Then, we show in Web Appendix D that, letting $\widehat{\boldsymbol{\mathfrak {T}}}^R_i(\widehat{{\boldsymbol \gamma }}) = \lbrace \widehat{\mathfrak {T}}^{1,R}_i(\widehat{{\boldsymbol \gamma }}), \ldots , \widehat{\mathfrak {T}}^{D-1,R}_i(\widehat{{\boldsymbol \gamma }})\rbrace ^T$, the estimator for ${\boldsymbol \Sigma }_\gamma$ is given by $\widehat{{\boldsymbol \Sigma }}_\gamma = n^{-1} \sum ^n_{i=1}\lbrace \widehat{\boldsymbol{\mathfrak {T}}}^R_i(\widehat{{\boldsymbol \gamma }}) \widehat{\boldsymbol{\mathfrak {T}}}^R_i(\widehat{{\boldsymbol \gamma }}) ^T\rbrace$, and the modified test statistic is constructed as $\mathbb {Z}(\widehat{{\boldsymbol \gamma }}) = n^{-1}\widehat{\boldsymbol{\mathfrak {T}}}^R(\widehat{{\boldsymbol \gamma }})^T \widehat{{\boldsymbol \Sigma }}^{-}_\gamma \widehat{\boldsymbol{\mathfrak {T}}}^R(\widehat{{\boldsymbol \gamma }})$, where $\widehat{\boldsymbol{\mathfrak {T}}}^R(\widehat{{\boldsymbol \gamma }}) = \sum ^n_{i=1}\widehat{\boldsymbol{\mathfrak {T}}}^R_i(\widehat{{\boldsymbol \gamma }})$. We also present an argument that, asymptotically, the test of $H_0$ based on $\mathbb {Z}(\widehat{{\boldsymbol \gamma }})$ is at least as powerful as that based on $\mathbb {Z}$. As above, truncation of integration at some value $L$ should also be implemented.

In fact, as we discuss in Web Appendix D, if there are components of the history at any decision point that are associated with the outcome, it is possible to obtain an even more powerful test by exploiting these associations. The associated test statistic is obtained by regressing the $\widehat{\mathfrak {T}}^j_i(\widehat{{\boldsymbol \gamma }})$, $i=1,\ldots ,n$, not only on ${\boldsymbol S}_{\gamma ,i}(\widehat{{\boldsymbol \gamma }})$ but also on suitably chosen functions of the histories at each decision point to obtain the residuals $\widehat{\mathfrak {T}}^{j,R}_i(\widehat{{\boldsymbol \gamma }})$, $i=1,\ldots ,n$. Only components of the history thought to be strongly associated with the outcome should be incorporated, as including components that are only weakly associated could degrade performance if $n$ is not large. See Web Appendix D for a demonstration and further discussion.

As shown in the simulations in Section 3, when $n$ is not large, any of the tests based on $\mathbb {Z}$ or $\mathbb {Z}(\widehat{{\boldsymbol \gamma }})$ can be anti-conservative, i.e., reject $H_0$ too often, leading to inflated type I error. This behavior is because the estimator $\widehat{{\boldsymbol \Sigma }}$ or $\widehat{{\boldsymbol \Sigma }}_\gamma$, as appropriate, understates the uncertainty in the components of $\boldsymbol{\mathfrak {T}}$ or $\boldsymbol{\mathfrak {T}}(\widehat{{\boldsymbol \gamma }})$ when $n$ is not large, which is not uncommon with methods based on semiparametric theory. In Web Appendix E, we propose a small-$n$ bias correction to $\widehat{{\boldsymbol \Sigma }}$ and $\widehat{{\boldsymbol \Sigma }}_\gamma$ that, when the bias-corrected covariance matrix is used to form the test statistic, yields a test that more closely achieves the nominal level, as demonstrated in Section 3.

### 2.6 Comparison to existing methods

Our motivation for developing the proposed methods is to provide a unified framework for general $K$ allowing comparison of regimes in any arbitrary set of regimes within the class of “feasible regimes” given the available data (Tsiatis et al., 2020, Section 6.2.3). The data may be from a SMART or observational study; censoring mechanisms beyond independent censoring, as expected with observational data, are accommodated; and “covariate-adjustment” to improve power is possible. We focus on SMARTs and independent censoring in this article.

Kidwell and Wahed (2013) and Li et al. (2014) were the first to propose logrank-type tests for comparing regimes, including “shared path” regimes, with time-to-event outcome. Kidwell and Wahed (2013) restrict to a SMART with $K=2$ stages analogous to that in Figure 3 without the control regime and the 4 embedded regimes. Li et al. (2014) consider data from an observational study and in principle accommodate any $K$, although the method is studied only in a 2-stage observational setting analogous to the SMART considered by Kidwell and Wahed (2013) and regimes of the form, e.g., with $K=2$, “Give $a_1$; if response give $a_2$, else continue $a_1$.” Neither method supports incorporation of covariate information to improve power and both assume censoring is noninformative and independent of $H(u)$.

The “score vector” on which the test statistic of Li et al. (2014) is based is equivalent to our $\boldsymbol{\mathfrak {T}}$ with $w(u,d^j) = K_c(u)$, $j=1,\ldots ,D$, as in (12); because the Li et al. (2014) formulation does not include a weight function analogous to $w(u,d)$, it cannot be generalized to censoring mechanisms other than noninformative, independent censoring. Li et al. (2014) contend, contrary to the results in Section 2.5, that no account need be taken of estimation of ${\boldsymbol \gamma }$ in models $\omega _k({\boldsymbol h}_k,a_k; {\boldsymbol \gamma }_k)$, $k=1,\ldots ,K$. In Web Appendix D, we show that there is a misstatement in the proof that Li et al. (2014) present to justify this claim. Thus, their test as proposed is expected to be conservative, as seen in their simulations, where it rejects $H_0$ at a rate lower than the nominal level for even very large sample sizes.

The test statistic of Kidwell and Wahed (2013) is based on a vector of pairwise comparisons of the hazards corresponding to 3 of the embedded regimes against a fourth reference regime, and known randomization probabilities are used. Derivation of the covariance matrix of this vector is based on counting process martingale theory. However, as we discuss in Web Appendix D, the components of the vector need not be martingales with respect to the filtration given by the authors. Thus, the authors’ covariance formula may be biased for the true covariance matrix. As shown in the simulations in Section 3, depending on the data generative scenario, the test may be robust to this feature or may not achieve the nominal level of significance even for large sample sizes.

## 3 SIMULATION STUDIES

We present results of a suite of simulation studies, each with 5000 Monte Carlo data sets, under several data generative scenarios and SMART designs with $K=2$. For each scenario, we evaluate type I error under $H_0$ in (1) and power under alternatives to $H_0$ for level of significance 0.05 using the test statistic of Kidwell and Wahed (2013), $\mathbb {Z}_{\textrm {KW}}$, where applicable, and several versions of the proposed test statistic with estimated randomization probabilities, $\mathbb {Z}(\widehat{{\boldsymbol \gamma }})$, denoted as $\mathbb {Z}_{\textrm {U,nocov}}$ and $\mathbb {Z}_{\textrm {C,nocov}}$ without incorporation of covariates using the uncorrected and bias-corrected versions of the covariance matrix $\widehat{{\boldsymbol \Sigma }}_\gamma$, respectively; and as $\mathbb {Z}_{\textrm {U,cov}}$ and $\mathbb {Z}_{\textrm {C,cov}}$, which do incorporate covariates.

Scenarios 1 and 2 involve a SMART as in Figure 3 without the control regime, with 4 embedded regimes, and use the generative process of Kidwell and Wahed (2013), modified to include baseline and intermediate covariates $X_1$ and $X_2$ that may or may not be associated with the outcome. Details are in Web Appendix F; censoring ranges from 25% to 40%. First consider $H_0$. Scenario 1(a) involves no covariate associations, so duplicates the first null scenario of Kidwell and Wahed (2013); Scenario 1(b) is the same but with covariate associations. In Table 2, all tests are anti-conservative for $n=250$. Those based on $\mathbb {Z}_{\textrm {C,nocov}}$, $\mathbb {Z}_{\textrm {C,cov}}$, and $\mathbb {Z}_{\textrm {KW}}$ achieve the nominal level for $n = 500, 1000$; in 1(a); including what are actually unimportant covariates in $\mathbb {Z}_{\textrm {C,cov}}$ does not degrade performance. The tests based on $\mathbb {Z}_{\textrm {U,nocov}}$ and $\mathbb {Z}_{\textrm {U,cov}}$ are anti-conservative, supporting use of the bias correction. We also considered an alternative to $H_0$ under Scenario 1(b); see Web Appendix F. From Table 2, for tests that achieve the nominal level under $H_0$, incorporation of covariates associated with the event time using the proposed methods yields increases in power.

**TABLE 2:** Simulation results under the data generative scenarios described in the text under both the null hypothesis $H_0$ in (1) and alternatives based on 5000 Monte Carlo (MC) data sets, with all tests conducted with level of significance 0.05.

Scenario	$n$	$\zeta$	$\mathbb {Z}_{\textrm {U,nocov}}$	$\mathbb {Z}_{\textrm {C,nocov}}$	$\mathbb {Z}_{\textrm {U,cov}}$	$\mathbb {Z}_{\textrm {C,cov}}$	$\mathbb {Z}_{\textrm {KW}}$
	**Null scenarios**						
1(a)	250	–	0.076	0.063	0.074	0.062	0.056
	500	–	0.060	0.053	0.058	0.052	0.053
	1000	–	0.054	0.051	0.054	0.051	0.053
1(b)	250	–	0.071	0.061	0.073	0.059	0.059
	500	–	0.060	0.054	0.057	0.050	0.054
	1000	–	0.056	0.053	0.053	0.050	0.055
2(a)	250	–	0.075	0.060	0.081	0.066	0.081
	500	–	0.060	0.053	0.061	0.053	0.081
	1000	–	0.055	0.051	0.056	0.053	0.081
2(b)	250	–	0.071	0.060	0.074	0.063	0.070
	500	–	0.061	0.054	0.059	0.051	0.072
	1000	–	0.054	0.050	0.054	0.051	0.065
3(a)	250	0.00	0.061	0.051	0.057	0.049	0.052
	500	0.00	0.054	0.049	0.053	0.050	0.049
	1000	0.00	0.051	0.048	0.050	0.048	0.050
3(b)	250	0.00	0.057	0.050	0.060	0.050	0.042
	500	0.00	0.053	0.048	0.052	0.049	0.040
	1000	0.00	0.054	0.050	0.052	0.049	0.044
3(c)	250	0.00	0.063	0.051	0.064	0.053	0.074
	500	0.00	0.056	0.053	0.057	0.051	0.079
	1000	0.00	0.050	0.048	0.057	0.053	0.078
4	375	0.00	0.058	0.050	0.062	0.055	–
	750	0.00	0.055	0.052	0.056	0.052	–
	1500	0.00	0.050	0.049	0.051	0.049	–
5	500	0.00	0.055	0.048	0.055	0.049	–
	1000	0.00	0.056	0.051	0.054	0.050	–
	**Alternative scenarios**						
1(b)	500	–	0.534	0.487	0.573	0.558	0.468
	1000	–	0.813	0.807	0.891	0.887	0.784
2(b)	500	–	0.455	0.438	0.534	0.515	0.451
	1000	–	0.769	0.761	0.842	0.836	0.766
3(a)	250	3.50	0.726	0.706	0.827	0.807	0.720
	500	2.50	0.719	0.707	0.821	0.812	0.727
	1000	1.75	0.699	0.694	0.808	0.802	0.709
3(b)	250	2.25	0.731	0.711	0.813	0.794	0.709
	500	1.65	0.749	0.736	0.834	0.826	0.730
	1000	1.25	0.817	0.812	0.880	0.875	0.805
3(c)	250	7.00	0.687	0.656	0.808	0.784	0.559
	500	5.25	0.734	0.717	0.857	0.846	0.561
	1000	3.50	0.664	0.656	0.812	0.808	0.466
4	375	2.00	0.713	0.701	0.786	0.774	–
	750	1.50	0.774	0.767	0.852	0.848	–
	1500	1.00	0.728	0.724	0.823	0.818	–
5	500	4.00	0.742	0.729	0.874	0.862	–
	1000	3.00	0.695	0.687	0.847	0.842	–

Entries are MC proportions of times the test based on the indicated test statistic rejected $H_0$. $\mathbb {Z}_{\textrm {U,nocov}}$ and $\mathbb {Z}_{\textrm {C,nocov}}$ denote the proposed generalized logrank statistics $\mathbb {Z}(\widehat{{\boldsymbol \gamma }})$ based on estimated randomization probabilities but without incorporation of covariates using the uncorrected and corrected versions of the covariance matrix $\widehat{{\boldsymbol \Sigma }}_\gamma$, respectively; $\mathbb {Z}_{\textrm {U,cov}}$ and $\mathbb {Z}_{\textrm {C,cov}}$ denote the same but incorporating all covariates as described in Web Appendix D using the uncorrected and corrected versions of the covariance matrix $\widehat{{\boldsymbol \Sigma }}_\gamma$, respectively; and $\mathbb {Z}_{\textrm {KW}}$ denotes the test statistic proposed by Kidwell and Wahed (2013). Entries under $H_0$ have MC standard error of approximately 0.003; those under alternatives have MC standard error of about 0.006. In Scenarios 3–5, $\zeta$ dictates the alternative; see the text.

As discussed in Web Appendix F, the test of Kidwell and Wahed (2013) may be expected to be robust to departure from the martingale property under Scenario 1. Under $H_0$, Scenarios 2(a) and 2(b) are the same as 1(a) and 1(b) but with some parameter settings altered to create a stronger departure from the martingale property. From Table 2, under 2(a) and 2(b), all tests are anti-conservative with $n=250$; however, for $n=500, 1000$, the tests based on $\mathbb {Z}_{\textrm {C,nocov}}$ and $\mathbb {Z}_{\textrm {C,cov}}$ achieve the nominal level, while those based on $\mathbb {Z}_{\textrm {KW}}$ continue to be anti-conservative. In Web Appendix F, we speculate that this behavior may reflect the strong departure from the martingale property. Under an alternative to $H_0$ in 2(b), Table 2 shows gains in power when associated covariates are incorporated in the proposed methods.

Scenario 3 uses a different generative strategy and mimics the design of Study C9710; see Web Appendix F for details. All versions 3(a)–3(c) involve baseline and intermediate covariates ${\boldsymbol X}_1$ and $X_2$ that are associated with the time-to-event outcome. Censoring ranged from 30% to 45%. In Scenario 3(a), the martingale property holds approximately; from Table 2, under $H_0$, the tests based on $\mathbb {Z}_{\textrm {C,nocov}}$, $\mathbb {Z}_{\textrm {C,cov}}$, and $\mathbb {Z}_{\textrm {KW}}$ all achieve the nominal level, and incorporation of covariates yields increased power under alternatives to $H_0$. Scenarios 3(b) and 3(c) involve departures from the martingale property. While the tests based on $\mathbb {Z}_{\textrm {C,nocov}}$ and $\mathbb {Z}_{\textrm {C,cov}}$ achieve the nominal level under $H_0$ for all $n$, that based on $\mathbb {Z}_{\textrm {KW}}$ is conservative under 3(b) and anti-conservative under 3(c), which persists across all $n$.

Scenarios 4 and 5 involve data generative strategies similar to those for Scenario 3; details are in Web Appendix F. Scenario 4 includes an additional control arm as in Figure 3; Scenario 5 follows a design like that in Figure 2, with 8 embedded regimes. Thus, in both scenarios, the test of Kidwell and Wahed (2013) is not applicable. Under $H_0$, the proposed tests using the bias-corrected version of $\widehat{{\boldsymbol \Sigma }}_\gamma$ achieve the nominal level in all cases, and under alternatives to $H_0$, incorporation of covariates associated with the outcome leads to gains in power.

A secondary analysis in many SMARTs is comparison of specific pairs of embedded regimes, e.g., the most and least resource intensive or burdensome or each embedded regime against a control regime. In Web Appendix F, we report on simulation studies of the performance of the proposed tests and, where applicable, of tests based on the statistic of Kidwell and Wahed (2013), for comparing hazards for pairs of embedded regimes, including “shared path” regimes. An analysis of interest in some SMARTs is to compare more complex “feasible regimes” with rules depending on covariates; we report on a simulation study using the proposed methods for this purpose in Web Appendix F, as well as a scenario with $K=3$.

## 4 APPLICATION TO STUDY C9710

Web Appendix G provides details of the data, which involve $n=467$ subjects, of which 310 completed consolidation and were randomized to maintenance therapy and 157 experienced the event or were censored before completing consolidation. Baseline covariate ${\boldsymbol X}_1$ comprises 10 variables, and ${\boldsymbol X}_2$ collected between study entry and stage 2 includes 46 variables indicating adverse events. At Decision 1, let 0 (1) indicate ATRA (ATRA + arsenic trioxide) consolidation therapy; at Decision 2, 0 (1) indicates ATRA (ATRA+Mtx+MP) maintenance therapy. As in Figure 1, the study involves 4 embedded regimes of the form “Give consolidation therapy $a$; if subject completes consolidation before the event occurs, give maintenance therapy $b$,” denoted as Regimes 1, 2, 3, and 4 as $(a, b) = (0,0), (0,1), (1,0), (1,1)$. In Web Appendix G, we present estimates of the corresponding survival functions, which suggest that administering ATRA + arsenic trioxide initially, as in Regimes 3 and 4, is more beneficial than ATRA alone, consistent with the findings of Powell et al. (2010), and that Regime 4, which gives ATRA+Mtx+MP maintenance, may yield benefit over Regime 3.

Table 3 shows the results of tests of $H_0$ in (1) for different choices of the set $\mathcal {D}$. We tested $H_0$ several ways: using (i) the proposed test statistic $\mathbb {Z}(\widehat{{\boldsymbol \gamma }})$ and using the bias correction to $\widehat{{\boldsymbol \Sigma }}_\gamma$, without incorporation of covariates, denoted $\mathbb {Z}_{\textrm {C,nocov}}$ as in Table 2; (ii) same as (i) but incorporating selected covariates in ${\boldsymbol X}_1$ and ${\boldsymbol X}_2$ (see Web Appendix G), denoted $\mathbb {Z}_{\textrm {C,cov}}$; and (iii) the test statistic of Kidwell and Wahed (2013), denoted $\mathbb {Z}_{\textrm {KW}}$, which uses known randomization probabilities. With $\mathcal {D}= \lbrace d^1, d^2, d^3, d^4\rbrace$, $D=4$, the set of all embedded regimes, all tests strongly reject $H_0$, consistent with the estimated survival functions. We also took $\mathcal {D}$ with $D=2$ to obtain pairwise comparisons; because the components of the test statistic of Kidwell and Wahed (2013) address pairwise comparisons (see Web Appendix F), $\mathbb {Z}_{\textrm {KW}}$ in Table 3 represents tests based on (linear combinations of) the relevant components. The proposed test statistics incorporating covariates are almost always largest, consistent with the expected increase in power. For the key pairwise comparison of “shared path” Regimes 3 and 4 starting with ATRA + arsenic trioxide, the proposed test statistics yield evidence of a difference, supporting further investigation of the use of ATRA+Mtx+MP as maintenance following this consolidation therapy.

**TABLE 3:** Test statistics and corresponding *P*-values (in parentheses) for comparisons of embedded regimes as indicated in $\mathcal {D}$ in North American Leukemia Intergroup Study C9710.

$\mathcal {D}$	$\mathbb {Z}_{\textrm {C,nocov}}$	$\mathbb {Z}_{\textrm {C,cov}}$	$\mathbb {Z}_{\textrm {KW}}$
$\lbrace d^1, d^2, d^3, d^4\rbrace$	25.990 ($< 0.0001$)	28.817 ($< 0.0001$)	23.425 ($< 0.0001$)
$\lbrace d^3,d^4\rbrace$	3.671 (0.055)	4.241 (0.040)	1.344 (0.246)
$\lbrace d^1,d^2\rbrace$	1.135 (0.287)	2.220 (0.138)	0.832 (0.362)
$\lbrace d^2,d^4\rbrace$	14.638 (0.0001)	14.605 (0.0001)	13.277 (0.0003)
$\lbrace d^1,d^4\rbrace$	20.109 ($< 0.0001$)	24.717 ($< 0.0001$)	21.154 ($< 0.0001$)

$\mathbb {Z}_{\textrm {C,nocov}}$ and $\mathbb {Z}_{\textrm {C,cov}}$ are the proposed test statistic without and with incorporation of covariates, respectively, and using the bias-corrected covariance matris; and $\mathbb {Z}_{\textrm {KW}}$ is the appropriate test statistic based on the approach of Kidwell and Wahed (2013). For all test statistics, *P*-values were obtained from the $\chi ^2$ distribution with 3 or 1 degree(s) of freedom as appropriate.

## 5 DISCUSSION

We have developed a logrank-type test to compare the survival distributions of a time-to-event outcome if the patient population were to receive treatment according to each treatment regime within a specified set of regimes based on the data from a SMART. The test can be modified to incorporate covariate information to enhance efficiency. The test is valid under assumptions analogous to those underlying the standard logrank test for comparing single-stage treatments. The test achieves the nominal level under null hypotheses, and incorporation of covariates associated with the outcome leads to increases in power.

We have focused on SMARTs here, as we believe that an obstacle to formal study of multistage treatment regimes in chronic disease (and especially cancer) research is the lack of general methods for SMARTs with a time-to-event outcome. Accordingly, we adopt parametric models for the treatment assignment probabilities, which is sufficient for estimating randomization probabilities, and the standard assumption of independent censoring, so that no model for the censoring distribution is required. Our framework also accommodates data from an observational study, which requires modeling of treatment assignment probabilities and more complex censoring mechanisms; refinements to incorporate nonparametric modeling of these nuisance quantities is an important topic for future development.

## Supplementary Material

ujae139_Supplemental_Files Web Appendices A–G, referenced in Sections 2–4 and R code implementing the simulations reported in Section 3, are available with this paper at the *Biometrics* website at Oxford Academic.

## Data Availability

Data sharing is not applicable to this article, as the data are proprietary.
