# Supplementary material for: A generalized logrank-type test for comparison of treatment regimes in sequential multiple assignment randomized trials
Source: Biometrics. 2024 Nov 30;80(4):ujae139. doi: 10.1093/biomtc/ujae139 (PMC11636965; doi:10.1093/biomtc/ujae139)
Supplement: ujae139_Supplemental_Files — Web Appendices A–G, referenced in Sections 2–4 and R code implementing the simulations reported in Section 3, are available with this paper at the Biometrics website at Oxford Academic. [file ujae139_supplemental_files.zip › SupplementaryMaterial_LR_SMARTS_TsiatisDavidian_final.pdf]

BIOMETRICS 000, 000–000 DOI: 000
000 0000

Supplementary Material for "A Generalized Logrank-type Test for Comparison of Treatment Regimes in Sequential Multiple Assignment Randomized Trials"

Anastasios A. Tsiatis* and Marie Davidian**

Department of Statistics, North Carolina State University, Raleigh, NC, USA.

**email:* tsiatis@ncsu.edu

***email:* davidian@ncsu.edu

This paper has been submitted for consideration for publication in *Biometrics*

Web Appendix A: Representation in Terms of Observed Data

A.1. Example of form of $C(u, d)$ and $\pi(u, d)$

We demonstrate the form of $C(u, d)$ in (**??**) and $\pi(u, d)$ in (**??**) of the main paper in the case of the SMART in Figure **??** of the main paper, with $K = 2$. Consider the embedded regime $d = (d_1, d_2)$ that gives Trt a at Decision 1, and, if the event does not occur before Decision 2, gives Trt b if an individual is a responder and Trt c if not. Thus, for this regime, $d_1(\boldsymbol{h}_1)$ returns Trt a for all $\boldsymbol{h}_1$, and $I\{A_1 = d_1(\boldsymbol{H}_1)\} = I(A_1 = a)$ and $\omega_1\{\boldsymbol{H}_1, d_1(\boldsymbol{H}_1)\} = P(A_1 = a)$, the probability of being randomized to Trt a at stage 1. Thus, if $\kappa = 1$, as $\mathcal{T}_1 = 0$, $C(u, d) = I(A_1 = a)$ and $\pi(u, d) = P(A_1 = a)$ for all $u \geqslant 0$. If $\kappa = 2$, letting $R_2 = 1$ (0) if an individual responds (does not respond) to stage 1 treatment, where R_2 is a function of $\boldsymbol{X}_2$ and thus $\boldsymbol{H}_2$, $d_2(\boldsymbol{h}_2)$ is a composite of two subset-specific rules: one of these selects Trt c for $\boldsymbol{h}_2$ such that $r_2 = 0, a_1 = a$, and the other selects Trt b for $\boldsymbol{h}_2$ such that $r_2 = 1, a_1 = a$. Thus, $I\{A_2 = d_2(\boldsymbol{H}_2)\} = R_2 I(A_2 = b) + (1 - R_2) I(A_2 = c)$, and $\omega_2\{\boldsymbol{H}_2, d_2(\boldsymbol{H}_2)\} = R_2 P(A_2 = b | A_1 = a, R_2 = 1, \kappa = 2) + (1 - R_2) P(A_2 = c | A_1 = a, R_2 = 0, \kappa = 2)$, where $P(A_2 = a_2 | A_1 = a, R_2 = r, \kappa = 2)$ is the probability of being randomized to Trt a_2 at stage 2 after being randomized to Trt a at stage 1 and reaching stage 2 as a responder ($r = 1$) or nonresponder ($r = 0$). Then if $\kappa = 2$, for $u \geqslant 0$, $C(u, d) = I(A_1 = a)\big[I\{\mathcal{T}_2 > u\} + I\{\mathcal{T}_2 \leqslant u\}\{R_2 I(A_2 = b) + (1 - R_2) I(A_2 = c)\}\big]$ and $\pi(u, d) = P(A_1 = a)\big[I\{\mathcal{T}_2 > u\} + I\{\mathcal{T}_2 \leqslant u\}\{R_2 P(A_2 = b | A_1 = a, R_2 = 1, \kappa = 2) + (1 - R_2) P(A_2 = c | A_1 = a, R_2 = 0, \kappa = 2)\}\big]$.

A.2. Demonstration of (??)

We wish to show that, under H_0 and the identifiability assumptions in Section **??** of the main paper, for $u \geqslant 0$, an inverse probability weighted observed data analog to (**??**) of the

main paper, that is,

$$\sum_{i=1}^{n}\sum_{j=1}^{D} w(u,d^j)\{dN_i^*(u,d^j) - d\Lambda_0(u)Y_i^*(u,d^j)\} = 0, \tag{A.1}$$

is given by (**??**) of the main paper,

$$\sum_{i=1}^{n}\sum_{j=1}^{D} \Omega_i(u,d^j)\{dN_i(u) - d\Lambda_0(u)Y_i(u)\} = 0, \quad \Omega(u,d) = \frac{C(u,d)I(U \geqslant u)}{\pi(u,d)K_c\{u|H(u)\}}w(u,d), \tag{A.2}$$

and that the "score equation" for β_j in terms of potential outcomes,

$$\sum_{i=1}^{n}\int_0^\infty w(u,d^j)\{dN_i^*(u,d^j) - d\Lambda_0(u)Y_i^*(u,d^j)\} = 0, \tag{A.3}$$

has observed data analog

$$\sum_{i=1}^{n}\int_0^\infty \Omega_i(u,d^j)\{dN_i(u) - d\Lambda_0(u)Y_i(u)\} = 0 \tag{A.4}$$

for $j = 1,\dots,D-1$. Multiplying (A.1)-(A.4) by n^{-1}, it suffices to show that the limit in probability of (A.1) is the same as that for (A.2), and similarly for (A.3) and (A.4). Because the weights $w(u,d^j)$, $j = 1,\dots,D-1$, are fixed constants for each u and d^j, we disregard them and thus show that, similar to Tsiatis et al. (2020, Section 8.4.3) and using the definition of $\Omega(u,d)$ in (A.2), for regime d,

$$E\left[\frac{C(u,d)I(U \geqslant u)}{\pi(u,d)K_c\{u|H(u)\}}\{dN(u) - d\Lambda_0(u)Y(u)\}\right] = E\{dN^*(u,d) - d\Lambda_0(u)Y^*(u,d)\}.$$

Clearly, it suffices to show that

$$E\left[\frac{C(u,d)I(U \geqslant u)}{\pi(u,d)K_c\{u|H(u)\}}\{dN(u) - d\Lambda_0(u)Y(u)\}\middle| \mathcal{W}^*\right] = dN^*(u,d) - d\Lambda_0(u)Y^*(u,d). \tag{A.5}$$

The integrand on the left hand side of (A.5) can be written as

$$\frac{C(u,d)I(U \geqslant u)}{\pi(u,d)K_c\{u|H(u)\}}I(U \geqslant u)\{dN(u) - d\Lambda_0(u)Y(u)\}.$$

By the consistency assumption, this expression can then be written as

$$\frac{C(u,d)I(U \geqslant u)}{\pi(u,d)K_c\{u|H(u)\}}\{dN^*(u,d) - d\Lambda_0(u)Y^*(u,d)\}$$
$$= \frac{C(u,d)I(U \geqslant u)}{\pi(u,d)K_c\{u|H(u)\}}I\{T^*(d) \geqslant u\}\{dN^*(u,d) - d\Lambda_0(u)Y^*(u,d)\},$$

so that the left hand side of (A.5) becomes

$$\{dN^*(u,d) - d\Lambda_0(u)Y^*(u,d)\}E\left[\frac{C(u,d)I(U \geqslant u)}{\pi(u,d)K_c\{u|H(u)\}}\middle| \mathcal{W}^*, T^*(d) \geqslant u\right]. \tag{A.6}$$

Thus, from (A.6), we need to show that

$$E\left[\frac{C(u,d)I(U \geqslant u)}{\pi(u,d)K_c\{u|H(u)\}}\middle| \mathcal{W}^*, T^*(d) \geqslant u\right] = 1, \quad u \geqslant 0. \tag{A.7}$$

We argue heuristically that (A.7) holds using a discrete time approximation and induction. Assume that, rather than occurring at any point in continuous time, decision points can occur only at equally-spaced time points; e.g., at any whole second, but not at fractions of a second. Partition the interval $[0,u]$ into an equally-spaced grid with grid points v_m, $m = 0, \ldots, M$, where $v_0 = 0$ and $v_M = u$, such that any decision points occurring in the interval $[0,u]$ fall on the grid, and intervals between grid points are of length $\delta v = u/M$. Define $A(v_m)$ to be the treatment option that is received at time v_m from among the feasible options at v_m. If v_m corresponds to one of the times $\mathcal{T}_1, \ldots, \mathcal{T}_\kappa$ at which decision points are reached, then $A(v_m)$ is the treatment assigned at v_m; if v_m does not correspond to one these times, then $A(v_m)$ is equal to the sole option "do nothing." Let $H^-(v_m)$ to be the history through time v_m up to but not including $A(v_m)$; i.e., $H^-(v_m)$ comprises all prior covariate information, treatments, and times of decision points up to and including v_m that would be used to determine treatment if v_m were a decision point. Thus, with $H(u)$ defined as in the main paper, $H(v_m) = \{H^-(v_m), A(v_m)\}$. Then, for the regime d of interest, define $d\{H^-(v_m)\}$ to be the "rule" that assigns treatment at time v_m based on the history $H^-(v_m)$ at time v_m; if v_m corresponds to one of these times, $d\{H^-(v_m)\}$ assigns the treatment indicated by the relevant rule in d given the history; if not, $d\{H^-(v_m)\}$ assigns "do nothing." Define the

following quantities:

$$\begin{aligned}
C^M(u,d) &= \prod_{m=0}^{M} I\big[A(v_m) = d\{H^-(v_m)\}\big] \\
\pi^M(u,d) &= \prod_{m=0}^{M} P\big[A(v_m) = d\{H^-(v_m)\}|H^-(v_m), U \geqslant v_m\big] \\
K_c^M\{u|H(u)\} &= \prod_{m=0}^{M} P\{U \geqslant v_{m+1}|H^-(v_m), A(v_m), U \geqslant v_m, T^*(d) \geqslant u\} \qquad \text{(A.8)} \\
&= \prod_{m=0}^{M} P\{U \geqslant v_{m+1}|H(v_m), U \geqslant v_m, T^*(d) \geqslant u\} \qquad \text{(A.9)} \\
I^M(u) &= \prod_{m=0}^{M} I(U \geqslant v_{m+1}). \qquad \text{(A.10)}
\end{aligned}$$

In (A.9) and (A.10), v_{M+1} is the next time point outside of the interval $[0,u]$, i.e., $u + \delta u = u(1+1/M)$. Evidently, $C^M(u,d) = C(u,d)$ and $\pi^M(u,d) = \pi(u,d)$. In (A.9), when $T^*(d) \geqslant u$,

$$\begin{aligned}
&P\{U \geqslant v_{m+1}|U \geqslant v_m, H(v_m), T^*(d) \geqslant u\} \\
&\qquad = 1 - P\{v_m \leqslant U < v_{m+1}|U \geqslant v_m, H(v_m), T^*(d) \geqslant u\}. \qquad \text{(A.11)}
\end{aligned}$$

Because $v_m \leqslant u$, $m = 0, \dots, M$ and $T^*(d) \geqslant u$, the only way that U can fall between v_m and v_{m+1} for $m = 0, \dots, M-1$ is if an individual is censored, in which case (A.11) becomes

$$1 - P\{v_m \leqslant U < v_{m+1}, \Delta = 0|U \geqslant v_m, H(v_m), T^*(d) \geqslant u\}. \qquad \text{(A.12)}$$

For $m = M$, the probability in (A.11) converges to 0 as $M \to \infty$. Thus, with the assumption of noninformative censoring, (A.12) is approximately equal to $1 - \lambda_c\{v_m|H(v_m)\}\delta v$, so that

$$K_c^M\{u|H(u)\} \approx \exp\left[-\int_0^u \lambda_c\{v|H(v)\}\,dv\right] = K_c\{u|H(u)\}$$

for large M. Likewise, if U is continuous, $I^M(u)$ converges almost surely to $I(U \geqslant u)$ as $M \to \infty$.

Now define the sequence of random variables

$$\mathcal{Z}^M = \frac{C^M(u,d)I^M(u)}{\pi^M(u,d)K_c^M\{u|H(u)\}}.$$

From the considerations above, under reasonable regularity conditions, $\mathcal{Z}^M$ converges almost

surely as $M \to \infty$ to

$$\frac{C(u,d)I(U \geqslant u)}{\pi(u,d)K_c\{u|H(u)\}}.$$

Thus, if we can show that

$$E\left\{\mathcal{Z}^M|\mathcal{W}^*, T^*(d) \geqslant u\right\} = 1 \text{ a.s.}, \tag{A.13}$$

then under regularity conditions the desired result (A.7) holds.

We demonstrate (A.13) by induction. Specifically, define the partial products

$$\mathcal{Z}^M(t) = \prod_{m=0}^{t} \frac{I\big[A(v_m) = d\{H^-(v_m)\}\big]I(U \geqslant v_{m+1})}{P\big[A(v_m) = d\{H^-(v_m)\}|H^-(v_m), U \geqslant v_m\big]P\{U \geqslant v_{m+1}|U \geqslant v_m, H(v_m), T^*(d) \geqslant u\}}$$

for $t = 0, \ldots, M$, so that $\mathcal{Z}^M(M) = \mathcal{Z}^M$ and

$$\mathcal{Z}^M(0) = \frac{I\{A_1 = d_1(\boldsymbol{H}_1)\}I(U \geqslant v_1)}{P\{A_1 = d_1(\boldsymbol{H}_1)|\boldsymbol{H}_1\}P\{U \geqslant v_1|\boldsymbol{H}_1, A_1, T^*(d) \geqslant u\}}.$$

Assume that all probabilities in these expressions are positive. Using the SRA and the noninformative censoring assumption

$$\begin{aligned}
&E\{\mathcal{Z}^M(0)|\mathcal{W}^*, T^*(d) \geqslant u\} = E\big[E\{\mathcal{Z}^M(0)|\boldsymbol{H}_1, A_1, \mathcal{W}^*, T^*(d) \geqslant u\}|\mathcal{W}^*, T^*(d) \geqslant u\big] \\
&\quad = E\Bigg(\frac{I\{A_1 = d_1(\boldsymbol{H}_1)\}}{P\{A_1 = d_1(\boldsymbol{H}_1)|\boldsymbol{H}_1\}} \\
&\qquad\quad \times\ E\left[\frac{I(U \geqslant v_1)}{P\{U \geqslant v_1|\boldsymbol{H}_1, A_1, T^*(d) \geqslant u\}}\middle| \boldsymbol{H}_1, A_1, \mathcal{W}^*, T^*(d) \geqslant u\right]\Bigg| \mathcal{W}^*, T^*(d) \geqslant u\Bigg) \\
&\quad = E\left[\frac{I\{A_1 = d_1(\boldsymbol{H}_1)\}}{P\{A_1 = d_1(\boldsymbol{H}_1)|\boldsymbol{H}_1\}}\middle| \mathcal{W}^*, T^*(d) \geqslant u\right] \qquad\qquad (A.14) \\
&\quad = E\left(E\left[\frac{I\{A_1 = d_1(\boldsymbol{H}_1)\}}{P\{A_1 = d_1(\boldsymbol{H}_1)|\boldsymbol{H}_1\}}\middle| \boldsymbol{H}_1, \mathcal{W}^*, T^*(d) \geqslant u\right]\middle| \mathcal{W}^*, T^*(d) \geqslant u\right) \\
&\quad = 1 \text{ a.s.}
\end{aligned}$$

Thus, if we can show that

$$E\{\mathcal{Z}^M(t)|\mathcal{W}^*, T^*(d) \geqslant u\} = E\{\mathcal{Z}^M(t-1)|\mathcal{W}^*, T^*(d) \geqslant u\} \text{ a.s.}, \tag{A.15}$$

(A.13) holds by induction.

To show (A.15), note that

$$E\{\mathcal{Z}^M(t)|\mathcal{W}^*, T^*(d) \geqslant u\} = E\big[E\{\mathcal{Z}^M(t)|H^-(v_t), U \geqslant v_t, \mathcal{W}^*, T^*(d) \geqslant u\}|\mathcal{W}^*, T^*(d) \geqslant u\big] \text{ a.s.}$$

The inner expectation is equal almost surely to

$$\mathcal{Z}^M(t) = \mathcal{Z}^M(t{-}1)\left\{\frac{E\big(I\big[A(v_t) = d\{H^-(v_t)\}\big]I(U \geqslant v_{t+1})|H^-(v_t), U \geqslant v_t, \mathcal{W}^*, T^*(d) \geqslant u\big)}{P\big[A(v_t) = d\{H^-(v_t)\}|H^-(v_t), U \geqslant v_t\big]P\{U \geqslant v_{t+1}|U \geqslant v_t, H(v_t), T^*(d) \geqslant u\}}\right\}$$

Then using the SRA and the noninformative censoring assumption, by an argument analogous to that in (A.14), the term in large braces is equal to 1 almost surely, and the result follows.

Web Appendix B: More Complex Censoring and Modeling/Estimation of $\omega_k(\boldsymbol{h}_k, a_k)$

B.1. Dependence of censoring on history

In the main paper, we adopt the assumption of noninformative censoring and further assume that censoring is independent of $H(u)$, or, if dependent on $H(u)$, depends on $H(u)$ only through stage 1 (baseline) treatment assignment. As we note in the main paper, these assumptions are analogous to those that underlie the standard logrank test for a conventional multi-arm clinical trial.

In some settings, the analyst may believe that censoring, although noninformative as defined in the main paper (so depending only on $H(u)$ and not on potential outcomes), depends on $H(u)$ in a more complex fashion. This feature may be particularly of concern if the data arise from an observational study. For example, censoring at u may be associated with baseline covariates, covariates that evolve over time, timing of decisions, or treatments at stage 2 and higher contained in $H(u)$. The formulation of the general testing procedure outlined in Section **??** of the main paper allows for this possibility. Namely, instead of adopting the simplification (**??**) of (**??**) of the main paper and taking $w(u, d^j) = K_c(u)$, $j = 1, \ldots, D$, one can maintain the more general expression for $\Omega(u, d)$ as in (**??**) and adopt a model for the censoring survival function $K_c\{u|H(u)\}$. The model should be chosen to reflect beliefs about the censoring process as a function of past history.

One approach would be to adopt an appropriate semiparametric proportional hazards model for the cause-specific hazard of censoring $\lambda_c\{u|H(u)\}$ given in Section **??** of the main paper fitted using maximum partial likelihood based on the "data" $\{H_i(U_i), U_i, 1-\Delta_i\}$, $i = 1, \ldots, n$, where $H(U_i)$ is the history to U_i for subject i; see, for example, Tsiatis et al. (2020, Section 8.3.3, pages 381–382) for discussion of considerations for developing and fitting such models. A possible concern with this approach is that the chosen proportional hazards model may be misspecified. Substitution of the resulting fitted misspecified model for $K_c\{u|H(u)\}$ in our inverse probability weighted test statistic could lead to a biased test. Accordingly, one may wish to consider adopting a nonparametric model for $K_c\{u|H(u)\}$, possibly through a flexible basis representation for the logarithm of $\lambda_c\{u|H(u)\}$. Ertefaie, Hejazi, and var der Laan (2023) and var der Laan, Benkeser, and Cai (2022) propose nonparametric estimation of $K_c\{u|H(u)\}$ via use of the highly adaptive lasso; see Ertefaie et al. (2023, Section 7) and var der Laan et al. (2022, Section 7). The results of these authors suggest that, with proper undersmoothing, estimation of $K_c\{u|H(u)\}$ via this approach in our method should yield a consistent test with increased efficiency.

Two issues must be addressed under these conditions. One is that account must be taken of the fact that $K_c\{u|H(u)\}$ has been estimated in obtaining the asymptotic covariance matrix of $\mathfrak{T}$ or $\mathfrak{T}(\widehat{\gamma})$. We conjecture that one can estimate the covariance matrix via a nonparametric bootstrap and use the bootstrap estimator to form the test statistic. This approach is a topic for future research. In addition, the weight functions $w(u, d^j)$, $j = 1, \ldots, D$, must be specified. One choice is to set these identically equal to 1. A possibly better alternative is to choose these in the spirit of stabilizing weights as in, for example, Tsiatis and Davidian (2022); namely, take $w(u, d^j)$ to be the fitted censoring model evaluated at the averages of components of the history. Again, choice of the weights under these conditions is a topic for future investigation.

Note that, if one believes that censoring does not depend on $H(u)$ or depends on $H(u)$ only through stage 1 treatment, one could estimate $K_c(u)$ (possibly separately by stage 1 treatment as dictated by d^j) using the Kaplan-Meier estimator. However, there is no advantage over the approach in the main paper, as the weights can be chosen to be equal to the corresponding estimator, in which case $\Omega(u, d^j)$ reduces to (**??**).

B.2. Modeling and estimation of $\omega_k(\boldsymbol{h}_k, a_k)$

In Section **??** of the main paper, the developments leading to the test statistic $\mathbb{Z} = n^{-1}\boldsymbol{\mathfrak{T}}^T\widehat{\boldsymbol{\Sigma}}^{-}\boldsymbol{\mathfrak{T}}$ take the probabilities of treatment assignment $\omega_k(\boldsymbol{h}_k, a_k) = P(A_k = a_k|\boldsymbol{H}_k = \boldsymbol{h}_k, \kappa \geqslant k)$, $k = 1, \ldots, K$, to be known functions of $\boldsymbol{h}_k$ and a_k. Thus, in particular, the form of the asymptotic covariance matrix $\boldsymbol{\Sigma}$ of $\boldsymbol{\mathfrak{T}}$ and thus the estimator $\widehat{\boldsymbol{\Sigma}}$ is derived under the condition that these probabilities are known, so that the test should achieve the chosen nominal level of significance under H_0. When the observed data $\mathcal{O}_i$, $i = 1, \ldots, n$, are from a SMART, these probabilities are the randomization probabilities, which of course are known by design. When the data are from an observational study, these probabilities are unknown; thus, they must be modeled and estimated. As noted in the main paper and discussed in detail in Web Appendix D, estimating these probabilities by fitting correctly specified parametric models at each decision point and substituting the estimators in (**??**) of the main paper and thus in $\boldsymbol{\mathfrak{T}}$ will lead to a test that is at least as powerful as that obtained based on known probabilities.

Here, we discuss considerations for modeling and estimating the treatment assignment probabilities $\omega_k(\boldsymbol{h}_k, a_k)$, $k = 1, \ldots, K$. The standard approach in practice is to adopt a logistic regression model in the case of two treatment options or an appropriate multinomial logistic (polytomous) regression model for more than two treatment options $\omega_k(\boldsymbol{h}_k, a_k; \boldsymbol{\gamma}_k)$, say, in terms of a finite-dimensional parameter $\boldsymbol{\gamma}_k$. By a "correctly specified" parametric

model at each Decision k, we mean that the model is such that there is a value of the parameter $\boldsymbol{\gamma}_k$, $\boldsymbol{\gamma}_{k,0}$, say, such that $\omega_k(\boldsymbol{h}_k, a_k; \boldsymbol{\gamma}_{k,0})$ yields the true treatment assignment probabilities. In a SMART, because the randomization probabilities are known, specification of correcly specified models $\omega_k(\boldsymbol{h}_k, a_k; \boldsymbol{\gamma}_k)$ that contain the true randomization probabilities in this sense is straightforward. We demonstrate in the following example.

Example 1: Two-stage SMART. Consider the two-stage SMART in Figure **??** of the main paper, with two feasible treatment options at each point of randomization. Here, $\omega_1(\boldsymbol{h}_1, a_1) = P(A_1 = a_1|\boldsymbol{H}_1 = \boldsymbol{h}_1, \kappa \geqslant 1) = P(A_1 = a_1)$ can be represented as a logistic regression model

$$\omega_1(\boldsymbol{h}_1, a_1; \gamma_1) = \left\{\frac{\exp(\gamma_1)}{1+\exp(\gamma_1)}\right\}^{I(a_1=1)} \left\{\frac{1}{1+\exp(\gamma_1)}\right\}^{I(a_1=0)},$$

which can be fitted by maximum likelihood (ML) using the data A_{1i}, $i = 1, \ldots, n$; see below. It is straightforward that the ML estimator $\widehat{\gamma}_1$ is such that $\text{expit}(\widehat{\gamma}_1)$ is equal to the sample proportion of subjects randomized at stage 1 to Trt 1, where $\text{expit}(s) = e^s/(1 + e^s)$. At stage 2, letting r_2 be the component/function of $\boldsymbol{h}_2$ indicating response status, $\omega_2(\boldsymbol{h}_2, a_2) = P(A_2 = a_2|\boldsymbol{H}_2 = \boldsymbol{h}_2, \kappa \geqslant 2)$ can be represented as

$$\begin{aligned}\omega_2(\boldsymbol{h}_2, a_2; \boldsymbol{\gamma}_2) = &\left\{\frac{\exp(\gamma_{21})}{1+\exp(\gamma_{21})}\right\}^{I(a_1=0,r_2=1,a_2=2)} \left\{\frac{1}{1+\exp(\gamma_{21})}\right\}^{I(a_1=0,r_2=1,a_2=3)}\\
&\times \left\{\frac{\exp(\gamma_{22})}{1+\exp(\gamma_{22})}\right\}^{I(a_1=0,r_2=0,a_2=2)} \left\{\frac{1}{1+\exp(\gamma_{22})}\right\}^{I(a_1=0,r_2=0,a_2=4)}\\
&\times \left\{\frac{\exp(\gamma_{23})}{1+\exp(\gamma_{23})}\right\}^{I(a_1=1,r_2=1,a_2=2)} \left\{\frac{1}{1+\exp(\gamma_{23})}\right\}^{I(a_1=1,r_2=1,a_2=5)}\\
&\times \left\{\frac{\exp(\gamma_{24})}{1+\exp(\gamma_{24})}\right\}^{I(a_1=1,r_2=0,a_2=3)} \left\{\frac{1}{1+\exp(\gamma_{24})}\right\}^{I(a_1=1,r_2=0,a_2=5)}.\end{aligned}$$

This model can be fitted by ML to the data $\{\boldsymbol{H}_{2i}, A_{2i}\}$ for all i for whom $\kappa_i = 2$ (see below). The ML estimator $\widehat{\boldsymbol{\gamma}}_2 = (\widehat{\gamma}_{21}, \widehat{\gamma}_{22}, \widehat{\gamma}_{23}, \widehat{\gamma}_{24})^T$ is such that, for example, $\text{expit}(\widehat{\gamma}_{21})$ is equal to the sample proportion of subjects who were randomized at stage 1 to Trt 0, reached stage 2 without experiencing the event or censoring, responded to Trt 0, and were randomized to Trt 2.

The ML estimator for $\boldsymbol{\gamma} = (\gamma_1, \boldsymbol{\gamma}_2^T)^T$ is found by maximizing the loglikelihood for $\boldsymbol{\gamma}$; the likelihood contribution for a single subject is given by

$$\begin{aligned}
&\frac{\exp\{\gamma_1 I(A_1 = 1)\}}{1+\exp(\gamma_1)} \\
&\times \left(\left[\frac{\exp\{\gamma_{21} I(A_2 = 2)\}}{1+\exp(\gamma_{21})} \right]^{I(A_1=0,R_2=1)} \left[\frac{\exp\{\gamma_{22} I(A_2 = 2)\}}{1+\exp(\gamma_{22})} \right]^{I(A_1=0,R_2=0)} \right. \\
&\times \left. \left[\frac{\exp\{\gamma_{23} I(A_2 = 2)\}}{1+\exp(\gamma_{23})} \right]^{I(A_1=1,R_2=1)} \left[\frac{\exp\{\gamma_{24} I(A_2 = 3)\}}{1+\exp(\gamma_{24})} \right]^{I(A_1=1,R_2=0)} \right)^{I(\kappa=2)} .
\end{aligned} \tag{B.1}$$

It is straightforward that the associated score vector is

$$\boldsymbol{S}_\gamma(\boldsymbol{\gamma}) = \begin{pmatrix} \{I(A_1 = 1) - \text{expit}(\gamma_1)\} \\ I(A_1 = 0, R_2 = 1, \kappa = 2)\{I(A_2 = 2) - \text{expit}(\gamma_{21})\} \\ I(A_1 = 0, R_2 = 0, \kappa = 2)\{I(A_2 = 2) - \text{expit}(\gamma_{22})\} \\ I(A_1 = 1, R_2 = 1, \kappa = 2)\{I(A_2 = 2) - \text{expit}(\gamma_{23})\} \\ I(A_1 = 1, R_2 = 0, \kappa = 2)\{I(A_2 = 3) - \text{expit}(\gamma_{24})\} \end{pmatrix} \tag{B.2}$$

When the data are from an observational study, the analyst must posit models $\omega_k(\boldsymbol{h}_k, a_k; \boldsymbol{\gamma}_k)$, $k = 1, \ldots, K$, that incorporate possible dependence of the probabilities of treatment on past history both to acknowledge the likely dependence of treatment assignment on history and to account for possible confounding. As noted in the main paper, one approach, which has been used extensively in practice, is to posit parametric models to represent the treatment assignment probabilities. We demonstrate in the following example.

Example 2: Observational study with two decision points. Consider a setting with $K = 2$ stages where subjects were observed to receive one of two treatments, coded as 0 and 1, at stage 1, and, among those who reached stage 2 without having experienced the event or censoring, responders continued on stage 1 treatment and nonresponders were observed to receive one of two treatments, also coded as 0 and 1 (but not necessarily the same as those

at stage 1). Typical logistic regression models are of the form

$$\omega_1(\boldsymbol{h}_1, a_1; \gamma_1) = \left\{ \frac{\exp(\boldsymbol{\gamma}_1^T \widetilde{\boldsymbol{h}}_1)}{1+\exp(\boldsymbol{\gamma}_1^T \widetilde{\boldsymbol{h}}_1)} \right\}^{I(a_1=1)} \left\{ \frac{1}{1+\exp(\boldsymbol{\gamma}_1^T \widetilde{\boldsymbol{h}}_1)} \right\}^{I(a_1=0)},$$

$$\omega_2(\boldsymbol{h}_2, a_2; \boldsymbol{\gamma}_2) = \left\{ \frac{\exp(\boldsymbol{\gamma}_{21}^T \widetilde{\boldsymbol{h}}_2)}{1+\exp(\boldsymbol{\gamma}_{21}^T \widetilde{\boldsymbol{h}}_2)} \right\}^{I(a_1=0, r_2=0, a_2=1)} \left\{ \frac{1}{1+\exp(\boldsymbol{\gamma}_{21}^T \widetilde{\boldsymbol{h}}_2)} \right\}^{I(a_1=0, r_2=0, a_2=0)}$$
$$\times \left\{ \frac{\exp(\boldsymbol{\gamma}_{22}^T \widetilde{\boldsymbol{h}}_2)}{1+\exp(\boldsymbol{\gamma}_{22}^T \widetilde{\boldsymbol{h}}_2)} \right\}^{I(a_1=1, r_2=0, a_2=1)} \left\{ \frac{1}{1+\exp(\boldsymbol{\gamma}_{22}^T \widetilde{\boldsymbol{h}}_2)}) \right\}^{I(a_1=1, r_2=0, a_2=0)},$$

where $\widetilde{\boldsymbol{h}}_k$ is a vector of functions of elements of $\boldsymbol{h}_k$, ordinarily with a "1" in the first position to yield an "intercept" term, $k = 1, 2$, so could include, for example, interactions, polynomial terms or splines, and so on. Analogous to (B.1), the contribution of a single subject to the likelihood for $\boldsymbol{\gamma} = (\boldsymbol{\gamma}_1^T, \boldsymbol{\gamma}_2^T)^T$ is given by

$$\begin{aligned} &\frac{\exp\{\boldsymbol{\gamma}_1^T \widetilde{\boldsymbol{H}}_1 I(A_1 = 1)\}}{1+\exp(\boldsymbol{\gamma}_1^T \widetilde{\boldsymbol{H}}_1)} \left[\frac{\exp\{\boldsymbol{\gamma}_{21}^T \widetilde{\boldsymbol{H}}_2 I(A_2 = 1)\}}{1+\exp(\boldsymbol{\gamma}_{21}^T \widetilde{\boldsymbol{H}}_2)} \right]^{I(A_1=0, R_2=0, \kappa=2)} \\ &\times \left[\frac{\exp\{\boldsymbol{\gamma}_{22}^T \widetilde{\boldsymbol{H}}_2 I(A_2 = 1)\}}{1+\exp(\boldsymbol{\gamma}_{22}^T \widetilde{\boldsymbol{H}}_2)} \right]^{I(A_1=1, R_2=0, \kappa=2))}, \end{aligned} \tag{B.3}$$

and the ML estimator for $\boldsymbol{\gamma}$ maximizes in $\boldsymbol{\gamma}$ the corresponding loglikelihood. The associated score vector is

$$\boldsymbol{S}_\gamma(\boldsymbol{\gamma}) = \begin{pmatrix} \{I(A_1 = 1) - \text{expit}(\boldsymbol{\gamma}_1^T \widetilde{\boldsymbol{H}}_1)\} \widetilde{\boldsymbol{H}}_1 \\ I(A_1 = 0, R_2 = 0, \kappa = 2)\{I(A_2 = 1) - \text{expit}(\boldsymbol{\gamma}_{21}^T \widetilde{\boldsymbol{H}}_2)\} \widetilde{\boldsymbol{H}}_2 \\ (A_1 = 1, R_2 = 0, \kappa = 2)\{I(A_2 = 1) - \text{expit}(\boldsymbol{\gamma}_{22}^T \widetilde{\boldsymbol{H}}_2)\} \widetilde{\boldsymbol{H}}_2 \end{pmatrix}. \tag{B.4}$$

Unlike in a SMART, a natural concern with observational data, where the treatment assignment mechanism is unknown and could be a complex function of past history, is that the posited parametric models may be not be correctly specified in the above sense. Analogous to the discussion in the previous section on modeling the censoring distribution when censoring is assumed noninformative but not independent, substitution of the fitted treatment assignment probabilities based on misspecified models in our inverse probability weighted test statistic could lead to a biased test.

As mentioned in Section **??** of the main paper, adopting of flexible, nonparametric representations for the treatment assignment probabilities may be more appropriate, and the use of various machine learning techniques, such as boosting, random forests, neural networks, and others, for this purpose has been proposed; see, for example, McCaffrey, Ridgeway, and Morral (2004), Lee, Lessler, and Stuart (2010), and Pirracchio, Petersen, and van der Laan (2015). A general concern with such approaches is overfitting of the probabilities and computational complexity (e.g., Alam, Moodie, and Stephens, 2018). Recently, Ertefaie, Hejazi, and var der Laan (2023) propose nonparametric estimation of treatment assignment probabilities for use in inverse probability weighted estimators using the highly adaptive lasso and present results suggesting that, with proper undersmoothing, estimation of $\omega_k(\boldsymbol{h}_k, a_k)$, $k = 1, \ldots, K$, via this approach should yield a consistent test with increased efficiency in our setting. Ertefaie et al. (2023) restrict to inverse weighting in settings analogous to $K = 1$. Topics for future investigation include adaptation of this approach to our proposed test statistics, with general K; and as in the previous section, use of a nonparametric bootstrap to form the test statistic.

Web Appendix C: Representation of $\mathfrak{T}$ as a Sum of iid Terms

We wish to show that $n^{-1/2}\times$ (**??**) in the main paper,

$$n^{-1/2}\mathfrak{T}^j = n^{-1/2}\sum_{i=1}^{n}\int_0^\infty \Omega_i(u, d^j)\{dN_i(u) - d\widehat{\Lambda}_0(u)Y_i(u)\},$$

for each $j = 1, \ldots, D-1$, satisfies (**??**) of the main paper. We begin by adding and subtracting $d\Lambda_0(u)$ in the integral, to obtain

$$\begin{aligned} n^{-1/2}\sum_{i=1}^{n}\int_0^\infty & \Omega_i(u, d^j)\big[dN_i(u) - \{d\widehat{\Lambda}_0(u) + d\Lambda_0(u) - d\Lambda_0(u)\}Y_i(u)\big] \\ &= n^{-1/2}\sum_{i=1}^{n}\int_0^\infty \Omega_i(u, d^j)\{dN_i(u) - d\Lambda_0(u)Y_i(u)\} \\ &\quad - n^{-1/2}\sum_{i=1}^{n}\int_0^\infty \Omega_i(u, d^j)\{d\widehat{\Lambda}_0(u) - d\Lambda_0(u)\}Y_i(u). \end{aligned} \tag{C.1}$$

As in the main paper, defining $d\overline{N}_i(u) = \sum_{j=1}^{D} \Omega_i(u,d^j)dN_i(u)$, $\overline{Y}_i(u) = \sum_{j=1}^{D} \Omega_i(u,d^j)Y_i(u)$, and $\widehat{q}(u,d) = \big\{n^{-1}\sum_{i=1}^{n} \Omega_i(u,d)Y_i(u)\big\}/\big\{n^{-1}\sum_{i=1}^{n} \overline{Y}_i(u)\big\}$, so that

$$d\widehat{\Lambda}_0(u) = \big\{n^{-1}\sum_{i=1}^{n} d\overline{N}_i(u)\big\}/\big\{n^{-1}\sum_{i=1}^{n} \overline{Y}_i(u)\big\},$$

substituting this expression in (C.1) and rearranging and using the definition of $\widehat{q}(u,d^j) = \big\{n^{-1}\sum_{i=1}^{n} \Omega_i(u,d^j)Y_i(u)\big\}/\big\{n^{-1}\sum_{i=1}^{n} \overline{Y}_i(u)\big\}$, (C.1) becomes

$$\begin{aligned} n^{-1/2}\sum_{i=1}^{n}\int_0^\infty & \widehat{q}(u,d^j)\{d\overline{N}_i(u) - d\Lambda_0(u)\overline{Y}_i(u)\} \\ &= n^{-1/2}\sum_{i=1}^{n}\int_0^\infty \widehat{q}(u,d^j)\left\{\sum_{j'=1}^{D}\Omega_i(u,d^{j'})dN_i(u) - d\Lambda_0(u)\sum_{j'=1}^{D}\Omega_i(u,d^{j'})Y_i(u)\right\} \\ &= n^{-1/2}\sum_{i=1}^{n}\sum_{j'=1}^{D}\int_0^\infty \Omega_i(u,d^{j'})\widehat{q}(u,d^j)\{dN_i(u) - d\Lambda_0(u)Y_i(u)\}. \qquad \text{(C.2)} \end{aligned}$$

Thus, using (C.2), we have

$$\begin{aligned} n^{-1/2}\mathfrak{T}^j = n^{-1/2}\sum_{i=1}^{n}\Bigg[&\int_0^\infty \Omega_i(u,d^j)\{dN_i(u) - d\Lambda_0(u)Y_i(u)\} \\ &- \sum_{j'=1}^{D}\int_0^\infty \Omega_i(u,d^{j'})\widehat{q}(u,d^j)\{dN_i(u) - d\Lambda_0(u)Y_i(u)\Bigg]. \qquad \text{(C.3)} \end{aligned}$$

For $j = 1,\ldots,D-1$, under the consistency assumption in the main paper, $\widehat{q}(u,d^j)$ converges in probability as $n\to\infty$ to $q(u,d^j) = E\big\{\Omega(u,d^j)Y^*(u,d^j)\big\}/\sum_{j'=1}^{D} E\big\{\Omega(u,d^{j'})Y^*(u,d^{j'})\big\}$. Then under regularity conditions, $n^{-1/2}$ times (C.3) satisfies

$$\begin{aligned} n^{-1/2}\sum_{i=1}^{n}&\left[\sum_{j'=1}^{D}\int_0^\infty \Omega_i(u,d^{j'})\widehat{q}(u,d^j)\{dN_i(u) - d\Lambda_0(u)Y_i(u)\right] \\ &= n^{-1/2}\sum_{i=1}^{n}\left[\sum_{j'=1}^{D}\int_0^\infty \Omega_i(u,d^{j'})q(u,d^j)\{dN_i(u) - d\Lambda_0(u)Y_i(u)\right] + o_p(1), \end{aligned}$$

leading to (**??**) in the main paper; namely,

$$\begin{aligned} n^{-1/2}\mathfrak{T}^j = n^{-1/2}\sum_{i=1}^{n}\Bigg[&\int_0^\infty \Omega_i(u,d^j)\{dN_i(u) - d\Lambda_0(u)Y_i(u)\} \\ &- \sum_{j'=1}^{D}\int_0^\infty \Omega_i(u,d^{j'})q(u,d^j)\{dN_i(u) - d\Lambda_0(u)Y_i(u)\Bigg] + o_p(1), \\ = n^{-1/2}\sum_{i=1}^{n}&\mathfrak{T}_i^j + o_p(1), \end{aligned}$$

which shows that $n^{-1/2}\mathfrak{T}^j$ in (**??**) is asymptotically equivalent to $n^{-1/2}$ times a sum of iid terms, and thus so is $n^{-1/2}\boldsymbol{\mathfrak{T}} = n^{-1/2}(\mathfrak{T}^1, \ldots, \mathfrak{T}^{D-1})^T$. Accordingly, if we define $\boldsymbol{\mathfrak{T}}_i = (\mathfrak{T}_i^1, \ldots, \mathfrak{T}_i^{D-1})^T$, which are iid for $i = 1, \ldots, n$, so that $n^{-1/2}\boldsymbol{\mathfrak{T}} = n^{-1/2}\sum_{i=1}^n \boldsymbol{\mathfrak{T}}_i + o_p(1)$, it follows by standard asymptotic normal theory that the asymptotic covariance matrix of $n^{-1/2}\boldsymbol{\mathfrak{T}}$ can be approximated by $\boldsymbol{\Sigma} = n^{-1}\sum_{i=1}^n(\boldsymbol{\mathfrak{T}}_i\boldsymbol{\mathfrak{T}}_i^T)$. As in the main paper, because $q(u, d^j)$ and $d\Lambda_0(u)$ are not known, it is natural to substitute $\widehat{q}(u, d^j)$ for $q(u, d^j)$ and $d\widehat{\Lambda}_0(u)$ for $d\Lambda_0(u)$ to obtain $\widehat{\mathfrak{T}}_i^j$ and $\widehat{\boldsymbol{\mathfrak{T}}}_i = (\widehat{\mathfrak{T}}_i^1, \ldots, \widehat{\mathfrak{T}}_i^{D-1})^T$, say, and estimate $\boldsymbol{\Sigma}$ by $\widehat{\boldsymbol{\Sigma}} = n^{-1}\sum_{i=1}^n(\widehat{\boldsymbol{\mathfrak{T}}}_i\widehat{\boldsymbol{\mathfrak{T}}}_i^T)$. In fact, it is straightforward to observe that $n^{-1/2}\boldsymbol{\mathfrak{T}} = n^{-1/2}\widehat{\boldsymbol{\mathfrak{T}}} = n^{-1/2}\sum_{i=1}^n \widehat{\boldsymbol{\mathfrak{T}}}_i$, so the test statistic can be formed equivalently as $\mathbb{Z} = n^{-1}\widehat{\boldsymbol{\mathfrak{T}}}^T\widehat{\boldsymbol{\Sigma}}^-\widehat{\boldsymbol{\mathfrak{T}}}$.

Web Appendix D: Improving Efficiency

D.1. Increased efficiency using estimated randomization probabilities

In Web Appendix B, we discuss modeling and estimation (by ML) of the probabilities $\omega_k(\boldsymbol{h}_k, a_k)$, $k = 1, \ldots, K$. Assuming that suitable models $\omega_k(\boldsymbol{h}_k, a_k; \boldsymbol{\gamma}_k)$, $k = 1, \ldots, K$, have been posited following the considerations in that Web Appendix and that $\boldsymbol{\gamma} = (\boldsymbol{\gamma}_1^T, \ldots, \boldsymbol{\gamma}_K^T)^T$ is estimated via ML, we now discuss the implications of substituting estimated probabilities obtained from the fitted models for the known probabilities in (**??**) of the main paper and thus in $\boldsymbol{\mathfrak{T}}$ in forming the test statistic. Namely, as noted in the main paper, we argue that substituting estimated probabilities for known probabilities yields a more powerful test as long as the models are correctly specified, and we demonstrate that estimation of $\boldsymbol{\gamma}$ must be taken into appropriate account in deriving and estimating the asymptotic covariance matrix of $\boldsymbol{\mathfrak{T}}$ with which to form the test statistic. Thus, with data from a SMART, it is advantageous to estimate the known randomization probabilities and use the test statistic with this appropriate covariance matrix. In fact, the arguments show that, if there are components of the histories $\boldsymbol{H}_k$ that are associated with the outcome, it is possible to exploit

these associations to gain further efficiency and thus further increases in power, analogous to the familiar tactic of covariate adjustment.

Denote $\mathfrak{T}$ with elements as in (??) with the models $\omega_k(\boldsymbol{h}_k, a_k; \boldsymbol{\gamma}_k)$, $k = 1, \ldots, K$ substituted as $\mathfrak{T}(\boldsymbol{\gamma})$, and, as in Web Appendix B, let $\widehat{\boldsymbol{\gamma}}$ be the ML estimator for $\boldsymbol{\gamma}$. According to Theorem 9.1 of Tsiatis (2006), with correctly-specified models, substituting $\widehat{\boldsymbol{\gamma}}$ in these models is equivalent to projecting each of the $D-1$ components of $\mathfrak{T}$, which contain the known probabilities, onto the space spanned by the score vector associated with the likelihood under the models. Because $\mathfrak{T}(\widehat{\boldsymbol{\gamma}})$ involves a projection, the asymptotic covariance matrix of $\mathfrak{T}(\widehat{\boldsymbol{\gamma}})$ is no larger than that of $\mathfrak{T}$ with the known probabilities in the sense of nonnegative definiteness, which implies that estimating $\boldsymbol{\gamma}$ results in efficiency gains relative to using the known probabilities. Thus, a modified test statistic, $\mathbb{Z}(\widehat{\boldsymbol{\gamma}})$, say, that takes these features into account will yield a more powerful test than the test statistic $\mathbb{Z}$ using the known probabilities.

Letting $\boldsymbol{\Sigma}_\gamma$ be the asymptotic covariance matrix of $\mathfrak{T}(\widehat{\boldsymbol{\gamma}})$, we now present a heuristic argument to motivate our proposed approach to obtaining an estimator for $\boldsymbol{\Sigma}$, $\widehat{\boldsymbol{\Sigma}}_\gamma$, to be used to form the test statistic. We first provide a representation of the score vector associated with the ML estimator $\widehat{\boldsymbol{\gamma}}$ in generic models $\omega_k(\boldsymbol{h}_k, a_k; \boldsymbol{\gamma}_k)$, $k = 1, \ldots, K$; examples of score vectors in the specific models considered in Web Appendix B are in (B.2) and (B.4). Similar to the formulation in Web Appendix A, let $A(u)$ denote the treatment received at time $u \geqslant 0$; and let M denote the number of all possible treatment options that could be administered at u, including "do nothing" if u is not the time of a decision point. Let $H^-(u)$ denote the history of information at time u but not including the treatment received at time u. Let $\omega\{\ell, u, H^-(u)\} = P\{A(u) = \ell | H^-(u)\}$, the probability of being assigned treatment ℓ at time u given the history $H^-(u)$, $\ell = 1, \ldots, M$, where this probability is positive only for treatments ℓ that are feasible at time u given the history $H^-(u)$.

Consider a model for $\omega\{\ell, u, H^-(u)\}$ given by $\omega\{\ell, u, H^-(u); \boldsymbol{\gamma}\}$ indexed by the finite-

dimensional parameter $\boldsymbol{\gamma}$. Then the contribution to the likelihood for $\boldsymbol{\gamma}$ for an individual, under the assumptions of consistency, the SRA, and positivity in the main paper, is given by

$$\prod_{u \leqslant U} \prod_{\ell=1}^{M} \omega\{\ell, u, H^{-}(u); \boldsymbol{\gamma}\}^{I\{A(u)=\ell\}},$$

so that the contribution to the loglikelihood is

$$\int_0^U \sum_{\ell=1}^{M} I\{A(u)=\ell\} \log\big[\omega\{\ell, u, H^{-}(u); \boldsymbol{\gamma}\}\big].$$

Then the score vector is the first partial derivative of this expression, namely,

$$\boldsymbol{S}_{\gamma}(\boldsymbol{\gamma}) = \int_0^U \sum_{\ell=1}^{M} I\{A(u)=\ell\} \frac{\partial/\partial\boldsymbol{\gamma}\big[\omega\{\ell, u, H^{-}(u); \boldsymbol{\gamma}\}\big]}{\omega\{\ell, u, H^{-}(u); \boldsymbol{\gamma}\}}.$$

Because $\sum_{\ell=1}^{M} \omega\{\ell, u, H^{-}(u); \boldsymbol{\gamma}\} = 1$ for all $\boldsymbol{\gamma}$,

$$\sum_{\ell=1}^{M} \partial/\partial\boldsymbol{\gamma}\big[\omega\{\ell, u, H^{-}(u); \boldsymbol{\gamma}\}\big] = 0,$$

so that

$$\boldsymbol{S}_{\gamma}(\boldsymbol{\gamma}) = \int_0^U \sum_{\ell=1}^{M} \big[I\{A(u)=\ell\} - \omega\{\ell, u, H^{-}(u); \boldsymbol{\gamma}\}\big] \frac{\partial/\partial\boldsymbol{\gamma}\big[\omega\{\ell, u, H^{-}(u); \boldsymbol{\gamma}\}\big]}{\omega\{\ell, u, H^{-}(u); \boldsymbol{\gamma}\}}. \tag{D.1}$$

Aligned with Tsiatis (2006, Theorem 9.1), consider the space of "augmentation terms," denoted by Λ_2, defined as all functions of the observed data, $g(\mathcal{O})$, say, such that

$$E\{g(\mathcal{O}) \,|\, \mathcal{W}^{*}\} = 0.$$

Motivated by the space spanned by score vectors with respect to $\boldsymbol{\gamma}$ (D.1), define the subspace Λ_{Aug} of Λ_2 by the space of functions of the form

$$\int_0^U \sum_{\ell=1}^{M} \big[I\{A(u)=\ell\} - \omega\{\ell, u, H^{-}(u); \boldsymbol{\gamma}_0\}\big]\, b_{\ell}\{u, H^{-}(u)\}, \tag{D.2}$$

where $\boldsymbol{\gamma}_0$ is the true value of $\boldsymbol{\gamma}$ so that $\omega\{\ell, u, H^{-}(u); \boldsymbol{\gamma}_0\}$ = the true $\omega\{\ell, u, H^{-}(u)\}$; and $b_{\ell}\{u, H^{-}(u)\}$ are arbitrary functions. That any element of Λ_{Aug} is contained in Λ_2 follows

because

$$
\begin{aligned}
&E\Big(\big[I\{A(u)=\ell\}-\omega\{\ell,u,H^-(u);\boldsymbol{\gamma}_0\}\big]\, b_\ell\{u,H^-(u)\}|\mathcal{W}^*\Big)\\
&\quad = E\left\{E\Big(\big[I\{A(u)=\ell\}-\omega\{\ell,u,H^-(u);\boldsymbol{\gamma}_0\}\big]\, b_\ell\{u,H^-(u)\}|H^-(u),\mathcal{W}^*\Big)\Big|\,\mathcal{W}^*\right\}\\
&\quad = 0,
\end{aligned}
$$

using the fact that $E\Big(\big[I\{A(u)=\ell\}|H^-(u),\mathcal{W}^*\Big) = \omega\{\ell,u,H^-(u);\boldsymbol{\gamma}_0\}$. Clearly, from (D.1), the space $\Lambda_{\rm Aug}$ contains the space spanned by $\boldsymbol{S}_\gamma(\boldsymbol{\gamma})$. Thus, by considering test statistics formed by projecting each of the $D-1$ components of $\boldsymbol{\mathfrak{T}}$ onto the space $\Lambda_{\rm Aug}$, we can obtain tests that are more powerful than those based on $\mathbb{Z}$ using known probabilities. These developments suggest the approach presented in the main paper for constructing the test statistic. Namely, instead of considering the entire space $\Lambda_{\rm Aug}$, we consider projecting onto a subspace spanned by a finite number of elements of $\Lambda_{\rm Aug}$, which are of the form in (D.2) with the elements of $\Lambda_{\rm Aug}$ determined by analyst-specified choices for the functions of the history $b_\ell\{u,H^-(u)\}$; refer to these functions as basis functions. As long as this subspace contains all the elements of $\boldsymbol{S}_\gamma(\boldsymbol{\gamma})$, so that the chosen basis functions include the components of $\boldsymbol{S}_\gamma(\boldsymbol{\gamma})$, the efficiency gain associated with estimation of $\boldsymbol{\gamma}$ will be taken into account.

Based on these results, operationally, to obtain $\widehat{\boldsymbol{\Sigma}}_\gamma$ with which to form a test statistic, for each $j=1,\ldots,D$, proceed as follows. Denote by $\widehat{\mathfrak{T}}_i^j(\widehat{\boldsymbol{\gamma}})$ $\widehat{\mathfrak{T}}_i^j$ defined in the main paper (i.e., as in (**??**) with $\widehat{q}(u,d^j)$ and $d\widehat{\Lambda}_0(u)$ substituted) for subject i, $i=1,\ldots,n$, with the estimated probabilities substituted. Denote by $\boldsymbol{S}_{\gamma,i}(\widehat{\boldsymbol{\gamma}})$ the score vector associated with the likelihood for $\boldsymbol{\gamma}$ for subject i in the chosen models $\omega_k(\boldsymbol{h}_k,a_k;\boldsymbol{\gamma}_k)$, $k=1,\ldots,K$, with the estimated probabilities substituted. Then for each j carry out a linear regression of the $\widehat{\mathfrak{T}}_i^j(\widehat{\boldsymbol{\gamma}})$ on $\boldsymbol{S}_{\gamma,i}(\widehat{\boldsymbol{\gamma}})$ and form for each subject $i=1,\ldots,n$ the residuals from this fit, $\widehat{\mathfrak{T}}_i^{j,R}(\widehat{\boldsymbol{\gamma}})$, say. Then, defining $\widehat{\boldsymbol{\mathfrak{T}}}_i^R(\widehat{\boldsymbol{\gamma}}) = \{\widehat{\mathfrak{T}}_i^{1,R}(\widehat{\boldsymbol{\gamma}}),\ldots,\widehat{\mathfrak{T}}_i^{D-1,R}(\widehat{\boldsymbol{\gamma}})\}^T$, define

$$
\widehat{\boldsymbol{\Sigma}}_\gamma = n^{-1}\sum_{i=1}^n\{\widehat{\boldsymbol{\mathfrak{T}}}_i^R(\widehat{\boldsymbol{\gamma}})\widehat{\boldsymbol{\mathfrak{T}}}_i^R(\widehat{\boldsymbol{\gamma}})^T\}. \tag{D.3}
$$

Letting $\widehat{\mathfrak{T}}^R(\widehat{\gamma}) = \sum_{i=1}^n \widehat{\mathfrak{T}}_i^R(\widehat{\gamma})$, form the test statistic as

$$\mathbb{Z}(\widehat{\gamma}) = n^{-1}\widehat{\mathfrak{T}}^R(\widehat{\gamma})^T \widehat{\boldsymbol{\Sigma}}_\gamma^- \widehat{\mathfrak{T}}^R(\widehat{\gamma}) \tag{D.4}$$

As a demonstration, consider Example 1 in Web Appendix B of a two-stage SMART. Here, the randomization probabilities are known, but the foregoing discussion indicates that a more powerful test can be obtained by estimating the randomization probabilities using sample proportions. In this case, to obtain the test statistic, for each $j = 1, \ldots, 8$, perform a linear regression with dependent variable $\widehat{\mathfrak{T}}_i^j(\widehat{\gamma})$ and design matrix with ith row, from (B.2),

$$\begin{aligned}\big[\{I(A_{1i}=1) - \text{expit}(\widehat{\gamma}_1)\}, I(A_{1i}=0, R_{2i}=1, \kappa_i=2)\{I(A_{2i}=2) - \text{expit}(\widehat{\gamma}_{21})\},\\ I(A_{1i}=0, R_{2i}=0, \kappa_i=2)\{I(A_{2i}=2) - \text{expit}(\widehat{\gamma}_{22})\},\\ I(A_{1i}=1, R_{2i}=1, \kappa_i=2)\{I(A_{2i}=2) - \text{expit}(\widehat{\gamma}_{23})\},\\ I(A_{1i}=1, R_{2i}=0, \kappa_i=2)\{I(A_{2i}=3) - \text{expit}(\widehat{\gamma}_{24})\}\big],\end{aligned} \tag{D.5}$$

and obtain the residuals $\widehat{\mathfrak{T}}_i^{j,R}(\widehat{\gamma})$ from this fit and then $\widehat{\boldsymbol{\Sigma}}_\gamma$ as in (D.3) and the test statistic as in (D.4).

Similarly, in Example 2 in Web Appendix B of a two-stage observational study, where of necessity $\omega_k(\boldsymbol{h}_k, a_k)$, $k = 1, 2$, must be modeled and fitted, the test statistic would be found by, for each $j = 1, \ldots, 4$, by linear regression with dependent variable $\widehat{\mathfrak{T}}_i^j(\widehat{\gamma})$ and design matrix with ith row, from (B.4),

$$\begin{aligned}\big[\{I(A_{1i}=1) - \text{expit}(\widehat{\boldsymbol{\gamma}}_1^T \widetilde{\boldsymbol{H}}_{1i})\}\widetilde{\boldsymbol{H}}_{1i}^T, I(A_{1i}=0, R_{2i}=0, \kappa_i=2)\{I(A_{2i}=1) - \text{expit}(\widehat{\boldsymbol{\gamma}}_{21}^T \widetilde{\boldsymbol{H}}_{2i})\}\widetilde{\boldsymbol{H}}_{2i}^T,\\ I(A_{1i}=1, R_{2i}=0, \kappa_i=2)\{I(A_{2i}=1) - \text{expit}(\widehat{\boldsymbol{\gamma}}_{22}^T \widetilde{\boldsymbol{H}}_{2i})\}\widetilde{\boldsymbol{H}}_{2i}^T\big].\end{aligned}$$

Then obtain the residuals $\widehat{\mathfrak{T}}_i^{j,R}(\widehat{\gamma})$ from this regression and proceed as above.

D.2. Remarks on Li et al. (2014)

It is straightforward to demonstrate that the approach of Li et al. (2014) can be viewed as a special case of our general testing approach, although formulated explicitly and evaluated

in their article only for a situation analogous to that of Example 2 in Web Appendix B. Thus, the foregoing developments contradict the claim of Li et al. (2014) that "estimation of these (treatment selection) probabilities does not have an effect on the asymptotic distribution of the weighted logrank statistic." In this example and in general, the above arguments demonstrate that there is an effect of estimation of the probabilities (i.e., estimation of $\boldsymbol{\gamma}$ here). The proof of Theorem 1 in the Appendix of Li et al. (2014) that there is no effect of estimating the probabilities hinges on the equality in their equation (5). If (5) is true, it implies that their quantity A_n in their equation (2) converges in probability to zero and thus that there is no dependence of the large-sample distribution of the quantity on the left hand side of their (2) and thus their test statistic on estimation of their parameter θ ($\boldsymbol{\gamma}$ here). However, we now argue that the equality in (5) does not hold; we provide a heuristic sketch of the argument. Using roughly their notation and letting Z denote the data, the expectation on the left hand side of their equation (5) is of the form

$$E_\theta\left[\frac{\partial}{\partial\theta}\{W(Z;\theta)\}\left\{\int_0^\tau \xi(u)dM(u)\right\}\right], \tag{D.6}$$

where $W(Z;\theta)$ and $dM(u)$ are functions of Z and $\xi(u)$ is a deterministic function, and E_θ indicates expectation with respect to the distribution of the data evaluated at θ. The distribution of the data depends on θ, as the data involve treatment assignment indicators, so that, letting $p(z;\theta)$ denote the density of Z, (D.6) is

$$\int \frac{\partial}{\partial\theta}\{W(z;\theta)\}\left\{\int_0^\tau \xi(u)dM(u)\right\}p(z;\theta)\,dz. \tag{D.7}$$

However, the right hand side of their (5) is, interchanging expectation and integration,

$$\begin{aligned}\frac{\partial}{\partial\theta}E_\theta\left[W(Z;\theta)\left\{\int_0^\tau \xi(u)dM(u)\right\}\right] &= \frac{\partial}{\partial\theta}\left[\int W(z;\theta)\left\{\int_0^\tau \xi(u)dM(u)\right\}p(z;\theta)\,dz\right]\\ &= \int \frac{\partial}{\partial\theta}\{W(z;\theta)\}\left\{\int_0^\tau \xi(u)dM(u)\right\}p(z;\theta)\,dz + \int W(z;\theta)\left\{\int_0^\tau \xi(u)dM(u)\right\}S_\theta(z;\theta)p(z;\theta)\,dz,\end{aligned} \tag{D.8}$$

where $S_\theta(z;\theta) = \partial/\partial\theta\{p(z;\theta)\}/p(z;\theta)$ is the score associated with ML estimation of θ.

Accordingly, their equation (5) does not hold as shown; rather, from (D.7) and (D.8),

$$E_\theta\left[\frac{\partial}{\partial\theta}\{W(Z;\theta)\}\left\{\int_0^\tau \xi(u)dM(u)\right\}\right]$$
$$= \frac{\partial}{\partial\theta}E_\theta\left[W(Z;\theta)\left\{\int_0^\tau \xi(u)dM(u)\right\}\right] - E_\theta\left[W(z;\theta)\left\{\int_0^\tau \xi(u)dM(u)\right\}S_\theta(z;\theta)\right]. \quad \text{(D.9)}$$

Equation (4) of Li et al. (2014) implies that the first term in (D.9) is equal to zero; thus, their equation (5) should be

$$E_\theta\left[\frac{\partial}{\partial\theta}\{W(Z;\theta)\}\left\{\int_0^\tau \xi(u)dM(u)\right\}\right] = -E_\theta\left[W(z;\theta)\left\{\int_0^\tau \xi(u)dM(u)\right\}S_\theta(z;\theta)\right] \neq 0,$$

so that A_n in their (2) converges in probability to the right hand side of this expression rather than to zero. The implication is that there is in fact an effect of estimation of their parameter θ indexing the models for the treatment probabilities (our $\boldsymbol{\gamma}$), which depends on the score vector, analogous to our results.

In fact, because as above estimation of $\boldsymbol{\gamma}$ leads to an increase in efficiency and because Li et al. (2014) do not take into account the effect of estimating $\boldsymbol{\gamma}$ in the covariance matrix used to form their test statistic, the covariance matrix they use overstates the uncertainty in the components of $\boldsymbol{\mathfrak{T}}$. Consequently, under H_0, their proposed test is expected to be conservative under H_0. This behavior is evident in their Table 1, which shows that, even with sample sizes as large as $n = 10,000$, the empirical type I error rate is lower than the nominal 0.05 level of significance.

D.3. Gaining additional efficiency using covariates

Returning to our approach, the above arguments and form of the space Λ_{Aug} suggest that, if there are components of the history at any decision point that are associated with the outcome, it may be possible to gain further efficiency by exploiting these associations. In particular, consider a SMART. In addition to including as basis functions the elements of the score vector associated with estimators of the randomization probabilities as above, one

can also include additional basis functions that are functions of these components of the history. For example, in the SMART in Example 1, in addition to having columns that are determined by elements of (B.2) for each subject i as in (D.5), one could add further columns to the design matrix by appending to the ith row the additional components

$$\begin{aligned}\big[\{I(A_{1i}=1)-\text{expit}(\widehat{\gamma}_1)\}\widetilde{\boldsymbol{H}}_{1i}^T, I(A_{1i}=0,R_{2i}=1,\kappa_i=2)\{I(A_{2i}=2)-\text{expit}(\widehat{\gamma}_{21})\}\widetilde{\boldsymbol{H}}_{21i,i}^T,\\ I(A_{1i}=0,R_{2i}=0,\kappa_i=2)\{I(A_{2i}=2)-\text{expit}(\widehat{\gamma}_{22})\}\widetilde{\boldsymbol{H}}_{22,i}^T,\\ I(A_{1i}=1,R_{2i}=1,\kappa_i=2)\{I(A_{2i}=2)-\text{expit}(\widehat{\gamma}_{23})\}\widetilde{\boldsymbol{H}}_{23,i}^T,\\ I(A_{1i}=1,R_{2i}=0,\kappa_i=2)\{I(A_{2i}=3)-\text{expit}(\widehat{\gamma}_{24})\}\widetilde{\boldsymbol{H}}_{24,i}^T\big],\end{aligned} \tag{D.10}$$

where now $\widetilde{\boldsymbol{H}}_1$ and $\widetilde{\boldsymbol{H}}_{2\ell}$, $\ell=1,\ldots,4$, are vectors of basis functions of $\boldsymbol{H}_1$ and $\boldsymbol{H}_2$, but with no "1" in the first position. For example, if $\boldsymbol{H}_1$ contains two baseline covariates X_{11}, X_{12}, and $\boldsymbol{H}_2$ contains one covariate X_2 ascertained between Decisions 1 and 2, one could take $\widetilde{\boldsymbol{H}}_1=(X_{11},X_{12})^T$ and $\widetilde{\boldsymbol{H}}_{2\ell}=(X_{11},X_{12},X_2)^T$, $\ell=1,\ldots,4$. In this case, the design matrix would be of dimension $(n\times 19)$. Of course, the analyst must be judicious in specifying these vectors of basis functions, as including in them information in the history for which the association is not strong could lead to finite-sample "noise" that diminishes or offsets efficiency gains in finite samples. To obtain the test statistic, one would proceed as above and obtain the residuals $\widehat{\mathfrak{T}}_i^{j,R}(\widehat{\boldsymbol{\gamma}})$ from the regression using this design matrix, compute $\widehat{\boldsymbol{\Sigma}}_\gamma$ as in (D.3), and form the test statistic as in (D.4).

D.4. Remarks on Kidwell and Wahed (2013)

We provide an argument demonstrating the contention of Section **??** of the main paper that the components of the vector need not be martingales with respect to the filtration given by Kidwell and Wahed (2013). Because the test of Kidwell and Wahed (2013) is applicable for $K=2$ and the design in Figure **??** of the main paper without the control regime, and their

formulation does not involve covariates, we consider this setting with no available covariate information for simplicity. In this case, the history up to time $u \geqslant 0$ is given by (recall $\mathcal{T}_1 = 0$ is superfluous, so we eliminate it from the history for the purpose of this argument)

$$H(u) = \{A_1, I(\kappa \geqslant 2, \mathcal{T}_2 \leqslant u), I(\kappa \geqslant 2, \mathcal{T}_2 \leqslant u)(\mathcal{T}_2, A_2), I(U < u), (U, \Delta)I(U < u)\}$$

is the filtration of Kidwell and Wahed (2013) in the current notation.

Under the null hypothesis H_0 in (**??**) of the main paper, the hazard rates $\lambda(u, d^j)$, $j = 1, \ldots, D = 4$ are all equal to the common hazard rate $\lambda_0(u)$. In computing the covariance matrix of the numerator of their test statistic under H_0, Kidwell and Wahed (2013) invoke the theory of counting process martingales. Specifically, the authors assume that the increment $dM(u) = dN(u) - d\Lambda_0(u)Y(u)$, where as in the main paper $N(u) = I(U \leqslant u, \Delta = 1)$ and $Y(u) = I(U \geqslant u)$, is a $H(u^-)$ martingale increment; i.e.,

$$E\{dM(u) \mid H(u^-)\} = 0.$$

Consequently, for each regime j,

$$E\{\Omega(u, d^j) dM(u) \mid H(u^-)\} = \Omega(u, d^j) E\{dM(u) \mid H(u^-)\} = 0.$$

However, in general, the intensity process for the counting process $N(u)$ is

$$\begin{aligned} E\{dN(u) \mid H(u^-)\} = \big[&d\Lambda_{NR}(u, A_1)\{1 - I(\kappa \geqslant 2, \mathcal{T}_2 \leqslant u)\} \\ &+ d\Lambda_R(u, A_1, \mathcal{T}_2, A_2) I(\kappa \geqslant 2, \mathcal{T}_2 \leqslant u)\}\big] Y(t). \end{aligned}$$

Here, $d\Lambda_{NR}(u, A_1)$ is the hazard of experiencing the event at time u for an individual who is at risk at time u and has not yet had a response, which may be a function of u and A_1; and $d\Lambda_R(u, A_1, \mathcal{T}_2, A_2)$ is the hazard of experiencing the event at time u for an individual who is at risk at time u, has achieved a response at time $\mathcal{T}_2 < u$, and received treatments A_1 and A_2 at Decisions 1 and 2, which may be a function of u, A_1, $\mathcal{T}_2$, and A_2.

In the data generative scenarios of Kidwell and Wahed (2013) (our Scenarios 1 and 2, for example) and our Scenarios 3-5, under the null hypothesis, $d\Lambda_{NR}(u, A_1) = d\Lambda_{NR}(u)$, so does

not depend on A_1; similarly, $d\Lambda_R(u, A_1, \mathcal{T}_2, A_2) = d\Lambda_R(u)$, so does not depend on $A_1, \mathcal{T}_2, A_2$. However, $d\Lambda_{NR}(u)$ need not be equal to $d\Lambda_R(u)$. Thus, under these generative scenarios,

$$E\{dN(u) \mid H(u^-)\} = \big[d\Lambda_{NR}(u)\{1 - I(\kappa \geqslant 2, \mathcal{T}_2 \leqslant u)\} \\ + d\Lambda_R(u) I(\kappa \geqslant 2, \mathcal{T}_2 \leqslant u)\}\big] Y(u),$$

in which case

$$E\{dM(u) \mid H(u^-)\} = \big[d\Lambda_{NR}(u)\{1 - I(\kappa \geqslant 2, \mathcal{T}_2 \leqslant u)\} \\ + d\Lambda_R(u) I(\kappa \geqslant 2, \mathcal{T}_2 \leqslant u) - d\Lambda_0(u)\}\big] Y(u). \tag{D.11}$$

(D.11) is not equal to zero unless $d\Lambda_{NR}(u) = d\Lambda_R(u)$, in which case

$$d\Lambda_{NR}(u) = d\Lambda_R(u) = d\Lambda_0(u),$$

and (D.11) equals zero. From Web Appendix A, $E\{\Omega(u, d^j) dM(u)\} = 0$, which is the basis of our approach; however, as the above demonstrates, $E\{\Omega(u, d^j) dM(u) \mid H(u^-)\}$ does not necessarily equal to zero unless $d\Lambda_{NR}(u) = d\Lambda_R(u)$.

The fact that $dM(u)$ is not necessarily a $H(u^-)$ martingale increment does not affect the unbiasedness of the vector of paired comparisons on which the test statistic of Kidwell and Wahed (2013) is based, but may impact the relevance of the authors' derivation of the covariance matrix of this vector, which depends on the assumed martingale structure. As we discuss further in Web Appendix F, this feature may underlie the simulation results reported in Section **??** of the main paper.

Web Appendix E: Second Order Correction to $\widehat{\boldsymbol{\Sigma}}$

As discussed in Section **??** of the main paper, in small samples, the proposed test may be anticonservative. This feature is a consequence of the fact that the estimator $\widehat{\boldsymbol{\Sigma}}$ for the asymptotic covariance matrix $\boldsymbol{\Sigma}$ of $\boldsymbol{\mathfrak{T}}$ understates the uncertainty in the components of $\boldsymbol{\mathfrak{T}}$ in finite samples when n is not sufficiently large. Such behavior is not uncommon with methods based on first-order semiparametric theory.

Accordingly, to improve the finite-sample performance of the test so that it achieves the nominal level of significance, we propose a second-order correction to the estimator for $\boldsymbol{\Sigma}$ in the spirit of similar corrections in other contexts (e.g. Kauermann and Carroll, 2001; Schaubel, 2005; Wang et al., 2023). Because we have observed anticonservatism of the test in simulations of SMARTs with small sample sizes in which the randomization probabilities were taken to be known or estimated, for simplicity, we present the argument leading to the corrected estimator for $\boldsymbol{\Sigma}$ in the case that the randomization probabilities are known. The same correction tactic can be used with estimated randomization probabilities and with inclusion of additional covariates to gain efficiency, as demonstrated in Web Appendix D.

We present a heuristic argument leading to the proposed correction. Recall from the main paper that

$$d\widehat{\Lambda}_0(u) = \frac{\sum_{i=1}^n \sum_{j=1}^D \Omega(u,d^j) dN_i(u)}{\sum_{i=1}^n \overline{Y}_i(u)},$$

$$\widehat{q}(u,d^j) = \frac{\sum_{i=1}^n \Omega_i(u,d^j) Y_i(u)}{\sum_{i=1}^n \overline{Y}_i(u)}$$

where $\overline{Y}_i(u) = \sum_{j=1}^D \Omega_i(u,d^j) Y_i(u)$. As in the main paper, let $q(u,d^j)$ be the limit in probability of $\widehat{q}(u,d^j)$. For brevity, define

$$dM_i(u,\Lambda_0) = dN_i(u) - d\Lambda_0(u) Y_i(u).$$

Then, from (**??**) of the main paper, write $\mathfrak{T}_i^j$ as

$$\Psi_i(d^j;q,\Lambda_0) = \int_0^\infty \left\{ \Omega_i(u,d^j) - q(u,d^j) \sum_{j'=1}^D \Omega_i(u,d^{j'}) \right\} dM_i(u,\Lambda_0), \qquad \text{(E.1)}$$

and let

$$\boldsymbol{\Psi}_i(q,\Lambda_0) = \{\Psi_i(d^1;q,\Lambda_0),\ldots,\Psi_i(d^{D-1};q,\Lambda_0)\}^T,$$

which is the same as $\boldsymbol{\mathfrak{T}}_i$ in the main paper, so that the asymptotic covariance matrix of $n^{-1/2}\boldsymbol{\mathfrak{T}} = n^{-1/2}\sum_{i=1}^n \boldsymbol{\mathfrak{T}}_i = n^{-1/2}\sum_{i=1}^n \boldsymbol{\Psi}_i(q,\Lambda_0)$ can be approximated to first order by

$$n^{-1}\boldsymbol{\Sigma} = n^{-1}\sum_{i=1}^n \{\boldsymbol{\Psi}_i(q,\Lambda_0)\boldsymbol{\Psi}_i(q,\Lambda_0)^T\}. \qquad \text{(E.2)}$$

In the main paper, we propose estimating (E.2) by

$$n^{-1}\sum_{i=1}^{n}\{\boldsymbol{\Psi}_i(\widehat{q},\widehat{\Lambda}_0)\boldsymbol{\Psi}_i(\widehat{q},\widehat{\Lambda}_0)^T\}. \tag{E.3}$$

To identify the source of the finite-sample bias and to correct it, write

$$\boldsymbol{\Psi}_i(\widehat{q},\widehat{\Lambda}_0)=\boldsymbol{\Psi}_i(q,\Lambda_0)-\{\boldsymbol{\Psi}_i(q,\Lambda_0)-\boldsymbol{\Psi}_i(q,\widehat{\Lambda}_0)\}-\{\boldsymbol{\Psi}_i(q,\widehat{\Lambda}_0)-\boldsymbol{\Psi}_i(\widehat{q},\widehat{\Lambda}_0)\},$$

so that the estimator (E.3) can be written as

$$\begin{aligned}
n^{-1}\sum_{i=1}^{n}\{\boldsymbol{\Psi}_i(\widehat{q},\widehat{\Lambda}_0)\boldsymbol{\Psi}_i(\widehat{q},\widehat{\Lambda}_0)^T\} &= n^{-1}\sum_{i=1}^{n}\{\boldsymbol{\Psi}_i(q,\Lambda_0)\boldsymbol{\Psi}_i(q,\Lambda_0)^T\}\\
&\quad - n^{-1}\sum_{i=1}^{n}\Big[\boldsymbol{\Psi}_i(q,\Lambda_0)\{\boldsymbol{\Psi}_i(q,\Lambda_0)-\boldsymbol{\Psi}_i(q,\widehat{\Lambda}_0)\}^T\Big] &\text{(E.4)}\\
&\quad - n^{-1}\sum_{i=1}^{n}\Big[\boldsymbol{\Psi}_i(q,\Lambda_0)\{\boldsymbol{\Psi}_i(q,\Lambda_0)-\boldsymbol{\Psi}_i(q,\widehat{\Lambda}_0)\}^T\Big]^T\\
&\quad - n^{-1}\sum_{i=1}^{n}\Big[\boldsymbol{\Psi}_i(q,\Lambda_0)\{\boldsymbol{\Psi}_i(q,\widehat{\Lambda}_0)-\boldsymbol{\Psi}_i(\widehat{q},\widehat{\Lambda}_0)\}^T\Big] &\text{(E.5)}\\
&\quad - n^{-1}\sum_{i=1}^{n}\Big[\boldsymbol{\Psi}_i(q,\Lambda_0)\{\boldsymbol{\Psi}_i(q,\widehat{\Lambda}_0)-\boldsymbol{\Psi}_i(\widehat{q},\widehat{\Lambda}_0)\}^T\Big]^T\\
&\quad + \text{smaller order terms}, &\text{(E.6)}
\end{aligned}$$

where we disregard the terms in (E.6) as they involve sample averages of the product of the differences $\{\boldsymbol{\Psi}_i(q,\Lambda_0)-\boldsymbol{\Psi}_i(q,\widehat{\Lambda}_0)\}$ and $\{\boldsymbol{\Psi}_i(q,\widehat{\Lambda}_0)-\boldsymbol{\Psi}_i(\widehat{q},\widehat{\Lambda}_0)\}$.

First consider (E.4) and define for brevity

$$A_i(u,q,d^j)=\left\{\Omega_i(u,d^j)-q(u,d^j)\sum_{j'=1}^{D}\Omega_i(u,d^{j'})\right\},$$

and consider a typical term in the matrix in a summand of (E.4). I.e., for regimes d^j and $d^{j'}$, consider

$$\Psi_i(d^j;q,\Lambda_0)\{\Psi_i(d^{j'};q,\Lambda_0)-\Psi_i(d^{j'};q,\widehat{\Lambda}_0)\},$$

which by (E.1) is equal to

$$\int_0^\infty A_i(u,q,d^j)dM_i(u,\Lambda_0)\int_0^\infty A_i(u,q,d^{j'})\{d\widehat{\Lambda}_0(u)-d\Lambda_0(u)\}Y_i(u). \tag{E.7}$$

But

$$\{d\widehat{\Lambda}_0(u) - d\Lambda_0(u)\} = \frac{n^{-1}\sum_{\ell=1}^n \sum_{j'=1}^D \Omega_\ell(u, d^{j'})dM_\ell(u,\Lambda_0)}{n^{-1}\sum_{\ell=1}^n \overline{Y}_\ell(u)} \tag{E.8}$$

Note that the denominator of (E.8), $n^{-1}\sum_{\ell=1}^n \overline{Y}_\ell(u)$, will converge in probability as $n \to \infty$, so we can regard this quantity as fixed in subsequent arguments, as presumably the difference between it and its limit in probability will be a second order effect.

Thus, interchanging sums, (E.7) is equal to

$$n^{-1}\sum_{\ell=1}^n \frac{\left\{\int_0^\infty A_i(u,d^j)dM_i(u,\Lambda_0)\ \int_0^\infty A_i(u,q,d^{j'})\sum_{j'=1}^D \Omega_\ell(u,d^{j'})dM_\ell(u,\Lambda_0)\right\} Y_i(u)}{n^{-1}\sum_{\ell=1}^n \overline{Y}_\ell(u)}. \tag{E.9}$$

The expectation of a summand in (E.9) when $\ell \neq i$ is zero; thus, the expectation of (E.9) is the same as the expectation of

$$\left\{\int_0^\infty A_i(u,q,d^j)dM_i(u,\Lambda_0)\right\} \frac{\left\{\int_0^\infty A_i(u,q,d^j)\sum_{j'=1}^D \Omega_i(u,d^{j'})dM_i(u,\Lambda_0)\right\}}{n^{-1}\sum_{\ell=1}^n \overline{Y}_\ell(u)},$$

where we have used the fact that $dM_i(u,\Lambda_0)Y_i(u) = dM_i(u,\Lambda_0)$.

Based on these developments, letting $\boldsymbol{A}_i(u,q) = \{A_i(u,q,d^1),\dots,A_i(u,q,d^{D-1})\}^T$, we propose estimating (E.4) by

$$n^{-2}\sum_{i=1}^n \left\{\int_0^\infty \boldsymbol{A}_i(u)dM_i(u,\Lambda_0)\right\} \frac{\left\{\int_0^\infty \boldsymbol{A}_i(u,q)^T \sum_{j'=1}^D \Omega_i(u,d^{j'})dM_i(u,\Lambda_0)\right\}}{n^{-1}\sum_{\ell=1}^n \overline{Y}_\ell(u)},$$

where in practice we replace $q(u,d^j)$ and $\Lambda_0(u)$ by the estimators $\widehat{q}(u,d^)$ and $\widehat{\Lambda}_0(u)$. Defining further

$$\boldsymbol{G}_i(q,\Lambda_0) = \frac{\left\{\int_0^\infty \boldsymbol{A}_i(u,q)\sum_{j'=1}^D \Omega_i(u,d^{j'})dM_i(u,\Lambda_0)\right\}}{n^{-1}\sum_{\ell=1}^n \overline{Y}_\ell(u)},$$

and noting that

$$\int_0^\infty \boldsymbol{A}_i(u,q)dM_i(u,\Lambda_0) = \boldsymbol{\Psi}_i(q,\Lambda_0),$$

we estimate (E.4) by

$$n^{-2}\sum_{i=1}^n \boldsymbol{\Psi}_i(q,\Lambda_0)\boldsymbol{G}_i(q,\Lambda_0)^T,$$

where in practice we replace $q(u,d^j)$ and $\Lambda_0(u)$ by the estimators $\widehat{q}(u,d^j)$ and $\widehat{\Lambda}_0(u)$.

A similar argument can be used to derive an estimator for (E.5). First note that (E.5) is

equal to

$$n^{-1}\sum_{i=1}^{n}\left[\boldsymbol{\Psi}_i(q,\Lambda_0)\{\boldsymbol{\Psi}_i(q,\Lambda_0)-\boldsymbol{\Psi}_i(\widehat{q},\Lambda_0)\}^T\right]+\text{smaller order terms.}$$

Thus, consider a typical term in the matrix in a summand; i.e., for regimes d^j and $d^{j'}$,

$$\Psi_i(d^j;q,\Lambda_0)\{\Psi_i(d^{j'};q,\Lambda_0)-\Psi_i(d^{j'};\widehat{q},\Lambda_0)\},$$

which is equal to

$$\left\{\int_0^\infty A_i(u,q,d^j)dM_i(u,\Lambda_0)\right\}\int_0^\infty\{\widehat{q}(u,d^{j'})-q(u,d^{j'})\}\sum_{j'=1}^{D}\Omega_i(u,d^{j'})dM_i(u,\Lambda_0). \tag{E.10}$$

It is straightforward to show that $\{\widehat{q}(u,d^{j'})-q(u,d^{j'})\}$ is equal to

$$n^{-1}\sum_{\ell=1}^{n}\frac{\Omega_\ell(u,d^{j'})Y_\ell(u)-q(u,d^{j'})\sum_{j''=1}^{D}\Omega_\ell(u,d^{j''})Y_\ell(u)}{n^{-1}\sum_{\ell=1}^{n}\overline{Y}_\ell(u)}.$$

Thus, substituting, (E.10) equals

$$n^{-1}\sum_{\ell=1}^{n}\left\{\int_0^\infty A_i(u,q,d^j)dM_i(u,\Lambda_0)\right\}\frac{\left\{\Omega_\ell(u,d^{j'})Y_\ell(u)-q(u,d^{j'})\sum_{j''=1}^{D}\Omega_\ell(u,d^{j''})Y_\ell(u)\right\}}{n^{-1}\sum_{\ell=1}^{n}\overline{Y}_\ell(u)}$$
$$\times\sum_{j''=1}^{D}\Omega_i(u,d^{j''})dM_i(u,\Lambda_0). \tag{E.11}$$

As above, when $\ell\neq i$, the expectation of a summand in (E.11) is zero. Thus, using $dM_i(u,\Lambda_0)Y_i(u)=dM_i(u,\Lambda_0)$, we replace (E.11) by

$$\left\{\int_0^\infty A_i(u,q,d^j)dM_i(u,\Lambda_0)\right\}\frac{\Omega_i(u,d^{j'})-q(u,d^{j'})\sum_{j''=1}^{D}\Omega_i(u,d^{j''})}{n^{-1}\sum_{\ell=1}^{n}\overline{Y}_\ell(u)}\sum_{j''=1}^{D}\Omega_i(u,d^{j''})dM_i(u,\Lambda_0).$$

Then, using the definitions of $\boldsymbol{A}_i(u,q)$ and $\boldsymbol{G}_i(q,\Lambda_0)$ above, we estimate (E.5) by

$$n^{-2}\sum_{i=1}^{n}\boldsymbol{\Psi}_i(q,\Lambda_0)\boldsymbol{G}_i(q,\Lambda_0)^T,$$

where in practice we replace $q(u,d^j)$ and $\Lambda_0(u)$ by the estimators $\widehat{q}(u,d^j$ and $\widehat{\Lambda}_0(u)$.

Collecting these results, we propose the bias-corrected estimator for $\boldsymbol{\Sigma}$ given by

$$n^{-1}\sum_{i=1}^{n}\{\boldsymbol{\Psi}_i(\widehat{q},\widehat{\Lambda}_0)\boldsymbol{\Psi}_i(\widehat{q},\widehat{\Lambda}_0)^T\}+n^{-2}\sum_{i=1}^{n}\left\{2\boldsymbol{\Psi}_i(\widehat{q},\widehat{\Lambda}_0)\boldsymbol{G}_i(\widehat{q},\widehat{\Lambda}_0)^T+2\boldsymbol{G}_i(\widehat{q},\widehat{\Lambda}_0)\boldsymbol{\Psi}_i(\widehat{q},\widehat{\Lambda}_0)^T\right\}$$
$$=\widehat{\boldsymbol{\Sigma}}+n^{-1}\left[n^{-1}\sum_{i=1}^{n}\left\{2\widehat{\boldsymbol{\mathfrak{T}}}_i\boldsymbol{G}_i(\widehat{q},\widehat{\Lambda}_0)^T+2\boldsymbol{G}_i(\widehat{q},\widehat{\Lambda}_0)\widehat{\boldsymbol{\mathfrak{T}}}_i^T\right\}\right], \tag{E.12}$$

where $\widehat{\boldsymbol{\Sigma}}$ is the estimator proposed in the main paper and $\widehat{\boldsymbol{\mathfrak{T}}}_i=\boldsymbol{\Psi}_i(\widehat{q},\widehat{\Lambda}_0)$. The second term

in (E.12) effects a bias correction that will be nonnegligible when n is not large, so that (E.12) provides a more faithful representation of the uncertainty in estimation of $\boldsymbol{\Sigma}$.

If in a SMART the randomization probabilities are estimated or modeled as discussed in Web Appendix D, with the possible addition of basis functions of the history to enhance efficiency/power, to obtain a bias-corrected covariance matrix estimator, form all of the quantities above involving $\Omega(u, d^j)$, $j = 1, \ldots, D$, by substituting the fitted models $\omega_k(\boldsymbol{h}_k, a_k; \widehat{\boldsymbol{\gamma}})$, $k = 1, \ldots, K$, and replace $\widehat{\boldsymbol{\Sigma}}$ and $\widehat{\boldsymbol{\mathfrak{T}}}_i$ in (E.12) by $\widehat{\boldsymbol{\Sigma}}_\gamma$ and $\widehat{\boldsymbol{\mathfrak{T}}}_i^R(\widehat{\boldsymbol{\gamma}})$.

Web Appendix F: Simulation Details

F.1. Details for Scenarios 1-3

Scenarios 1 and 2 involve a SMART as in Figure **??** of the main paper without the control regime, so with four embedded regimes, and use the generative process of Kidwell and Wahed (2013), modified to possibly involve covariates. We first generated baseline covariate $X_1 \sim \mathcal{N}(0, 1)$ and stage 1 treatment A_1 as Bernoulli(0.5). We then generated response status R as Bernoulli(0.4); and, if $R = 1$, intermediate covariate X_2 as Bernoulli(p_{X_2}), where, for $\boldsymbol{\theta}_{X_2} = (\theta_{X_2,1}, \theta_{X_2,2}, \theta_{X_2,3})^T$, $p_{X_2} = \text{expit}(\theta_{X_2,1} + \theta_{X_2,2}X_1 + \theta_{X_2,3}A_1)$ and $\text{expit}(u) = e^u/(1 + e^u)$, and A_2 as Bernoulli(0.5), so that $\boldsymbol{X}_2 = (R, X_2)^T$. Then, given $\boldsymbol{\theta} = (\theta_1^{NR}, \theta_0^{NR}, \theta_1^R, \theta_0^R, \theta_{11}^{RE}, \theta_{10}^{RE}, \theta_{01}^{RE}, \theta_{00}^{RE})^T$, $\boldsymbol{\delta}^{NR} = (\delta_1^{NR}, \delta_0^{NR})^T$, $\boldsymbol{\delta}^R = (\delta_1^R, \delta_0^R)^T$, $\boldsymbol{\alpha}_\ell = (\alpha_{\ell,11}, \alpha_{\ell,10}\alpha_{\ell,01}\alpha_{\ell,00})^T$, $\ell = 1, 2$, with $\lambda_r^{NR} = \theta_r^{NR}\exp(\delta_r^{NR}X_1)$, $\lambda_r^R = \theta_r^R\exp(\delta_r^R X_1)$, $r = 0, 1$, and $\lambda_{rs}^{RE} = \theta_{rs}^{RE}\exp\{\alpha_{1,rs}X_1 + \alpha_{2,rs}(X_2 - p_{X_2})\}$, $r, s = 0, 1$, for $R = 0$, we generated potential event times T_r^{NR}, $r = 0, 1$, as exponential(λ_r^{NR}); and, for $R = 1$, potential times to response T_r^R, $r = 0, 1$, as exponential(λ_r^R) and times from response to event T_{rs}^{RE} as exponential(λ_{rs}^{RE}), $r, s = 0, 1$. With $T^{NR} = A_1T_1^{NR} + (1 - A_1)T_0^{NR}$ for $R = 0$ and $T^R = A_1\{A_2(T_1^R + T_{11}^{RE}) + (1 - A_2)(T_1^R + T_{10}^{RE})\} + (1 - A_1)\{A_2(T_0^R + T_{01}^{RE}) + (1 - A_2)(T_0^R + T_{00}^{RE})\}$ for $R = 1$, the event time was $T = RT^R + (1 - R)T^{NR}$. Censoring time C was generated as

uniform$(0, c_{\max})$, and $U = \min(T, C)$, $\Delta = I(T \leqslant C)$. If the event time was censored prior to response, we redefined $R = 0$. Thus, for $R = 1$, the time to Decision 2 $\mathcal{T}_2 = A_1 T_1^R + (1 - A_1) T_0^R$ and $\kappa = 2$, and, as nonresponders never reach Decision 2, $\kappa = 1$ for $R = 0$.

In Scenario 1, we took $c_{\max} = 3.80$, resulting in 30% to 40% censoring. Under H_0, Scenario 1(a) takes $\boldsymbol{\theta} = (1/0.91, 1/0.91, 1/0.5, 1/0.5, 1, 1, 1, 1)^T$, with $\boldsymbol{\theta}_{X_2}$, $\boldsymbol{\delta}^R$, $\boldsymbol{\delta}^{NR}$, $\boldsymbol{\alpha}_\ell$, $\ell = 1, 2$, vectors of zeroes, so that time to event is not associated with covariates, and thus duplicates the first null hypothesis scenario of Kidwell and Wahed (2013). Scenario 1(b) is the same but with covariate associations induced by taking $\boldsymbol{\theta}_{X_2} = (0, 0.15, 0)^T$, $\boldsymbol{\delta}^R = (0.7, 0.7)^T$, $\boldsymbol{\delta}^{NR} = (0.3, 0.3)^T$, $\boldsymbol{\alpha}_\ell = (0.7, 0.7, 0.7, 0.7)^T$, $\ell = 1, 2$. In Table **??** of the main paper, under both 1(a) and 1(b), all tests are anti-conservative for $n = 250$. Those based on $\mathbb{Z}_{\mathrm{C,nocov}}$, $\mathbb{Z}_{\mathrm{C,cov}}$, and $\mathbb{Z}_{\mathrm{KW}}$ achieve the nominal level for $n = 500, 1000$; including unimportant covariates as in 1(a) does not degrade performance. The tests based on $\mathbb{Z}_{\mathrm{U,nocov}}$ and $\mathbb{Z}_{\mathrm{U,cov}}$ are anti-conservative, supporting use of the bias correction. As an alternative to H_0 under 1(b), we took $\boldsymbol{\theta} = (1/0.91, 1/1.15, 1/0.9, 1/0.5, 1/2, 1/2.33, 1/1.11, 1/0.67)^T$, with all other quantities the same. From Table **??** of the main paper, for tests that achieve the nominal level under H_0, incorporation of covariates associated with the event time using the proposed methods yields increases in power.

As discussed in Section F.2 next, the test of Kidwell and Wahed (2013) may be expected to be robust to departure from the martingale property under Scenario 1. Under H_0, Scenarios 2(a) and 2(b) are the same as 1(a) and 1(b) except with $c_{\max} = 8$, resulting in 25% to 30% censoring, and $\boldsymbol{\theta} = (1/0.91, 1/0.91, 1/0.5, 1/0.5, 1/3, 1/3, 1/3, 1/3)^T$. As Table **??** shows, all tests are anti-conservative with $n = 250$; however, for $n = 500, 1000$, the tests based on $\mathbb{Z}_{\mathrm{C,nocov}}$ and $\mathbb{Z}_{\mathrm{C,cov}}$ achieve the nominal level, while those based on $\mathbb{Z}_{\mathrm{KW}}$ continue to be anti-conservative. In Section F.2, we speculate that this behavior may reflect a strong departure from the martingale property for these Scenarios. For an alternative to Scenario 2(b), we

took instead $\boldsymbol{\theta} = (1/0.35, 1/0.9, 1/0.5, 1/0.5, 1/3.3, 1/3.3, 1/3, 1/3)^T$; again, Table **??** of the main paper shows gains in power when associated covariates are incorporated in the proposed methods.

Scenario 3 uses a different generative strategy and mimics the design of Study C9710. We first generated $\boldsymbol{X}_1 = (X_{11}, X_{12})^T$, where $X_{11} \sim \mathcal{N}(0,1)$ and $X_{12} \sim$ uniform$(0,1)$, and A_1 as Bernoulli(0.5). For given α_{1D}, α_{1SS}, α_{2AL}, ψ, and ζ, we took $\boldsymbol{\theta}_{1D} = (\alpha_{1D}, 0.5\psi, 0.5\psi, -0.26\zeta)^T$ and $\boldsymbol{\theta}_{1SS} = (\alpha_{1SS}, 0.5\psi, 0.5\psi, 0.24\zeta)^T$, generated potential event time T_D as exponential(λ_{1D}), $\lambda_{1D} = \exp\{\theta_{1D,1} + \theta_{1D,2}X_{11} + \theta_{1D,3}(X_{12} - 0.5) + \theta_{1D,4}(A_1 - 0.5)\}$ and potential time to Decision 2 T_{SS} as exponential(λ_{1SS}), $\lambda_{1SS} = \exp\{\theta_{1SS,1} + \theta_{1SS,2}X_{11} + \theta_{1SS,3}(X_{12} - 0.5) + \theta_{1SS,4}(A_1 - 0.5)\}$, and took $S = \min(T_D, T_{SS})$, $R = I(T_{SS} < T_D)$. If $R = 1$, with $\boldsymbol{\theta}_{X_2} = (0.2, 0.5\psi, 0.4\psi, 0.12\zeta)^T$ and $\boldsymbol{\theta}_{2AL} = (\alpha_{2AL}, 0.5\psi, -0.52\psi, 0.6\psi, -0.1\zeta, -0.11\zeta)^T$, we generated X_2 as Bernoulli(p_{X_2}), $p_{X_2} = \text{expit}(\theta_{X_2,1} + \theta_{X_2,2}X_{11} + \theta_{X_2,3}X_{12} + \theta_{X_2,4}A_1)$, A_2 as Bernoulli(0.5), and "added life" post-response T_{AL} as exponential$(\lambda_{2,AL})$, $\lambda_{2AL} = \exp\{\theta_{2AL,1} + \theta_{2AL,2}X_{11} + \theta_{2AL,3}(X_{12} - 0.5) + \theta_{2AL,4}(X_2 - p_{X2}) + \theta_{2AL,5}(A_1 - 0.5) + \theta_{2AL,6}(A_2 - 0.5)\}$. We then took $T = (1-R)T_D + R(T_{SS} + T_{AL})$ and, with C uniform$(0, c_{\max})$, $U = \min(T, C)$, $\Delta = I(T \leqslant C)$; if $R = 1$ and $C < S$, redefine $R = 0$. For $R = 1$, $\mathcal{T}_2 = T_{SS}$ and $\kappa = 2$; else, $\kappa = 1$.

Scenarios 3(a) - 3(c) reflect different possible departures from the martingale property through specification of α_{1D} and α_{2AL}; the property roughly holds with $\alpha_{1D} = \alpha_{2AL}$. In all cases, $\alpha_{1SS} = -4.2$, $\psi = 1.5$, and $\zeta = 0$ corresponds to H_0 while $\zeta > 0$ produces alternatives to H_0. For Scenarios 3(a) and 3(b), $c_{\max} = 500$, resulting in about 40% to 45% censoring; for Scenario 3(c), $c_{\max} = 300$ for about 30% censoring. For Scenario 3(a), $\alpha_{1D} = \alpha_{2AL} = -5.5$; from Table **??** of the main paper, under H_0, the tests based on $\mathbb{Z}_{\text{C,nocov}}$, $\mathbb{Z}_{\text{C,cov}}$, and $\mathbb{Z}_{\text{KW}}$ all achieve the nominal level, and incorporation of covariates yields increased power under alternatives. Under Scenarios 3(b) and 3(c), $\alpha_{1D} = -4.5$, $\alpha_{1AL} = -5.5$ and $\alpha_{1D} = -5.5$, $\alpha_{1AL} = -3.5$, respectively. While the tests based on $\mathbb{Z}_{\text{C,nocov}}$ and $\mathbb{Z}_{\text{C,cov}}$ achieve the nominal

level under H_0 in both cases for all n, that based on $\mathbb{Z}_{\mathrm{KW}}$ is conservative under 3(b) and anti-conservative under 3(c), which persists across all n, possibly reflecting departure from the martingale property; see Web Appendix F.2.

F.2. Implications of martingale property

We examine the implications of Section D.4 of Web Appendix D, namely, that the martingale property need not hold, for the data generative Scenarios 1-3, which may offer insight into the nature of the simulation results.

First consider Scenarios 1(a) and 2(a) under the null hypothesis; as noted in Section **??** of the main paper, Scenario 1(a) is the first null scenario of Kidwell and Wahed (2013). Let $\pi_R = P(R = 1)$; $\pi_R = 0.4$ in Scenarios 1(a) and 2(a). Because H_0 holds, we suppress the subscripts indicating treatment; thus, for $R = 0$, let T^{NR}, the potential event time, be exponential(λ_1). If $R = 1$, let the potential time to response, T^R, be exponential(λ_2) and the potential time to event, T^{RE}, be exponential(λ_3). Then it is straightforward to show that the hazard of experiencing the event prior to response at time u is given by

$$\frac{(1-\pi_R)\lambda_1 \exp(-\lambda_1 u)}{(1-\pi_R)\exp(-\lambda_1 u) + \pi_R \exp(-\lambda_2 u)}. \tag{F.1}$$

The hazard of experiencing the event after response ($R = 1$) is equal to λ_3 from above. According to Web Appendix D.4, the martingale property will hold if (F.1) is equal to λ_3. Moreover, it can be shown that

$$\lambda_0(u) = \frac{(1-\pi_R)\lambda_1 \exp(-\lambda_1 u) + \pi_R \lambda_2 \lambda_3 \{\exp(-\lambda_3 u) - \exp(-\lambda_2 u)\}/(\lambda_2 - \lambda_3)}{(1-\pi_R)\exp(-\lambda_1 u) + \pi_R\{\lambda_2 \exp(-\lambda_3 u) - \lambda_3 \exp(-\lambda_2 u)\}/(\lambda_2 - \lambda_3)}. \tag{F.2}$$

In Scenario 1(a), $\lambda_1 = 1/0.91 = 1.099$, $\lambda_2 = 1/0.5 = 2$, and $\lambda_3 = 1$. In Figure 1, for these values and $\pi_R = 0.4$ we plot (F.1) (dashed line), λ_3 (solid line), and (F.2) (dotted line). The hazard in (F.1) differs from λ_3 over most of the time range, but the greatest disparity, for small u, is not substantial. We conjecture that this relatively mild departure from the martingale property is reflected in the fact that the test of Kidwell and Wahed (2013) achieves the nominal level. In contrast, Figure 2 shows the same plot for Scenario

2(a), for which $\lambda_1 = 1/0.91 = 1.099$, $\lambda_2 = 1/0.5 = 2$, and $\lambda_3 = 1/3$. Here, the disparity between λ_3 (solid) and (F.1) (dashed) is substantial, and we we conjecture that the anti-conservatism of the test of Kidwell and Wahed (2013) in this case, which persists as the sample size increases, is a consequence of this feature.

[Figure 1 about here.]

[Figure 2 about here.]

Consider now Scenario 3 under the null hypothesis ($\zeta = 0$) in the simplified case in which there are no covariate effects, so that $\psi = 0$. Under this generative scenario, the hazard of experiencing the event prior to response at time u is given by $\lambda_1 = \exp(\alpha_{1D})$ and the hazard of experiencing the event after response ($R = 1$) is given by $\lambda_3 = \exp(\alpha_{2AL})$. Moreover, the hazard for the potential time to Decision 2 $\lambda_2 = \exp(\alpha_{1SS})$, and

$$\lambda_0(u) = \frac{(\lambda_1 + \lambda_2)(\lambda_1 - \lambda_3)\exp\{-(\lambda_1 + \lambda_2)u\} + \lambda_2\lambda_3\exp(-\lambda_3 u)}{(\lambda_1 - \lambda_3)\exp\{-(\lambda_1 + \lambda_2)u\} + \lambda_2\exp(-\lambda_3 u)}. \tag{F.3}$$

According to Web Appendix D.4, the martingale property holds in this setting if $\lambda_1 = \lambda_3$. In Scenario 3(a), $\alpha_{1D} = \alpha_{2AL} = -5.5$; thus, without covariate effects ($\psi = 0$), the martingale property holds exactly, and likely holds approximately when covariate effects are included as in Section **??** ($\psi = 1.5$). As seen in Table **??** of the main paper, the test of Kidwell and Wahed (2013) achieves the nominal level, as expected under these conditions. In Scenario 3(b), $\alpha_{1D} = -4.5$ and $\alpha_{2AL} = -5.5$, so that the martingale property does not hold exactly or approximately; we conjecture that the conservatism of the test of Kidwell and Wahed (2013) in this case, which persists as the sample size increases, is a consequence of this feature. In Scenario 3(c), $\alpha_{1D} = -5.5$ and $\alpha_{2AL} = -3.5$, so that the martingale property does not hold exactly or approximately. Again, we conjecture that the anti-conservatism of the test of Kidwell and Wahed (2013) reflects this feature.

F.3. Details for Scenarios 4 and 5

Scenario 4, which is based on the SMART design in Figure **??** of the main paper, with an additional control regime, is similar to Scenario 3, with the following modifications to incorporate the control regime. Code the control as $a_1 = 2$, so that $\mathcal{A}_1 = \{0, 1, 2\}$. We generated A_1 as multinomial with probabilities 1/3, 1/3, 1/3 for $a_1 = 0, 1, 2$, respectively. For given $\alpha_{1D} = -5.5$, $\alpha_{1SS} = -4.2$, $\alpha_{2AL} = -5.5$, $\psi = 1.5$, and ζ, we took $\boldsymbol{\theta}_{1D} = (\alpha_{1D}, 0.5\psi, 0.5\psi, -0.26\zeta, 0.15\zeta)^T$ and $\boldsymbol{\theta}_{1SS} = (\alpha_{1SS}, 0.5\psi, 0.5\psi, 0.24\zeta - 0.13\zeta)^T$, generated potential event time T_D as exponential(λ_{1D}), $\lambda_{1D} = \exp\{\theta_{1D,1} + \theta_{1D,2}X_{11} + \theta_{1D,3}X_{12} + \theta_{1D,4}I(A_1 = 1) + \theta_{1D,5}I(A_1 = 2)\}$ and potential time to Decision 2 T_{SS} as exponential(λ_{1SS}), $\lambda_{1SS} = \exp\{\theta_{1SS,1} + \theta_{1SS,2}X_{11} + \theta_{1SS,3}X_{12} + \theta_{1SS,4}A_1\}$, and took $S = \min(T_D, T_{SS})$ and $R = I(T_{SS} < T_D)$ as in the main paper. If $R = 1$, with $\boldsymbol{\theta}_{X_2} = (0.2, 0.5\psi, 0.4\psi, 0.12\zeta, 0.1\zeta)^T$ and $\boldsymbol{\theta}_{2AL} = (\alpha_{2AL}, 0.5\psi, -0.52\psi, 0.6\psi, -0.1\zeta, 0.15\zeta, -0.11\zeta)^T$, we generated X_2 as Bernoulli(p_{X_2}), $p_{X_2} = \text{expit}\{\theta_{X_2,1}+\theta_{X_2,2}X_{11}+\theta_{X_2,3}X_{12}+\theta_{X_2,4}I(A_1 = 1)+\theta_{X_2,5}I(A_1 = 2)\}$, A_2 as Bernoulli(0.5), and "added life" post-response T_{AL} as exponential($\lambda_{2,AL}$), $\lambda_{2AL} = \exp\{\theta_{2AL,1} + \theta_{2AL,2}X_{11} + \theta_{2AL,3}X_{12} + \theta_{2AL,4}X_2 + \theta_{2AL,5}I(A_1 = 1) + \theta_{2AL,6}I(A_1 = 2) + \theta_{2AL,7}A_2I(A_1 < 2)\}$. All other features of the scenario are as in Scenario 3.

For Scenario 5, data generation is as in Scenario 3 at the first stage of the design, with $\alpha_{1D} = -5.5$ and $\alpha_{1SS} = -3.5$, $\psi = 1.5$, and $\boldsymbol{\theta}_{1D}$ and $\boldsymbol{\theta}_{1SS}$ as in Scenario 3 but with potential event time T_D as exponential(λ_{1D}), $\lambda_{1D} = \exp(\theta_{1D,1} + \theta_{1D,2}X_{11} + \theta_{1D,3}X_{12} + \theta_{1D,4}A_1)$ and potential time to Decision 2 T_{SS} as exponential(λ_{1SS}), $\lambda_{1SS} = \exp(\theta_{1SS,1} + \theta_{1SS,2}X_{11} + \theta_{1SS,3}X_{12} + \theta_{1SS,4}A_1)$. We then took $S = \min(T_D, T_{SS})$ as before and additionally $\Gamma_{SS} = I(T_{SS} < T_D)$. For subjects who would reach Decision 2, with $\Gamma_{SS} = 1$, we generated response status R as Bernoulli(p_R), where $p_R = \text{expit}(0.3 + 0.15X_{11} + 0.15X_{12} + 0.2\zeta A_1)$, and X_2 as in Scenario 3. For these subjects with $\Gamma_{SS} = 1$, we generated A_2 as Bernoulli(0.5) within their observed value of (A_1, R). Then with $\boldsymbol{\theta}_{2AL} =$

$(\alpha_{2AL}, 0.5\psi, -0.52\psi, 0.6\psi, -0.1\zeta, -0.11\zeta, -0.3\zeta)^T$, we generated "added life" post-response T_{AL} as exponential$(\lambda_{2,AL})$, $\lambda_{2AL} = \exp\{\theta_{2AL,1} + \theta_{2AL,2}X_{11} + \theta_{2AL,3}X_{12} + \theta_{2AL,4}X_2 + \theta_{2AL,5}A_1 + \theta_{1AL,6}A_2 + \theta_{1AL,7}R\}$. We then took $T = (1 - \Gamma_{SS})T_D + \Gamma_{SS}(T_{SS} + T_{AL})$ and, with C uniform$(0, c_{\max})$, $U = \min(T, C)$, $\Delta = I(T \leqslant C)$. If $\Gamma_{SS} = 1$ but $C < T_{SS}$ or $\Gamma_{SS} = 0$, take R to be undefined. For subjects for whom $\Gamma_{SS} = 1$ and $C \geqslant T_{SS}$, $\mathcal{T}_2 = T_{SS}$ and $\kappa = 2$; if $\Gamma_{SS} = 1$ and $C < T_{SS}$ or $\Gamma_{SS} = 0$, $\kappa = 1$.

F.4. Simulation studies of tests of pairwise comparisons of regimes

The test statistic of Kidwell and Wahed (2013) involves a (3×1) vector, where each component addresses a pairwise comparison of the hazards for two of the four regimes in a SMART analogous to that in Figure **??** of the main paper without the additional control regime. Specifically, as in the figure, represent these regimes as being of the form "Give treatment a initially; if the event does not occur before response status is ascertained and nonresponse, continue, otherwise, if response, give treatment b," the four regimes correspond to $(a, b) = (0, 0), (0, 1), (1, 0), (1, 1)$. Denoting these as Regimes 1, 2, 3, and 4 in this order, the three components involve the comparisons of 3 versus 4, 2 versus 4, and 1 versus 4; that is, Regime 4 is the reference regime against which the other three regimes are compared. The comparison of Regimes 3 and 4 involves "shared path" regimes starting with the same stage 1 treatment, while the other two comparisons start with different stage 1 treatments. The test statistic of Kidwell and Wahed (2013) is formed as a quadratic form involving this (3×1) vector and an approximation to its sampling covariance matrix using martingale theory, which, as discussed in Section **??**, may be violated. It is thus possible to construct from these elements three test statistics as a component of the vector divided by its standard error obtained from this sampling covariance matrix, where these test statistics address the comparisons of Regimes 3 versus 4, 2 versus 4, and 1 versus 4, respectively.

The above configuration is the situation in simulation Scenarios 1 - 3 in Section **??** of the main paper. Study C9710,, depicted in Figure **??** of the main paper, is analogous, involving four embedded regimes of the form "Give consolidation therapy a; if subject completes consolidation (responder) before the event occurs, give maintenance therapy b," which we denote as Regimes 1, 2, 3, and 4 as $(a, b) = (0, 0), (0, 1), (1, 0), (1, 1)$.

To evaluate the performance of the proposed methods and the test of Kidwell and Wahed (2013) for making pairwise comparisons, we considered Scenario 1(a), which as in the main paper and Web Appendix F is expected to be robust to departure from the martingale property; Scenario 2(a), which involves a strong departure from the martingale property; Scenario 3(a), for which the martingale property holds roughly except for covariate associations; and Scenario 3(c), which reflects a departure from the martingale property and includes covariate associations. For each scenario, we used the test statistics obtained from the overall test statistic of Kidwell and Wahed (2013) as described above and the various versions of the proposed test statistic denoted as $\mathbb{Z}_{\text{U,nocov}}$, $\mathbb{Z}_{\text{U,nocov}}$, $\mathbb{Z}_{\text{U,cov}}$, and $\mathbb{Z}_{\text{C,cov}}$ in the main paper, where $\mathcal{D}$ was taken to be the subset of embedded regimes involved in the indicated pairwise comparison (so $D = 2$).

Table 1 shows the results. Under the null hypothesis, under Scenarios 1(a) and 3(a), the proposed methods with using the bias-corrected covariance matrix and the test statistics of Kidwell and Wahed (2013) mostly achieve the nominal level, reflecting for the latter that the martingale property is not significantly violated. However, under Scenarios 2(a) and 3(c), although the proposed methods continue to achieve the nominal level, that of Kidwell and Wahed (2013) for testing the "shared path" comparison (3 versus 4) in Scenario 2(a) is anti-conservative, suggesting that the anti-conservative performance of the overall test shown in Table **??** of the main paper stems from this comparison. Likewise, under Scenario 3(c), although the test of Kidwell and Wahed (2013) achieves the nominal level

for the "shared path" comparison (3 versus 4), the non-shared path comparisons (2 versus 4, 1 versus 4) exhibit anti-conservatism, suggesting that the performance of the overall test shown in Table **??** of the main paper reflects this behavior. We also show power under the same alternatives in Section **??** of the main paper for Scenarios 3(a) and 3(c), which involve covariates associated with the event time; the comparison of Regimes 2 versus 4 is omitted under Scenario 3(c) because the hazards for these regimes are very similar, so that power is extremely low using all test statistics. As for the proposed tests of the overall null hypothesis in (**??**) of the main paper, the proposed tests of pairwise comparisons show enhanced power when covariates are incorporated.

[Table 1 about here.]

F.5. Simulation study of comparison of more complex regimes

To demonstrate that the proposed methods can be used to compare the regimes in an arbitrary set of regimes that are "feasible" given the available data in the sense discussed in Tsiatis et al. (2020, Section 6.2.3), consider Scenario 3(a) of Section **??** of the main paper, where $\mathcal{A}_1 = \{0, 1\}$ and $\mathcal{A}_2 = \{0, 1\}$. Recall that by construction the covariates are associated with the event time outcome. We take the set of regimes of interest to be $\mathcal{D} = \{d^1, d^2, d^3\}$ ($D = 3$), where the regimes are defined as follows:

- Regime 1, d^1, with rules $d_1^1(\boldsymbol{h}_1) = I(X_{12} \geqslant 0.3)$, $d_2^1(\boldsymbol{h}_2) = I(X_{12} \geqslant 0.4, X_2 = 1, R = 1)$
- Regime 2, d^2, with rules $d_1^2(\boldsymbol{h}_1) = I(X_{12} \leqslant 0.5)$, $d_2^2(\boldsymbol{h}_2) = I(X_{12} \geqslant 0.6, X_2 = 1, R = 1)$
- Regime 3, d^3, with rules $d_1^3(\boldsymbol{h}_1) = I(X_{12} \geqslant 0.7)$, $d_2^3(\boldsymbol{h}_2) = I(X_{12} \geqslant 0.8, X_2 = 0, R = 1)$.

The choice of these regimes is completely arbitrary and for demonstration only.

Table 2 shows the results under the null hypothesis of no difference in hazard rate among these three regimes and demonstrates that the proposed methods yield a test achieving the nominal level of significance.

[Table 2 about here.]

F.6. Simulation study of three decision SMART

All of the previous simulations involve SMARTs with two decision points, which is arguably by far the most common situation in practice. To demonstrate that the proposed methods can be used in the more complex setting of a SMART with three decision points, we consider an extension of Scenario 3 to involve an additional decision point. The generative scenario is as follows and is such that the SMART embeds eight regimes in which subjects who reach the second and third decision points are are re-randomized.

We first generated $\boldsymbol{X}_1 = (X_{11}, X_{12})^T$, where $X_{11} \sim \mathcal{N}(0,1)$ and $X_{12} \sim \text{uniform}(0,1)$, and A_1 as Bernoulli(0.5). For given α_{1D}, α_{1SS}, ψ, and ζ, we took $\boldsymbol{\theta}_{1D} = (\alpha_{1D}, 0.5\psi, 0.5\psi, -0.26\zeta)^T$ and $\boldsymbol{\theta}_{1SS} = (\alpha_{1SS}, 0.5\psi, 0.5\psi, 0.24\zeta)^T$, generated potential event time T_{D1} as exponential(λ_{1D}), $\lambda_{1D} = \exp\{\theta_{1D,1} + \theta_{1D,2}X_{11} + \theta_{1D,3}(X_{12} - 0.5) + \theta_{1D,4}(A_1 - 0.5)\}$ and potential time to Decision 2 T_{SS} as exponential(λ_{1SS}), $\lambda_{1SS} = \exp\{\theta_{1SS,1} + \theta_{1SS,2}X_{11} + \theta_{1SS,3}(X_{12} - 0.5) + \theta_{1SS,4}(A_1 - 0.5)\}$, and took $S_2 = \min(T_{D1}, T_{SS})$, $R_2 = I(T_{SS} < T_{D1})$. If $R_2 = 1$, with $\boldsymbol{\theta}_{X_2} = (0.2, 0.5\psi, 0.4\psi, 0.12\zeta)^T$, we generated X_2 as Bernoulli(p_{X_2}), $p_{X_2} = \text{expit}(\theta_{X_2,1} + \theta_{X_2,2}X_{11} + \theta_{X_2,3}X_{12} + \theta_{X_2,4}A_1)$, and A_2 as Bernoulli(0.5). For given α_{2D} and α_{2TS}, we took $\boldsymbol{\theta}_{2D} = (\alpha_{2D}, 0.5\psi, -0.52\psi, 0.6\psi, -0.1\zeta, -0.11\zeta)^T$ and $\boldsymbol{\theta}_{2TS} = (\alpha_{2TS}, 0.5\psi, -0.52\psi, 0.6\psi, -0.1\zeta, -0.11\zeta)^T$, and generated potential event time T_{D2} as exponential(λ_{2D}), $\lambda_{2D} = \exp\{\theta_{2D,1} + \theta_{2D,2}X_{11} + \theta_{2D,3}(X_{12} - 0.5) + \theta_{2D,4}(X_2 - p_{X_2}) + \theta_{2D,5}(A_1 - 0.5) + \theta_{2D,6}(A_2 - 0.5)\}$ and potential time to Decision 3 T_{TS} as exponential(λ_{2TS}), $\lambda_{2TS} = \exp\{\theta_{2TS,1} + \theta_{2TS,2}X_{11} + \theta_{2TS,3}(X_{12} - 0.5) + \theta_{2TS,4}(X_2 - p_{X_2}) + \theta_{2TS,5}(A_1 - 0.5) + \theta_{2TS,6}(A_2 - 0.5)\}$, and took $S_3 = \min(T_{SS} + T_{D2}, T_{SS} + T_{TS})$, $R_3 = I(T_{TS} < T_{D2})$. With $\boldsymbol{\theta}_{X_3} = (0.2, 0.5\psi, 0.4\psi, 0.12\zeta, -0.15\zeta)^T$, if $R_3 = 1$, we generated X_3 as Bernoulli(p_{X_3}), $p_{X_3} = \text{expit}(\theta_{X_3,1} + \theta_{X_3,2}X_{11} + \theta_{X_3,3}X_{12} + \theta_{X_3,4}X_2 + \theta_{X_3,5}A_1 + \theta_{X_3,6}A_2)$, and A_3 as Bernoulli(0.5). Finally, for given α_{3AL}, with $\boldsymbol{\theta}_{3AL} = (\alpha_{3AL}, 0.5\psi, -0.52\psi, 0.6\psi, 0.1\psi, -0.1\zeta, -0.11\zeta, 0.2\zeta)^T$, we generated "added life" T_{AL} as exponential(λ_{3AL}), $\lambda_{3AL} = \exp\{\theta_{3AL,1} + \theta_{3AL,2}X_{11} +$

$\theta_{3AL,3}(X_{12}-0.5)+\theta_{3AL,4}(X_2-p_{X2})+\theta_{3AL,5}(X_3-p_{X3})+\theta_{1AL,6}(A_1-0.5)+\theta_{1AL,7}(A_2-0.5)+\theta_{1AL,8}(A_3-0.5)\}$. We then took $T=(1-R_2)T_{D1}+R_2(1-R_3)(T_{SS}+T_{D2})+R_2R_3(T_{SS}+T_{TS}+T_{AL})$, and, with C uniform$(0,c_{\max})$, $U=\min(T,C)$, $\Delta=I(T\leqslant C)$; if $R_2=1$ and $C<S_2$, redefine $R_2=0$, and if $R_3=1$ and $C<S_3$, redefine $R_3=0$. For $R_2=1$, $\mathcal{T}_2=T_{SS}$ and, if $R_3=0$, $\kappa=2$. If $R_2=1$ and $R_3=1$, $\mathcal{T}_3=T_{SS}+T_{TS}$, $\kappa=3$; else, $\kappa=1$.

Under this scenario, the eight regimes are of the form "Give treatment a initially; if the event does not occur before treatment with a concludes, continue to Decision 2 and give treatment b; if the event does not occur before treatment with b concludes, continue to Decision 3 and give treatment c," The eight regimes, which we denote in order as regimes 1, 2, …, 8, correspond to $(a,b,c)=(0,0,0),(0,1,0),(1,0,0),(1,1,0),(0,0,1),(0,1,1),(1,0,1),(1,1,1)$.

For the simulations reported in Table 3, we took $\zeta=0.0$, so focusing on performance under the null hypothesis; $\alpha_{1D}=-4.5$, $\alpha_{1SS}=-3.2$, $\alpha_{2D}=-4.0$, $\alpha_{2TS}=-2.7$, and $\alpha_{3AL}=-3.0$; $\psi=1.5$; and $c_{\max}=350$, corresponding to about 15% censoring. The comparison of regimes 1 and 8 corresponds to comparison of "non-shared path" regimes; that of regimes 1 and 2 corresponds to comparison of "shared path" regimes that share the same first stage treatment; and that of regimes 1 and 5 corresponds to comparison of "shared path" regimes that share the same first and second stage treatments. The results show that, for comparison of all eight embedded regimes, all tests are generally anti-conservative until $n=4000$, where $\mathbb{Z}_{\mathrm{C,nocov}}$ and $\mathbb{Z}_{\mathrm{C,cov}}$ achieve the nominal level (within Monte Carlo error), suggesting that large sample sizes may be needed to achieve the nominal level for an overall test. This result may not be too surprising, as with three decision points the numbers of subjects with experience consistent with any regime may be small when n is not large. Comparison of pairs of "non-shared path" and "shared path" regimes can be carried out reliably with a much smaller overall sample size, as the nominal level is achieved for the tests based on $\mathbb{Z}_{\mathrm{C,nocov}}$ and $\mathbb{Z}_{\mathrm{C,cov}}$. Not surprisingly, comparison of four "shared path" regimes

requires a larger sample size to achieve the nominal level, but considerably smaller than that required for the overall comparison of all eight regimes.

[Table 3 about here.]

Web Appendix G: Data Analysis Details

G.1. Data definitions and data cleaning

The data from North American Leukemia Intergroup Study C9710 provided to us by the Alliance for Clinical Trials in Oncology comprise information on 538 subjects; this data set is the same as that considered by Hager et al. (2018). Ten (10) baseline covariates (components of $\boldsymbol{X}_1$) were ideally collected on each subject at the time of registration into the trial: age, gender, race, ethnicity, Eastern Cooperative Oncology Group (ECOG) performance status, risk group, white blood cell count, platelet count, serum creatinine, and hemoglobin. Table 4 shows these covariates. For responders, $\boldsymbol{X}_2$ includes variables summarizing adverse events and toxicities occurring during induction or consolidation therapy. We consider only adverse events experienced by at least 5% of patients. Table 5 shows the adverse events, which are coded with an eight-digit Medical Dictionary for Regulatory Activities (medDRA) code. In the data, the adverse events were recorded as occurring during induction therapy (coded by "I"), during consolidation therapy (coded by "C"), and during either induction or consolidation therapy (coded by "IC"). Thus, the binary variable "IC_90004060" represents whether or not hemorrhage or bleeding with grade 3 or 4 thrombocytopenia occurred during either induction or consolidation therapy. There are 46 such variables in $\boldsymbol{X}_2$; three of the variables are trinary; all others are binary.

[Table 4 about here.]

[Table 5 about here.]

For the data analysis reported in Section **??** of the main paper, we considered a subset of these data, similar to Hager et al. (2018). First, the following subjects were removed from the data set: 57 subjects deemed ineligible according to the age criterion in Powell et al. (2010), 7 subjects with more than two covariates missing, and 6 subjects for whom the date of initiation of induction therapy is missing. Fifteen (15) subjects are missing the start date of maintenance therapy; the actual event or censoring time for these subjects was artificially censored at the start date of consolidation therapy. One (1) such subject is also missing the start date of consolidation therapy; this subject was also removed from the data set (Hager et al. (2018) did not remove this subject). After these deletions, $n = 467$ subjects were available for analysis.

Of these subjects, the first 50 to enroll in the study had different maintenance treatment options; of the 47 of these subjects not already removed, all reached stage 2, and the actual event or censoring time for these subjects was artificially censored at the start date of maintenance therapy. For four (4) other subjects, the start date for maintenance therapy was recorded as being before or equal to the date of registration in the study; the actual event/censoring time for these subjects was artificially censored at the date of start of consolidation therapy.

Taking into account all of these conventions, among the $n = 467$ subjects, 310 reached stage 2 and were randomized to maintenance therapy options.

As in Hager et al. (2018), several of the baseline covariates were redefined as follows:

- The number of race groups is reduced to only include “White,” “Hispanic American,” “Black/African American,” and “Other.” Patients previously classified as “Asian,” “Native Hawaiian or Pacific Islander,” “American Indian or Alaska Native,” “Indian Subcontinent,” or “Multiple Races Reported” are classified as “Other.”
- 1 subject has a platelet count of 5300, which is much higher than those for all other

subjects, who had values in the range 1–232. This observation was divided by 100 for consistency with the range given in Powell et al. (2010).

- 4 subjects have hemoglobin levels of 87, 92, 81, and 80, which are much higher than those for the rest of the subjects, who had values in the range of 4.3–14.6. It is believed that these values were entered in units of g/L instead of g/dL, so we divided these hemoglobin observations by 10.
- 7 subjects have creatinine levels of 76, 95, 87, 92, 90, 52, and 53, which are much higher than those for the rest of the subjects, who had values in a range of 0.1–10.4. It is believed these were entered in units of μmol/L instead of mg/dL. These creatinine observations are divided by 88.4 to correct the units.

The data after these adjustments are consistent with the information presented in Powell et al. (2010).

Of the $n = 467$ subjects, 446 have complete data on all baseline covariates, and the remainder have missing values for no more than 2 covariates. The missing values were imputed as follows:

- Race is set to "Other" for the 8 subjects for whom it is missing.
- 5 subjects are missing creatinine. A linear model to estimate creatinine using all other baseline covariates was fitted to the data from the complete cases and used to impute the missing values.
- 1 subject is missing white blood cell count (WBC). A linear model to estimate WBC using all baseline covariates except for WBC and Risk group was fitted to the data from the complete cases and used to impute the missing WBC value.
- 4 subjects are missing ECOG performance status. A multinomial logistic regression model for ECOG performance status using all other baseline covariates was fitted to the data from

the complete cases. The missing ECOG performance status values were imputed using this model.

- 4 subjects are missing hemoglobin. A linear model to estimate hemoglobin using all other baseline covariates was fitted to the data from the complete cases and used to impute the missing values.

G.2. Data analysis

At Decision 1, $\mathcal{A}_1 = \{0, 1\}$, where 0 (1) corresponds to ATRA (ATRA + arsenic trioxide) consolidation therapy; at Decision 2, $\mathcal{A}_2 = \{0, 1\}$, where 0 (1) corresponds to ATRA (ATRA+Mtx+MP) maintenance therapy. As in the main paper, the study involves four embedded regimes of the form "Give consolidation therapy a; if subject completes consolidation before the event occurs, give maintenance therapy b," which we denote as Regimes 1, 2, 3, and 4 as $(a, b) = (0, 0), (0, 1), (1, 0), (1, 1)$. To visualize the survival functions $\mathcal{S}(u, d^j) = P\{T^*(d^j) \geqslant u\}$ corresponding to Regimes $j = 1, \ldots, 4$, we estimated the cumulative hazard functions $\Lambda(u, d^j) = \int_0^\infty \lambda(u, d^j)du$ by $\widehat{\Lambda}(u, d^j) = \int_0^\infty \big\{ \sum_{i=1}^n \Omega_i(u, d^j)dN_i(u)/\sum_{i=1}^n \Omega_i(u, d^j)Y_i(u)\big\}$, with estimated randomization probabilities substituted, obtaining estimates $\widehat{\mathcal{S}}(u, d^j) = \exp\{-\widehat{\Lambda}(u, d^j)\}$. Figure 3 depicts $\widehat{\mathcal{S}}(u, d^j)$, $j = 1, \ldots, 4$, and suggests that administering ATRA + arsenic trioxide ($a_1 = 1$) initially, as in Regimes 3 and 4, is more beneficial than administering ATRA alone as in Regimes 1 and 2, consistent with the findings of Powell et al. (2010). The estimates suggest further that, of the regimes starting with ATRA + arsenic trioxide, Regime 4, which gives ATRA+Mtx+MP maintenance to responders, may yield benefit over Regime 3.

[Figure 3 about here.]

At stage 1, let $A_1 = 0$ if a subject was randomized to ATRA consolidation therapy and $A_1 = 1$ if randomized to ATRA+Arsenic Trioxide. Here, a subject is a "nonresponder" if the

subject experienced the event or was censored before completing consolidation therapy, with $\kappa = 1$. A subject is a "responder" if the subject completed consolidation without experiencing the event or censoring, $\kappa = 2$; for such subjects, let $A_2 = 0$ if a subject was randomized at stage 2 to ATRA maintenance therapy and $A_2 = 1$ if randomized to ATRA+Mtx+MP. For consistency with the examples in Web Appendices B and D, define $R_2 = I(\kappa = 2)$, although this definition is redundant, as all subjects for whom $\kappa = 2$ are "responders."

Equal randomization was used at both decisions, i.e., $\omega_1(\boldsymbol{h}_1, a_1) = 1/2$ for all $\boldsymbol{h}_1$ and $a_1 \in \mathcal{A}_1$, and $\omega_2(\boldsymbol{h}_2, a_2) = 1/2$ for all $\boldsymbol{h}_2$, $a_2 \in \mathcal{A}_2$, and $\kappa = 2$. For the proposed methods, as in Web Appendix D, we estimated the randomization probabilities by positing and fitting logistic regression models

$$\omega_1(\boldsymbol{h}_1, a_1; \gamma_1) = \left\{\frac{\exp(\gamma_1)}{1+\exp(\gamma_1)}\right\}^{I(a_1=1)} \left\{\frac{1}{1+\exp(\gamma_1)}\right\}^{I(a_1=0)}$$

at stage 1 and

$$\begin{aligned}\omega_2(\boldsymbol{h}_2, a_2; \boldsymbol{\gamma}_2) = &\left\{\frac{\exp(\gamma_{21})}{1+\exp(\gamma_{21})}\right\}^{I(a_1=0,r_2=1,a_2=1)} \left\{\frac{1}{1+\exp(\gamma_{21})}\right\}^{I(a_1=0,r_2=1,a_2=0)} \\ \times &\left\{\frac{\exp(\gamma_{22})}{1+\exp(\gamma_{22})}\right\}^{I(a_1=1,r_2=1,a_2=1)} \left\{\frac{1}{1+\exp(\gamma_{22})}\right\}^{I(a_1=1,r_2=1,a_2=0)}\end{aligned}$$

at stage 2. Letting $\widehat{\boldsymbol{\gamma}}$ be the ML estimator for $\boldsymbol{\gamma} = (\gamma_1, \gamma_{21}, \gamma_{22})^T$, as in Web Appendix D, to obtain our test statistic without incorporating covariates to gain efficiency, for each regime $j = 1, \ldots, 4$, we performed a linear regression with dependent variable $\widehat{\mathfrak{T}}_i^j(\widehat{\boldsymbol{\gamma}})$ and design matrix with ith row

$$\begin{aligned}\big[\{I(A_{1i}=1) - \text{expit}(\widehat{\gamma}_1)\}, I(A_{1i}=0, R_{2i}=1, \kappa_i=2)\{I(A_{2i}=1) - \text{expit}(\widehat{\gamma}_{21})\}, \\ I(A_{1i}=1, R_{2i}=1, \kappa_i=2)\{I(A_{2i}=1) - \text{expit}(\widehat{\gamma}_{22})\}\end{aligned} \tag{G.1}$$

to obtain the residuals $\widehat{\mathfrak{T}}_i^{j,R}(\widehat{\boldsymbol{\gamma}})$ and thus $\widehat{\boldsymbol{\Sigma}}_\gamma$.

To attempt to enhance power by incorporating covariates, we informally examined associations between the covariates and outcome. To gain a sense of the extent to which components of the baseline covariates $\boldsymbol{X}_1$ in Table 4 are potentially associated with outcome, we fit separate proportional hazards models to the data (U, Δ) for the $n = 467$ subjects,

where in each model we included each component of $\boldsymbol{X}_1$ as the sole covariate. Because the distribution of WBC is extremely skewed, we considered the logarithm of WBC as a covariate, and all discrete covariates were treated as categorical. From these fits, we identified $(X_{11}, X_{12}, X_{13}) = \{\log(\text{WBC}), I(\text{ECOG Performance Status} > 1), I(\text{Risk Group} > 2)\}$ as potentially associated with the EFS outcome. As X_{11} and X_{13} are highly associated owing to the definition of the latter, we opted to consider X_{11} and X_{12} as covariates for the purpose of gaining efficiency as discussed in Web Appendix D. Thus, analogous to (D.10), we defined the vector of basis functions $\widetilde{\boldsymbol{H}}_1 = (X_{11}, X_{12})^T$.

To gain an informal sense of the extent to which adverse event variables in $\boldsymbol{X}_2$ are associated with outcome, we considered only the 310 subjects who were observed to reach the second decision point ($\kappa = 2$), and fit separate proportional hazards models to the data (U, Δ) including each component of $\boldsymbol{X}_2$ as the sole covariate, treating each covariate as categorical. From these fits, we identified the binary variables $X_{21} =$ I_10028813, Nausea, and $X_{22} =$ IC_90004060, hemorrhage or bleeding with grade 3 or 4 neutropenia, as potentially associated with the EFS outcome. Thus, analogous to (D.10), we defined vectors of basis functions $\widetilde{\boldsymbol{H}}_{21} = \widetilde{\boldsymbol{H}}_{22} = (X_{11}, X_{12}, X_{21}, X_{22})^T$, appended to the ith row of the design matrix in (G.1) the additional components

$$\big[\{I(A_{1i}=1) - \text{expit}(\widehat{\gamma}_1)\}\widetilde{\boldsymbol{H}}_1^T, I(A_{1i}=0, R_{2i}=1, \kappa_i=2)\{I(A_{2i}=1) - \text{expit}(\widehat{\gamma}_{21})\}\widetilde{\boldsymbol{H}}_{21}^T,$$
$$I(A_{1i}=1, R_{2i}=1, \kappa_i=2)\{I(A_{2i}=1) - \text{expit}(\widehat{\gamma}_{22})\}\widetilde{\boldsymbol{H}}_{22}^T$$

and carried out the regression with dependent variable $\widehat{\mathfrak{T}}_i^j(\widehat{\boldsymbol{\gamma}})$ and obtained the residuals $\widehat{\mathfrak{T}}_i^{j,R}(\widehat{\boldsymbol{\gamma}})$ and thus $\widehat{\boldsymbol{\Sigma}}_\gamma$ and the test statistic $\mathbb{Z}(\widehat{\boldsymbol{\gamma}})$..

The maximum follow-up time is 4694 days, with maximum time to EFS of 4000; in all analyses, we took $L = 4200$, resulting in about 2% truncation.

References

Alam, S., Moodie, E. E. M., and Stephens, D. A. (2018). Should a propensity score model be super? the utility of ensemble procedures for causal adjustment. *Statistics in Medicine* **38,** 1690–1702.

Ertefaie, A., Hejazi, N. S., and var der Laan, M. J. (2023). Nonparametric inverse probability-weighted estimators based on the highly adaptive lasso. *Biometrics* **79,** 1029–1041.

Hager, R., Tsiatis, A. A., and Davidian, M. (2018). Optimal two-stage dynamic treatment regimes from a classification perspective with censored survival data. *Biometrics* **74,** 1180–1192.

Kauermann, G. and Carroll, R. J. (2001). A note on the efficiency sandwich matrix estiamtion. *Journal of the American Statistical Association* **96,** 1387–1396.

Kidwell, K. M. and Wahed, A. S. (2013). Weighted log-rank statistic to compare shared-path adaptive treatment strategies. *Biostatistics* **14,** 299–312.

Lee, B. K., Lessler, J., and Stuart, E. A. (2010). Improving propensity score weighting using machine learning. *Statistics in Medicine* **29,** 337–346.

Li, Z., Valenstein, M., Pfeiffer, P., and Ganoczy, D. (2014). A global logrank test for adpative treatment strategies based on observational studies. *Statistics in Medicine* **33,** 760–771.

McCaffrey, D. F., Ridgeway, G., and Morral, A. R. (2004). Propensity score estimation with boosted regression for evaluating causal effects in observational studies. *Psychological Methods* **9,** 403–425.

Pirracchio, R., Petersen, M. L., and van der Laan, M. (2015). Improving propensity score estimators' robustness to model misspecification using super learner. *American Journal of Epidemiology* **181,** 108–119.

Powell, B. L., Moser, B., Stock, W., Gallegher, R. E., Willman, C. L., Stone, R. M., Rowe, J. M., Coutre, S., Feusner, J. H., Gregory, Couban, S., Appelbaum, F. R., Tallman, M. S.,

and Larson, R. A. (2010). Arsenic trioxide improves event-free and overall survival for adults with acute promyelocytic leukemia: North American Leukemia Intergroup Study C9710. *Blood* **116,** 3751–3757.

Schaubel, D. E. (2005). Variance estimation for clustered recurrent event data with a small number of clusters. *Statistics in Medicine* **24,** 3037–3051.

Tsiatis, A. A. (2006). *Semiparametric Theory and Missing Data*. Springer, New York.

Tsiatis, A. A. and Davidian, M. (2022). Estimating vaccine efficacy over time after a randomized study is unblinded. *Biometrics* **78,** 825–838.

Tsiatis, A. A., Davidian, M., Holloway, S. T., and Laber, E. B. (2020). *Dynamic Treatment Regimes: Statistical Methods for Precision Medicine*. Chapman and Hall/CRC Press, Boca Raton, FL.

var der Laan, M. J., Benkeser, D., and Cai, W. (2022). Efficient estimation of pathwise differentiable target parameters with the undersmoothed highly adaptive lasso. *International Journal of Biostatistics* **19,** 261–289.

Wang, X., Turner, E. L., and Li, F. (2023). Improving sandwich variance estimation for marginal cox analysis of cluster randomized trials. *Biometrical Journal* **65,** 2200113.

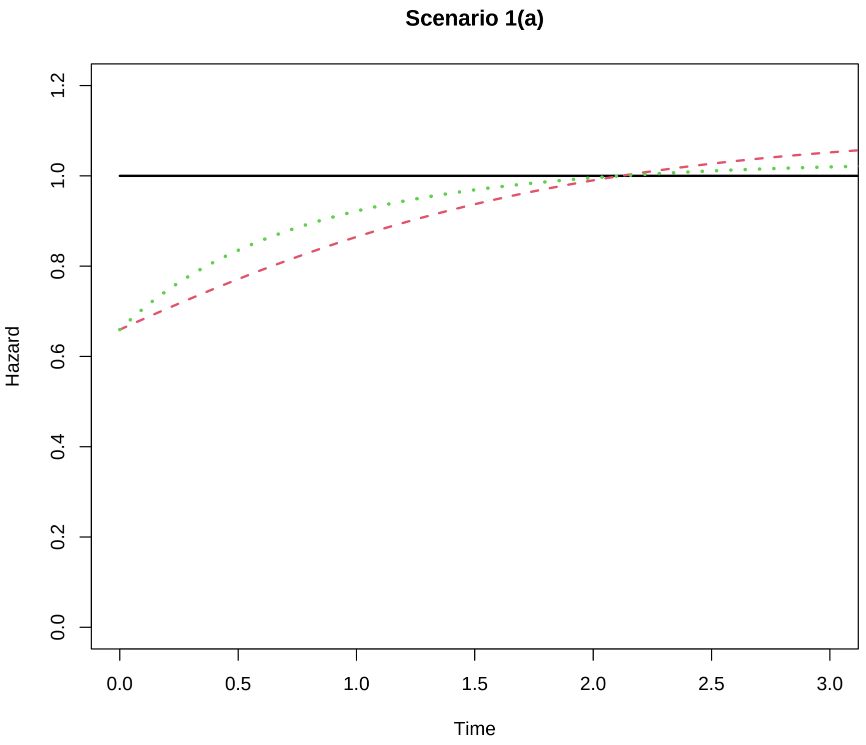

Figure 1. Hazards under Scenario 1(a). The solid line is λ_3, the dashed line is (F.1), and the dotted line is (F.2).

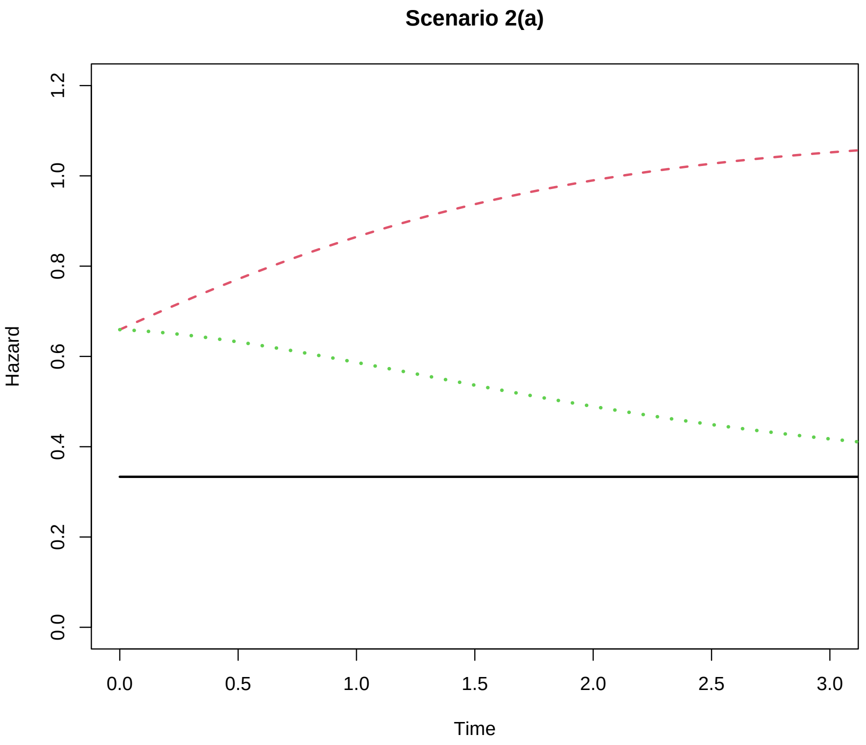

Figure 2. Hazards under Scenario 2(a). The solid line is λ_3, the dashed line is (F.1), and the dotted line is (F.2).

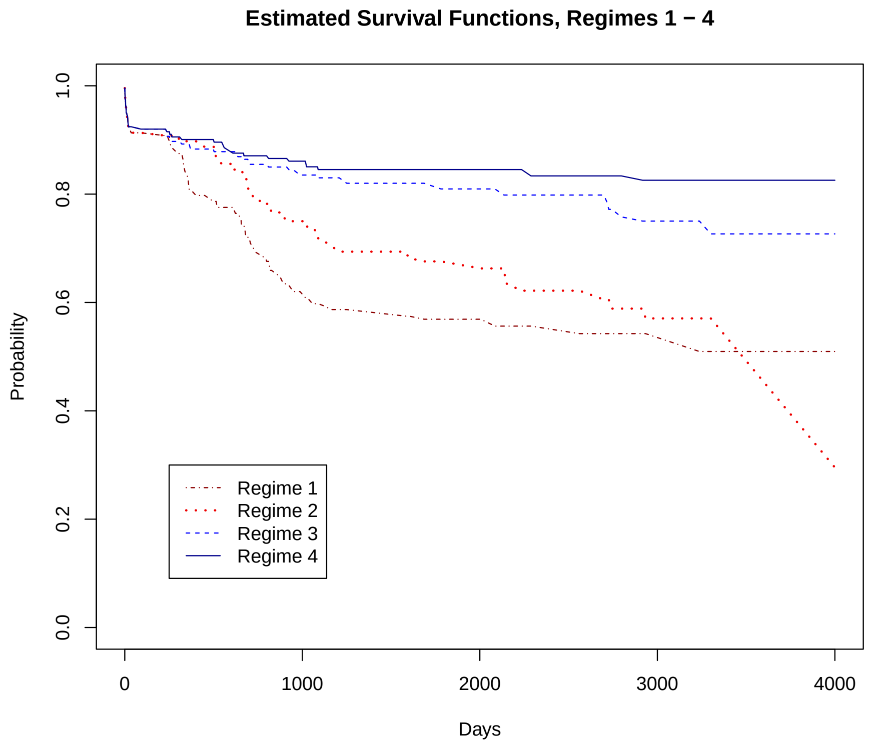

Figure 3. Estimates of the survival distributions $\mathcal{S}(u, d^j)$ corresponding to embedded Regimes $j = 1, \ldots, 4$, shown with dash-dotted, dotted, dashed, and solid lines, respectively. As in the text, the regimes are of the form "Give consolidation therapy a; if subject completes consolidation before the event occurs, give maintenance therapy b," where Regimes 1, 2, 3, 4 take $(a, b) = (0, 0), (0, 1), (1, 0), (1, 1)$, respectively.

Table 1

Simulation results for comparisons of pairs of embedded regimes under data generative scenarios described in the main paper under both the null hypothesis H_0 in (??) and alternatives based on 5000 Monte Carlo (MC) data sets, with all tests conducted with level of significance 0.05. Entries are as in Table ?? of the main paper except that $\mathbb{Z}_{KW}$ denotes the test based on the relevant component of the test statistic proposed by Kidwell and Wahed (2013). For each scenario, "3v4" denotes the "shared path" comparison of regimes 3 and 4 (first component of the test of Kidwell and Wahed (2013) and "xv4" denotes the "non-shared path" comparison of regimes x and 4 (second/third component of the test of Kidwell and Wahed (2013)).

**Scenario**	n	ζ	$\mathbb{Z}_{\text{U,nocov}}$	$\mathbb{Z}_{\text{C,nocov}}$	$\mathbb{Z}_{\text{U,cov}}$	$\mathbb{Z}_{\text{C,cov}}$	$\mathbb{Z}_{\text{KW}}$
				**Null Scenarios**			
1(a) 3v4	250	–	0.066	0.048	0.064	0.048	0.056
	500	–	0.058	0.047	0.056	0.046	0.050
	1000	–	0.057	0.051	0.057	0.053	0.052
1(a) 2v4	250	–	0.068	0.057	0.067	0.056	0.060
	500	–	0.054	0.047	0.056	0.051	0.051
	1000	–	0.052	0.050	0.052	0.050	0.052
1(a) 1v4	250	–	0.062	0.054	0.065	0.054	0.057
	500	–	0.057	0.051	0.056	0.050	0.056
	1000	–	0.055	0.051	0.053	0.050	0.052
2(a) 3v4	250	–	0.068	0.048	0.068	0.051	0.090
	500	–	0.060	0.051	0.060	0.050	0.085
	1000	–	0.053	0.047	0.051	0.046	0.080
2(a) 2v4	250	–	0.061	0.047	0.067	0.054	0.055
	500	–	0.056	0.048	0.057	0.052	0.053
	1000	–	0.052	0.048	0.053	0.051	0.049
2(a) 1v4	250	–	0.058	0.049	0.062	0.051	0.054
	500	–	0.058	0.050	0.061	0.053	0.053
	1000	–	0.053	0.049	0.057	0.053	0.053
3(a), 3v4	250	0.00	0.063	0.050	0.060	0.049	0.053
	500	0.00	0.050	0.045	0.051	0.045	0.047
	1000	0.00	0.052	0.049	0.056	0.043	0.046
3(a), 2v4	250	0.00	0.058	0.049	0.056	0.044	0.056
	500	0.00	0.055	0.052	0.053	0.049	0.055
	1000	0.00	0.051	0.046	0.054	0.052	0.054
3(a), 1v4	250	0.00	0.056	0.047	0.057	0.049	0.053
	500	0.00	0.053	0.049	0.057	0.051	0.053
	1000	0.00	0.054	0.051	0.051	0.047	0.056
3(c), 3v4	250	0.00	0.059	0.046	0.060	0.0.046	0.053
	500	0.00	0.054	0.048	0.050	0.045	0.055
	1000	0.00	0.051	0.047	0.056	0.050	0.057
3(c), 2v4	250	0.00	0.055	0.048	0.058	0.049	0.073
	500	0.00	0.050	0.046	0.051	0.045	0.073
	1000	0.00	0.052	0.049	0.051	0.049	0.080
3(c), 1v4	250	0.00	0.057	0.050	0.058	0.050	0.070
	500	0.00	0.055	0.049	0.056	0.051	0.075
	1000	0.00	0.047	0.045	0.048	0.046	0.074
				**Alternative Scenarios**			
3(a), 3v4	1000	1.75	0.195	0.188	0.242	0.234	0.147
3(a), 2v4	1000	1.75	0.581	0.570	0.678	0.671	0.563
3(a), 1v4	1000	1.75	0.845	0.840	0.915	0.911	0.848
3(c), 3v4	1000	3.50	0.625	0.614	0.766	0.755	0.476
3(c), 1v4	1000	3.50	0.340	0.334	0.444	0.435	0.343

Table 2

*Simulation results for comparisons of a set of three arbitrary regimes under the null hypothesis H_0 in (**??**) based on 5000 Monte Carlo (MC) data sets, with all tests conducted with level of significance 0.05. Entries are as in Table **??** of the main paper.*

**Scenario**	n	ζ	$\mathbb{Z}_{\mathrm{U,nocov}}$	$\mathbb{Z}_{\mathrm{C,nocov}}$	$\mathbb{Z}_{\mathrm{U,cov}}$	$\mathbb{Z}_{\mathrm{C,cov}}$
			**Null Scenario**			
3(a)	250	0.00	0.056	0.045	0.062	0.049
	500	0.00	0.054	0.049	0.053	0.048
	1000	0.00	0.051	0.048	0.053	0.050

Table 3

*Simulation results for comparisons of sets of regimes in the three decision SMART scenario under the null hypothesis H_0 in (**??**) based on 5000 Monte Carlo (MC) data sets, with all tests conducted with level of significance 0.05. The first column indicates the set of regimes $\mathcal{D}$ and thus the comparison of interest; "All" indicates that $\mathcal{D}$ corresponds to all eight embedded regimes. Otherwise, entries are as in Table **??** of the main paper.*

**Comparison**	n	ζ	$\mathbb{Z}_{\text{U,nocov}}$	$\mathbb{Z}_{\text{C,nocov}}$	$\mathbb{Z}_{\text{U,cov}}$	$\mathbb{Z}_{\text{C,cov}}$
			**Null Scenario**			
All	1000	0.00	0.072	0.067	0.071	0.063
	1500	0.00	0.058	0.056	0.067	0.061
	2000	0.00	0.059	0.057	0.059	0.055
	4000	0.00	0.057	0.056	0.054	0.053
1,8	500	0.00	0.060	0.053	0.059	0.049
	1000	0.00	0.053	0.049	0.051	0.047
	1500	0.00	0.051	0.048	0.049	0.044
1,2	500	0.00	0.062	0.052	0.066	0.054
	1000	0.00	0.055	0.049	0.057	0.048
	1500	0.00	0.047	0.043	0.054	0.048
1,5	500	0.00	0.067	0.054	0.065	0.045
	1000	0.00	0.056	0.049	0.059	0.048
	1500	0.00	0.058	0.051	0.054	0.047
1,2,5,6	1000	0.00	0.067	0.062	0.066	0.057
	1500	0.00	0.058	0.054	0.054	0.048

Table 4
Baseline covariates available from the North American Leukemia Intergroup Study C9710. Race is redefined as indicated in the text.

Variable	Meaning
Age	Age at registration
Gender	1=Male, 2=Female
Race	1=White, 2=Hispanic American, 3=Black/African American, 9=Other
Ethnicity	1=Hispanic, 2=Non-Hispanic, 9=Unknown
WBC	White blood cell count at registration ($10^3/\mu$L)
Platelet	Platelet count at registration ($10^3/\mu$L)
ECOG Performance Status	0 = fully active, 1= restricted in strenuous activity, 2 = restricted in work activity, 3 = capable of limited self care, 4 = completely disabled
Risk Group	1=Low (WBC $\leqslant$ 10 and Platelet $>$ 40), 2=Intermediate (WBC $\leqslant$ 10 and Platelet $\leqslant$ 40), 3=High (WBC $>$ 10)
Creatinine	Serum creatinine (mg/dL)
Hemoglobin	Hemoglobin (g/dL)

Table 5
medDRA codes and definitions of the adverse events experienced by at least 5% of patients in North American Leukemia Intergroup Study C9710.

medDRA	Adverse Event Category	Adverse Event
10002646	Gastrointestinal	Anorexia
10012457	Dermatology/skin	Rash/desquamation
10012745	Gastrointestinal	Diarrhea
10013442	Coagulation	Disseminated intravascular coagulation
10013972	Pulmonary	Dyspnea (shortness of breath)
10016288	Infection	Febrile neutropenia, fever of unknown origin without clinically or microbiologically documented infection (ANC $<$1.0 x 10e9/L, fever $\geqslant$ 8.5 degrees C)
10018876	Blood/bone marrow	Hemoglobin
10019218	Pain	Headache
10020637	Metabolic/laboratory	Glucose serum-high (hyperglycemia)
10020947	Metabolic/laboratory	Calcium serum-low (hypocalcemia)
10021015	Metabolic/laboratory	Potassium serum-low (hypokalemia)
10021143	Pulmonary/upper respiratory	Hypoxia
10021842	Infection/febrile neutropenia	Infection without neutropenia
10024285	Blood/bone marrow	Leukocytes (total WBC)
10025327	Blood/bone marrow	Lymphopenia
10028813	Gastrointestinal	Nausea
10029363	Blood/bone marrow	Neutrophils/granulocytes (ANC/AGC)
10033359	Blood/bone marrow	Transfusion: packed red blood cells
10035528	Blood/bone marrow	Platelets
10035543	Blood/bone marrow	Transfusion: platelets
10043607	Vascular	Thrombosis/thrombus/embolism
90004060	Hemorrhage	Hemorrhage/bleeding with grade 3 or 4 thrombocytopenia
90004070	Infection/febrile neutropenia	Infection (documented clinically or microbiologically) with grade 3 or 4 neutropenia (ANC $<$1.0 x 10e9/L)
